# Tuning of Oxidation Potential of Ferrocene for Ratiometric Redox Labeling and Coding of Nucleotides and DNA

**DOI:** 10.1002/chem.201904700

**Published:** 2020-01-09

**Authors:** Anna Simonova, Ivan Magriñá, Veronika Sýkorová, Radek Pohl, Mayreli Ortiz, Luděk Havran, Miroslav Fojta, Ciara K. O'Sullivan, Michal Hocek

**Affiliations:** ^1^ Institute of Organic Chemistry and Biochemistry Czech Academy of Sciences Flemingovo namesti 2 16610 Prague 6 Czech Republic; ^2^ Department of Organic Chemistry Faculty of Science Charles University in Prague Hlavova 8 Prague-2 12843 Czech Republic; ^3^ Departament d'Enginyeria Química Universitat Rovira i Virgili 26 Països Catalans 43007 Tarragona Spain; ^4^ Institute of Biophysics of the Czech Academy of Sciences Královopolská 135 61265 Brno Czech Republic; ^5^ Central European Institute of Technology Masaryk University Kamenice 753/5 62500 Brno Czech Republic; ^6^ Institució Catalana de Recerca i Estudis Avançats Passeig Lluís Companys, 23 08010 Barcelona Spain

**Keywords:** DNA, electrochemistry, ferrocenes, nucleobases, redox labelling

## Abstract

Three sets of 7‐deazaadenine and cytosine nucleosides and nucleoside triphosphates bearing either unsubstituted ferrocene, octamethylferrocene and ferrocenecarboxamide linked through an alkyne tether to position 7 or 5, respectively, were designed and synthesized. The modified **dN^FcX^TP**s were good substrates for KOD XL DNA polymerase in primer extension and were used for enzymatic synthesis of redox‐labelled DNA probes. Square‐wave voltammetry showed that the octamethylferrocene oxidation potential was shifted to lower values, whilst the ferrocenecarboxamide was shifted to higher potentials, as compared to ferrocene. Tailed PEX products containing different ratios of Fc‐labelled A (**dA^Fc^**) and FcPa‐labelled C (**dC^FcPa^**) were synthesized and hybridized with capture oligonucleotides immobilized on gold electrodes to study the electrochemistry of the redox‐labelled DNA. Clearly distinguishable, fully orthogonal and ratiometric peaks were observed for the **dA^Fc^** and **dC^FcPa^** bases in DNA, demonstrating their potential for use in redox coding of nucleobases and for the direct electrochemical measurement of the relative ratio of nucleobases in an unknown sequence of DNA.

## Graphical abstract

**Orthogonal redox labelling** of nucleobases with substituted ferrocenes with tuned redox potential was developed for redox coding of DNA bases.

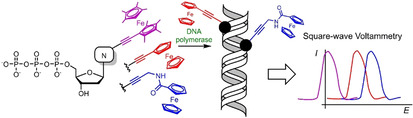

## Introduction

Redox labelling of DNA bases by attachment of some oxidisable or reducible groups is used for diverse applications in bioanalysis and diagnostics.^1^, ^2^ The redox active group can be attached to 2′‐deoxyribonucleoside triphosphate (dNTP) and used for polymerase mediated synthesis of redox‐labelled DNA for subsequent electrochemical detection^2^ with further possible applications for construction of electrochemical genosensors.^3^ By using a combination of several orthogonal redox labels with differing redox potentials, an attractive option of redox coding of DNA bases^4^ can be envisaged for applications in sequencing. Previously, we and others have reported the use of nitrophenyl,^5^ anthraquinone,^4^, ^6^ benzofurazane^7^ and azidophenyl^8^ as reducible labels, some of which (combination of nitrophenyl with either benzofurazane or azidophenyl) were orthogonal and suitable for ratiometric redox coding of two bases, but none of the organic oxidizable labels, that is, aminophenyl,^5^ methylene blue,^4^, ^9^ methoxyphenol,^10^ or phenothiazines,^4^, ^11^ was truly ideal and orthogonal for combination with another label(s). Ferrocene is a classical electrochemical standard^12^ that has been used as an oxidizable electrochemical label for nucleotides and DNA^13^, ^14^ and has been exploited in sensors.^3^ In order to develop a set of fully orthogonal oxidizable redox labels for the use in redox coding, herein we report the fine tuning of the oxidation potential of ferrocene by its substitution with electron‐donating or electron‐withdrawing substituents.

## Results and Discussion

### Synthesis

In our design of modified ferrocene labels, we envisaged that multiple methylation of ferrocene, as in octamethylferrocene, can be used as electron‐donating substituents to decrease the redox potential, whereas a substitution with an electron‐withdrawing carboxamide should increase the oxidation potential. To easily synthesise the modified ferrocene‐linked nucleosides and nucleotides through direct aqueous‐phase Sonogashira cross‐coupling reactions,^15^ we designed the corresponding terminal alkynes: 1‐ethynyl‐1′,2,2′,3,3′,4,4′,5‐octamethylferrocene (**FcMe**)^16^ and propargylaminocarbonylferrocene (**FcPa**)^17^ as suitable building blocks and prepared them as previously reported.

**Scheme 1:**
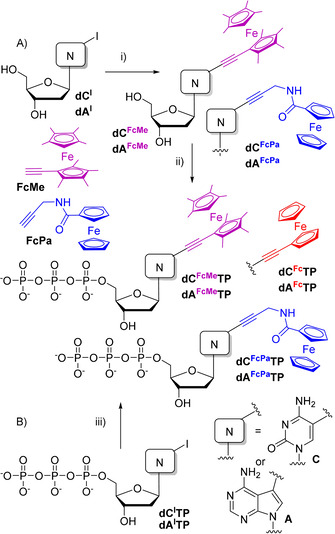
Synthesis of modified nucleosides and dNTPs. Reagents and conditions: i) **FcPA** or **FcMe**, Pd(PPh_3_)_2_Cl_2_+PPh_3_ or Pd(OAc)_2_+TPPTS, CuI, PPh_3_, DMF (75 °C, 1 h) or MeCN/H_2_O (1:1) (50 °C, 40 min.); ii) 1. POCl_3_, PO(OMe)_3_, 0 °C, 3 h; 2. (NHBu_3_)_2_H_2_P_2_O_7_, Bu_3_N, DMF, 0 °C, 1.5 h; 3. TEAB; iii) **FcPA** or **FcMe**, Pd(PPh_3_)_2_Cl_2_, CuI, PPh_3_, MeCN/H_2_O (1:1), 60 °C, 1 h.

The modified ferrocene‐linked nucleosides were synthesized by Sonogashira cross‐coupling reactions of unprotected halogenated nucleosides (**dA^I^** or **dC^I^**) in the presence of Pd(PPh_3_)_2_Cl_2_ or Pd(OAc)_2_ catalyst, PPh_3_ or TPPTS (triphenylphosphine‐3,3′,3“‐trisulfonate) ligand, CuI and Et_3_N either in MeCN/water or in DMF to give labelled nucleosides **dN^FcMe^** or **dN^FcPa^** in high yields (Table 1, Scheme 1).

**Table 1:** Synthesis of nucleosides and dNTPs bearing acetylene‐linked ferrocene labels.

Starting compound	Reagent	Catalyst	Solvent	Base	Product	Yield [%]
**dA^I^**	**FcMe**	Pd(OAc)_2_, CuI, TPPTS	MeCN/H_2_O (1/1)	Et_3_N	**dA^FcMe^**	90
**dC^I^**					**dC^FcMe^**	86
**dA^I^**	**FcPa**	Pd(PPh_3_)_2_Cl_2_, CuI, PPh_3_	DMF	Et_3_N	**dA^FcPa^**	98
**dC^I^**					**dC^FcPa^**	97
**dA^I^TP**	**FcMe**	Pd(PPh_3_)_2_Cl_2_, CuI, PPh_3_	MeCN/H_2_O (1/1)	Et_3_N	**dA^FcMe^TP**	38^[a]^
**dC^I^TP**					**dC^FcMe^TP**	30^[**a]**^
**dA^I^TP**	**FcPA**	Pd(PPh_3_)_2_Cl_2_, CuI, PPh_3_	MeCN/H_2_O (1/1)	Et_3_N	**dA^FcPa^TP**	13
**dC^I^TP**					**dC^FcPa^TP**	16
**dC^I^TP**	ethynylferrocene	Pd(OAc)_2_, CuI, TPPTS	MeCN/H_2_O (1/1)	Et_3_N	**dC^Fc^TP**	27
**dA^FcPa^**	–^[b]^				**dA^FcPa^TP**	22
**dC^FcPa^**	–^[b]^				**dC^FcPa^TP**	18
**dA^FcMe^**	–^[b]^				**dA^FcMe^TP**	15^[a]^
**dC^FcMe^**	–^[b]^				**dC^FcMe^TP**	20^[a]^

[a] isolated **dN^FcMe^TP**s were prone to oxidation on air. [b] Reaction conditions: 1) PO(OMe)_3_, POCl_3_, 0 °C; 2) (NHBu_3_)_2_H_2_P_2_O_7_, Bu_3_N, DMF, 0 °C; 3) TEAB (2 m).

The corresponding modified dNTPs were prepared (Scheme 1, Table 1) either by phosphorylation of modified nucleosides (Approach A) or by direct aqueous‐phase cross‐coupling reactions of halogenated triphosphates **dN^I^TP** with Fc‐alkynes (Approach B). The cross‐coupling reactions were performed analogously to the reaction of nucleosides using Pd(PPh_3_)_2_Cl_2_ catalyst in acetonitrile/water for 1 h. The reactions reached completion but, due to partial hydrolysis of the triphosphates and difficulties in separation, the desired **dN^FcMe^TP** or **dN^FcPa^TP** were isolated in moderate yields (13–38 %) after isolation by HPLC. The alternative approach using triphosphorylation of nucleosides gave similarly moderate yields. In all cases, the modified **dN^FcX^TPs** were prepared and isolated in sufficient quantities for the subsequent biochemical and electrochemical experiments. The dNTPs bearing unsubstituted ethynylferrocene were also synthesised for comparison. **dA^Fc^TP**s was prepared as previously reported^14^ and the related **dC^Fc^TP** was prepared analogously through Sonogashira reaction of **dC^I^TP** with ethynylferrocene achieving a yield of 27 %.

### Biochemistry

**Figure 1:**
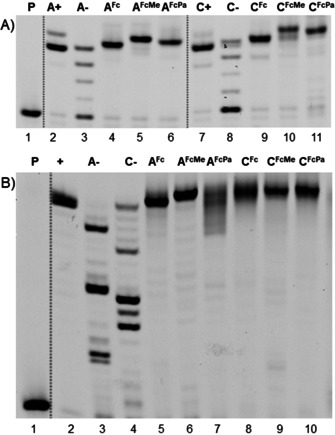
Primer extension with a KOD XL polymerase using either (A) 19‐mer template temp^*A*^ or temp^*C*^ and prim^*rnd*^; or (B) 31‐mer temp^*rnd16*^ and prim^*rnd*^: (P) primer (5′‐6‐FAM‐labelled); (A+, C+ or +) natural dNTPs; (A‐) dCTP, dTTP, dGTP; (C‐) dATP, dTTP, dGTP; (A^Fc^) **dA^Fc^TP**, dCTP, dTTP, dGTP; (C^Fc^) **dC^Fc^TP**, dATP, dTTP, dGTP; (A^FcMe^) **dA^FcMe^TP**, dCTP, dTTP, dGTP; (C^FcMe^) **dC^FcMe^TP**, dATP, dTTP, dGTP; (A^FcPa^) **dA^FcPa^TP**, dCTP, dTTP, dGTP; (C^FcPa^) **dC** ^FcPa^ **TP**, dATP, dTTP, dGTP.

The Fc‐modified **dN^FcX^TP**s were then tested as substrates for DNA polymerases in primer extension (PEX) reactions using either a 19‐mer (temp^*A*^ or temp^*C*^) or 31‐mer (temp^*rnd16*^) template and 15‐mer primer prim^*rnd*^ (for sequences of all oligonucleotides, see Table S1 in Supporting Information). KOD XL DNA polymerase was selected based on previous reports^7^, ^8^, ^11^, ^18^ on extensive use of KOD DNA polymerase and its mutants for polymerase synthesis of base‐modified DNA. Figure 1 shows the PAGE analysis confirming that in all cases, full‐length PEX products containing either 1 or 4 modified nucleotides were formed. The identity of most of the PEX products was also confirmed by MALDI analysis (Table 2) of single‐stranded oligonucleotides (ssONs) after PEX with biotinylated template and magnetoseparation. Only in the case of 31‐mers containing 4 **dN^FcMe^** bases the mass of the full‐length products, was not observed, probably due to the limited stability of the octamethylferrocene label, which is prone to oxidation in air. We also studied the **dN^FcX^TP**s as nucleotide building blocks for PCR, however, similarly to the previously reported **dA^Fc^TP**,^14^ the PCR amplification in the absence of natural dATP (or dCTP) did not work (Figure S13a in Supporting Information). On the other hand, when using the modified **dN^FcX^TP**s in presence of the natural dNTP (ratio 60:40), we observed formation of partially labelled amplicons (Figure S13b in Supporting Information), which can be used for electrochemical detection of the PCR products similarly as in our recent work on Fc‐based electrochemical genosensor.^3^

**Table 2:** List of MALDI data of PEX products bearing modified or non‐modified Fc labels.

oligonucleotide	*M* calcd [Da]	*M* found [Da]
**31ON** **4A^Fc^**	10 445.5	10 447.0
**31ON** **4A^FcPa^**	10 673.7	10 674.1
**31ON** **4C^Fc^**	10 449.5	10 451,9
**31ON** **4C^FcPa^**	10 677.7	10 679.6
**19ON** **1A^Fc^**	6182.0	6183.2
**19ON** **1A^FcPa^**	6239.0	6238.6
**19ON** **1A^FcMe^**	6294.0	6295.4
**19ON** **1C^Fc^**	6159.0	6160.2
**19ON** **1C^FcPa^**	6216.0	3389.4
**19ON** **1C^FcMe^**	6271.0	6272.3

### Electrochemistry of nucleosides and nucleotides

**Figure 2:**
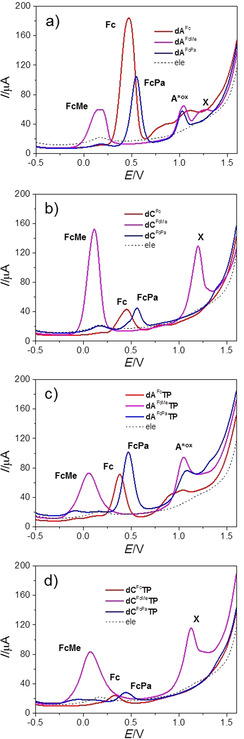
Square‐wave voltammograms of modified nucleosides (a, b) or 40 μm dNTPs (c, d) measured at a pyrolytic graphite electrode in 0.2 m acetate buffer (pH 5.0).

The electrochemical behavior of Fc‐modified nucleosides and dNTPs was studied using Square‐wave voltammetry (SWV) on a pyrolytic graphite electrode (PGE) in acetate buffer (pH 5.0). Samples of nucleosides **dN^FcX^** and triphosphates **dN^FcX^TP** show voltammetric peaks corresponding to reversible oxidation of the ferrocene moiety (see Figure S14–15 in Supporting Information for the evidence of signal reversibility/irreversibility given by components of the SWV current). In the case of 7‐deazaadenine derivatives, an additional peak of irreversible oxidation of the pyrrolopyrimidine moiety was observed at 1.03–1.10 V (vs. Ag/AgCl). As expected and designed, the substitution of ferrocene strongly influences the oxidation potential. The electron rich octamethylferrocene is shifted to lower oxidation potentials by ca. 300 mV (Table 3). This easier oxidation, however, leads to limited stability of these labels in air. On the other hand, the electron‐poor amide‐linked ferrocene derivatives are shifted to higher potentials by ca. 100 mV. Compounds containing octamethylferrocene give an additional irreversible peak (denoted as X in Figure 2) at 1.20 V.

**Table 3:** Redox potentials of FcX‐labelled nucleosides and dNTPs.^[a]^

Compound	FcX [V]	A*^ox^[V]	X [V]
**dA^Fc^**	0.470	1.100	
**dA^FcMe^**	0.160	1.040	1.280
**dA^FcPa^**	0.550	1.030	
**dA^Fc^TP**	0.380	1.030	
**dA^FcMe^TP**	0.060	1.050	–
**dA^FcPa^TP**	0.470	1.080	
**dC^Fc^**	0.325		
**dC^FcMe^**	0.115		1.200
**dC^FcPa^**	0.550		
**dC^Fc^TP**	0.325		
**dC^FcMe^TP**	0.070		1.125
**dC^FcPa^TP**	0.440

[a] Peak potentials of net SWV signals measured at the PGE against Ag|AgCl|3 m KCl. For more details see Figure 2.

### Design, synthesis and electrochemistry of modified DNA probes

**Scheme 2:**
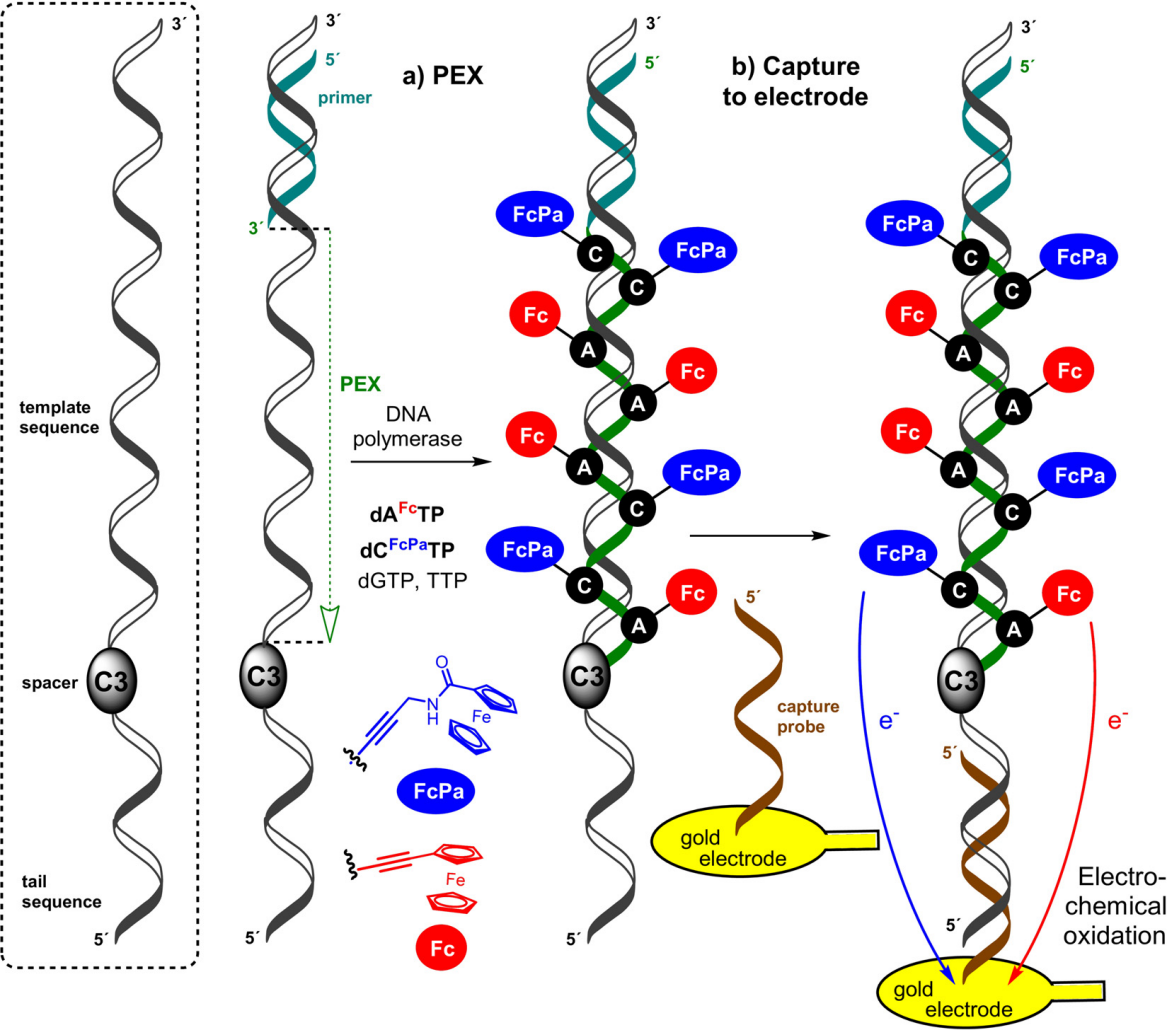
PEX synthesis of the modified DNA probes and their capture on electrode.

In order to study the electrochemistry of redox‐labelled DNA, we initially tried to synthesize FcX‐modified ssONs by PEX with magnetoseparation and studied their SWV on carbon paste electrodes (analogously to our previous works^14^). Unfortunately, we did not observe any significant signals, probably because of low amounts of the modified ssONs and problems with their adsorption on electrodes. Therefore, we applied our recently reported approach^3^ based on the synthesis of tailed‐PEX products and their capture on gold electrodes (Scheme 2). Since the octamethylferrocene‐labelled nucleotides and ONs were prone to oxidation with air, we focused only on the stable unsubstituted ferrocene (**Fc**) and carboxamidoferrocene (**FcPa**) labels.

To test whether the **Fc** and **FcPa** labels can be distinguished and electrochemically quantified, we designed two 55‐mer and one 37‐mer template bearing a 20‐mer ON sequence at the 3′‐end separated through a ‐(CH_3_)_3_‐ (C3) spacer (Table S1). The two 55‐mer tailed templates were designed for the synthesis of PEX products containing either a combination of 8 **dA^Fc^** and 2 **dC^FcPa^** (**DNA 8A^Fc^** **2C^FcPa^**) or a combination of 2 **dA^Fc^** and 8 **dC^FcPa^** (**DNA 2A^Fc^** **8C^FcPa^**). The shorter 37‐mer template was designed for PEX synthesis of DNA containing equimolar numbers of 4 **dA^Fc^** and 4 **dC^FcPa^** (**DNA 4A^Fc^** **4C^FcPa^**). The role of the 3′‐tail was to facilitate hybridization of the PEX product to a complementary capture probe immobilised on the gold electrode and the role of the C3 spacer was to prevent further extension during the PEX, maintaining the tail as ssON.

**Figure 3:**
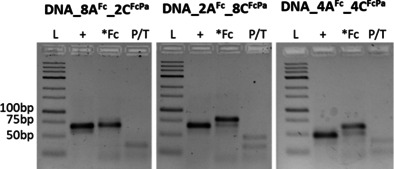
Agarose gel electrophoresis of PEX products using temp^*8A*, *2C*^, temp^*8C*, *2A*^ or temp^*4C*, *4A*^ templates and Primer^8/2 4/4^ obtained in presence of (a) all four natural dNTPs (+), or combination of **dA^Fc^TP**, **dC^FcPa^TP** with TTP and GTP (*Fc). P/T corresponds to primer hybridized to template in the absence of polymerase.

Agarose gel electrophoresis (Figure 3) shows the PEX product formation using either set of natural dNTPs (+) or combination of **dA^Fc^TP**, **dC^FcPa^TP** with TTP and GTP (*Fc). In all cases, the formation of the full‐length PEX product was observed confirming that even the combination of two Fc‐modified dNTPs can be used for polymerase construction of double‐redox‐labelled DNA. The labelled PEX products were then hybridized to the complementary capture probe immobilised on gold electrode, washed and used for electrochemical interrogation by SWV. Ca(NO_3_)_2_ was selected as the electrolyte solution due to the ability of divalent cations to shrink, compact and bend DNA, whilst also shielding electrostatic repulsions between neighbour DNA strands.^19^

**Figure 4:**
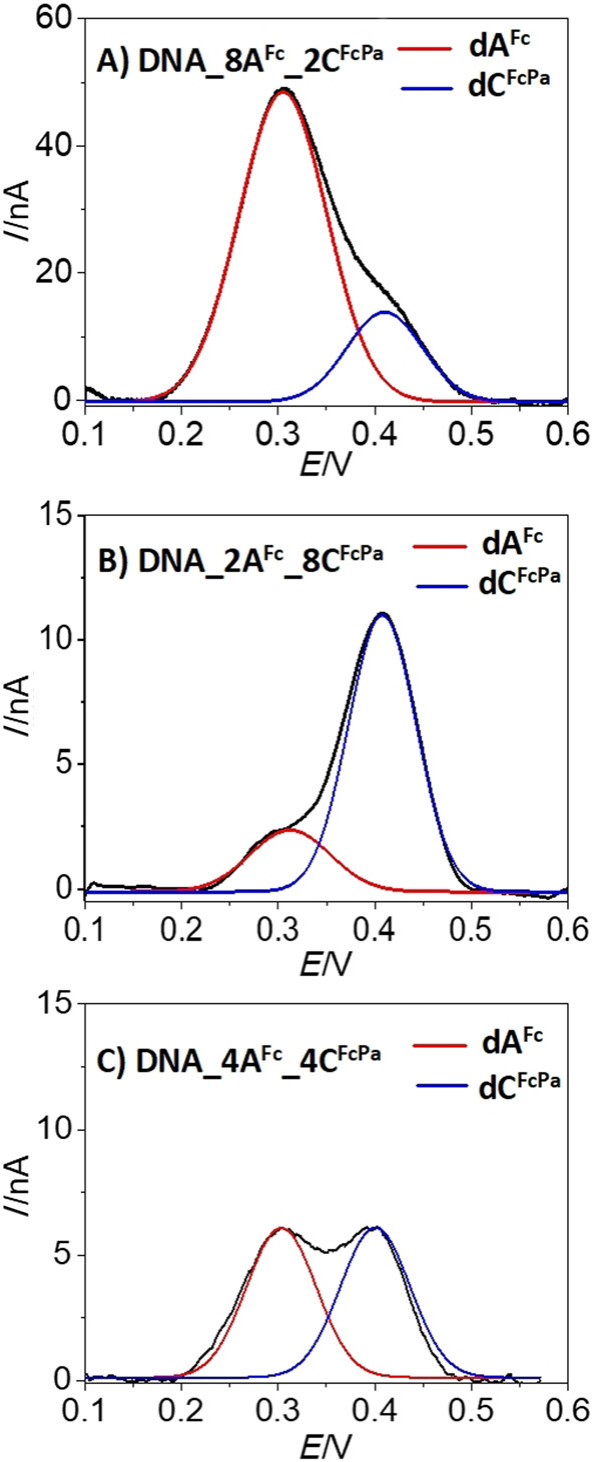
Square wave voltammograms of the oxidation peaks of **dA^Fc^** and **dC^FcPa^** on PEX products and then hybridized to a complementary surface tethered thiol‐end sequence for detection. The black traces correspond to the experimental SWV signals for **dA^Fc^**/**dC^FcPa^** ratios equal to: 8:2 (A), 2:8 (B) and 4:4 (C). Red and blue traces correspond to the deconvoluted signals of **dA^Fc^** and **dC^FcPa^**, respectively. SWVs were recorded in 0.1 m Ca(NO_3_)_2_ vs. Ag/AgCl reference electrode.

The square‐wave voltammograms (Figure 4) of the three individual labelled PEX products show two clearly distinguishable peak maxima corresponding to the oxidation of **Fc** (0.3 V vs. Ag/AgCl) and **FcPa** (0.4 V vs. Ag/AgCl), respectively. After peak deconvolution, the ratios of the intensities of the two oxidation peaks correlated very well with the expected A/C nucleobase ratio present in each PEX product: 3.6±0.5 (**DNA 8A^Fc^** **2C^FcPa^**), 0.27±0.02 (**DNA 2A^Fc^** **8C^FcPa^**) and 1.04±0.15 (**DNA 4A^Fc^** **4C^FcPa^**).

## Conclusions

We have designed and synthesized nucleosides and dNTPs bearing three differently substituted ferrocene labels. Substitution with eight electron‐donating methyl groups led to a significant decrease of the oxidation potential of ferrocene (by 300 mV), whilst the attachment of the electron‐withdrawing carboxamide shifted the redox potential to 100 mV higher values. The octamethyferrocene‐linked nucleosides and nucleotides were insufficiently stable due to their spontaneous oxidation in air. However, the unsubstituted ferrocene (Fc) and ferrocenecarboxamide (FcPa) labels were stable and useful for electrochemical labelling. The corresponding redox‐labelled **dN^FcX^TP**s were good substrates for KOD XL DNA polymerase and were efficiently used for the enzymatic synthesis of DNA probes containing either one or even two redox‐labelled nucleotides using PEX. The oxidation peaks associated with **dA^Fc^** and **dC^FcPa^** were clearly distinguishable and ratiometric. Thus, for the first time, we describe a set of two fully orthogonal and ratiometric oxidizable labels for DNA suitable for the redox‐coding of nucleobases. The PEX using tailed templates and hybridization with capture probes on gold electrodes is a very efficient and straightforward strategy to study the incorporation of the two redox labels to DNA and thus directly measure the relative abundance of A and C in an unknown target sequence of DNA. In the future, the combination of these two ferrocene‐based oxidizable labels (Fc, FcPa) with some of the previously reported reducible labels (e.g. benzofurazane, nitrophenyl, azidophenyl)^5^, ^6^, ^7^, ^8^ will be tested for completing of the full set of orthogonal labels for redox coding and detection of all four nucleobases.

## Experimental Section

Complete experimental procedures and methods, characterization of all compounds, additional figures and copies of spectra are given in the Supporting Information.

## Conflict of interest

The authors declare no conflict of interest.

## Supporting information

As a service to our authors and readers, this journal provides supporting information supplied by the authors. Such materials are peer reviewed and may be re‐organized for online delivery, but are not copy‐edited or typeset. Technical support issues arising from supporting information (other than missing files) should be addressed to the authors.

Supplementary
