# Supplementary material for: Tuning of Oxidation Potential of Ferrocene for Ratiometric Redox Labeling and Coding of Nucleotides and DNA
Source: Chemistry. 2020 Jan 9;26(6):1286–91. doi: 10.1002/chem.201904700 (PMC7384099; doi:10.1002/chem.201904700)
Supplement: Supplementary file 1 — Supplementary [file CHEM-26-1286-s001.pdf]

CHEMISTRY
A European Journal

Supporting Information

Tuning of Oxidation Potential of Ferrocene for Ratiometric Redox Labeling and Coding of Nucleotides and DNA

Anna Simonova,[a, b] Ivan Magriñá,[c] Veronika Sýkorová,[a] Radek Pohl,[a] Mayreli Ortiz,[c] Luděk Havran,[d] Miroslav Fojta,*[d, e] Ciara K. O'Sullivan,*[c, f] and Michal Hocek*[a, b]

chem_201904700_sm_miscellaneous_information.pdf

Supporting information

Contents

1. Additional results and schemes S3

2. Experimental section – organic chemistry S4

3. Experimental section - biochemistry S13

4. Experimental section - electrochemistry S26

5. Copies of MALDI-TOF mass spectra S32

6. Copies of NMR spectra S38

7. References S48

1. Additional results and schemes

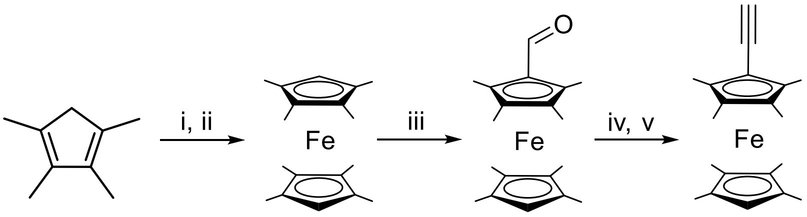

Scheme S1. Synthesis of 1-ethynyl-1',2,2',3,3',4,4',5-octamethylferrocene. Reagents and conditions: i) tetramethylcyclopentadiene in benzene, CH_3Li in E_2O, 25 °C, reflux for 4 h, ii) $FeCl_2$ (0.7 equiv.), THF, 0 °C to 25 °C, 12 h (48 %); iii) $POCl_3$ (7.8 equiv.), DMF, 60 °C, 6 h (79 %); iv) $[Ph_3PCH_2Cl]Cl$ (1.0 equiv.), THF, n-BuLi (2.5 equiv.), 25 °C, 3 h, v) t-BuOK (2.0 equiv.), reflux for 1 day (56 %).

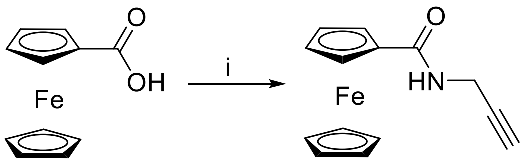

Scheme S2. Synthesis of ferrocenoyl propargylamide. Reagents and conditions: i) DCC (1.5 equiv.), DMAP (0.1 equiv.), propargylamine (1.0 equiv.), CH_2Cl_2, 25 °C, 24 h (25 %).

2. Experimental section – organic chemistry

General remarks

NMR spectra were recorded on 400 (400.0 MHz for ^{1}H, 162 MHz for ^{31}P, 100 MHz for ^{13}C), 500 (500 MHz for ^{1}H, 125.7 MHz for ^{13}C, 470.4 MHz for ^{19}F and 202.3 for ^{31}P) or 600 (600.1 MHz for ^{1}H, 150.9 MHz for ^{13}C) spectrometers from sample solutions in D_2O, DMSO-*d*$_6$, CD_3CN or CD_3OD. Chemical shifts (in ppm, δ scale) were referenced as follows: D_2O (referenced to dioxane as internal standard in 1 mm coaxial capillary; 3.75 ppm for ^{1}H NMR and 69.3 ppm for ^{13}C NMR); CD_3OD (referenced to solvent signal: 3.31 ppm for ^{1}H NMR and 49.00 ppm for ^{13}C NMR); DMSO-*d6* (referenced to solvent signal: 2.50 ppm for ^{1}H NMR and 39.7 ppm for ^{13}C NMR); CD_3CN (referenced to solvent signal: 1.94 ppm for ^{1}H NMR and 1.32 ppm for ^{13}C NMR). ^{31}P chemical shifts were referenced to H_3PO_4 as external reference. Chemical shifts are given in ppm (δ scale), coupling constants (*J*) in Hz. Complete assignment of all NMR signals was achieved by using a combination of H,H-COSY, H,C-HSQC, and H,C-HMBC experiments. Mass spectra were measured on LCQ classic (Thermo-Finnigan) spectrometer using ESI or Q-Tof Micro (Waters, ESI source, internal calibration with lockspray). Preparative HPLC separations were performed on a column packed with 10 µm C18 reversed phase (Phenomenex, Luna C18(2)). High resolution mass spectra were measured on a LTQ Orbitrap XL (Hermo Fischer Scientific) spectrometer using ESI ionization technique. Mass spectra of functionalised DNA were measured by Maldi-TOF, Reflex IV (Bruker) with nitrogen laser.

Synthesis of ferrocene labels

1,1',2,2',3,3',4,4'-octamethylferrocene

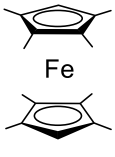

Yield: 48 %. Spectral data were in accordance with literature.[1a]

1-formyl-1',2,2',3,3',4,4',5-octamethylferrocene

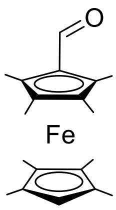

Yield: 79 %. Spectral data were in accordance with literature.[1b]

1-ethynyl-1',2,2',3,3',4,4',5-octamethylferrocene (FcMe)

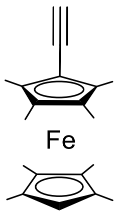

Yield: 56 %. Spectral data were in accordance with literature.[1c]

Ferrocenoyl propargylamide (FcPa)

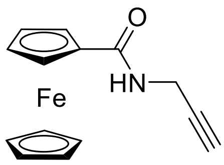

Yield: 25 %. Spectral data were in accordance with literature.[2]

Synthesis of ferrocene-labeled nucleosides

Synthesis of modified nucleosides - Sonogashira cross-coupling:

Method A: A 1:1 mixture of H_2O-CH_3CN (2 mL) was added through a septum to an argon-purged flask containing a halogenated nucleoside **dN^I^** (1 equiv.), **FcX** (1.5 equiv.), CuI (10 mol %), TPPTS (2 mol %) and $Pd(OAc)_2$ (5 mol %) followed by Et_3N (10 equiv.). The reaction mixture was stirred at 50 ºC for 40 min until complete consumption of the starting material and then evaporated under vacuum. The products were purified by silica gel column chromatography using chloroform/methanol (0 to 10 %) as eluent.

Method B: Dry DMF (3 mL) was added to an argon-purged flask containing **FcX** (1.5 equiv.), a nucleoside analogue **dN^I^** (1 equiv.), CuI (10 mol %), PPh_3 (2 mol %) and $[Pd(PPh_3)_2Cl_2]$ (5 mol %) followed by Et_3N (10 equiv.). The reaction mixture was stirred at 75 °C for 1 h until complete consumption of the starting material and then evaporated in vacuo. The products were purified by silica gel column chromatography using chloroform/methanol (0 to 10 %) as eluent.

7-(1',2,2',3,3',4,4',5-Octamethylferrocene-1-yl-ethynyl)-7-deaza-2'-deoxyadenosine (dA^{FcMe})

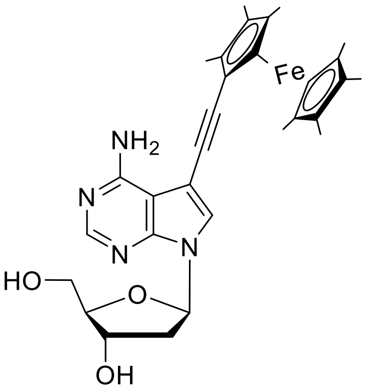

Compound **dA^{FcMe}** was prepared from **dA^{I}** according to Method A. The product was isolated as a yellow solid (45 mg, 90 %); ^{1}H NMR (500.0 MHz, CD_3OD): 1.38, 1.41, 1.53, 1.56 (4 × bs, 24H, CH_3-ferrocene); 2.32 (ddd, 1H, J_{gem} = 13.6, $J_{2'b,1'}$ = 6.0, $J_{2'b,3'}$ = 2.6 H-2′b); 2.66 (ddd, 1H, J_{gem} = 13.6, $J_{2'a,1'}$ = 8.3, $J_{2'a,3'}$ = 5.9, H-2′a); 3.58 (bs, 1H, H-ferrocene); 3.73 (dd, 1H, J_{gem} = 12.1, $J_{5'b,4'}$ = 3.5, H-5′b); 3.81 (dd, 1H, J_{gem} = 12.1, $J_{5'a,4'}$ = 3.2, H-5′a); 4.00 (ddd, 1H, $J_{4',5'}$ = 3.5, 3.2, $J_{4',3'}$ = 2.6, H-4′); 4.52 (dt, H, $J_{3',2'}$ = 5.9, 2.6, $J_{3',4'}$ = 2.6, H-3′); 6.49 (dd, 1H, $J_{1',2'}$ = 8.3, 6.0, H-1′); 7.56 (s, 1H, H-6); 8.10 (s, 1H, H-2).

^{13}C NMR (125.7 MHz, CD_3OD): 9.15, 9.97, 10.89, 11.02 (CH_3-ferrocene); 41.56 (CH_2-2′); 63.61 (CH_2-5′); 73.00 (CH-3′); 74.28 (CH-ferrocene); 82.03 (C5-**C**≡C-ferrocene); 83.85, 84.13 (C-ferrocene); 86.69 (CH-1′); 89.19 (CH-4′); 92.58 (C5-C≡**C**-ferrocene); 97.87 (C-5); 104.43 (C-4a); 126.38 (CH-6); 149.81 (C-7a); 153.22 (CH-2); 159.39 (C-4).

MS (ESI-): *m/z* (%): 571.2 (100) [M]; HRMS (ESI-): calcd. 571.2366 for $C_{31}H_{39}O_3N_4Fe$, found 571.2362.

5-(1',2,2',3,3',4,4',5-Octamethylferrocene-1-yl-ethynyl)-2'-deoxycytidine (dC^{FcMe})

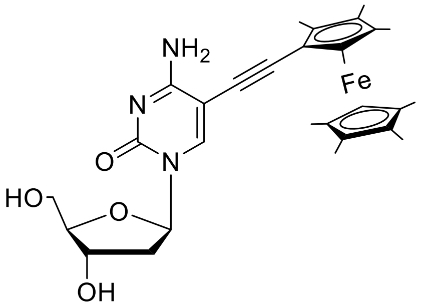

Compound **dC^{FcMe}** was prepared from **dC^{I}** according to Method A. The product was isolated as a yellow solid (40 mg, 86 %); ^{1}H NMR (500.0 MHz, CD_3OD): 0.35 – 1.24 (bm, 24H, CH_3-ferrocene); 2.10 (dt, 1H, J_{gem} = 13.6, $J_{2'b,1'}$ = $J_{2'b,3'}$ = 6.2, H-2′b); 2.37 (ddd, 1H, J_{gem} = 13.6, $J_{2'a,1'}$ = 6.2, $J_{2'a,3'}$ = 4.0, H-2′a); 3.68 (dd, 1H, J_{gem} = 12.1, $J_{5'b,4'}$ = 3.5, H-5′b); 3.74 (dd, 1H, J_{gem} = 12.1, $J_{5'a,4'}$ = 3.2, H-5′a); 3.92 (ddd, 1H, $J_{4',3'}$ = 4.0, $J_{4',5'}$ = 3.5, 3.2, H-4′); 4.31 (dt, 1H, $J_{3',2'}$ = 6.2, 4.0, $J_{3',4'}$ = 4.0, H-3′); 6.19 (t, 1H, $J_{1',2'}$ = 6.2, H-1′); 8.31 (bs, 1H, H-6); (CH-ferrocene not detected).

^{13}C NMR (125.7 MHz, CD_3OD): 8.77, 9.41, 10.28, 10.59 (CH_3-ferrocene); 42.39 (CH_2-2′); 62.39 (CH_2-5′); 71.77 (CH-3′); 76.70 (C5-**C**≡C-ferrocene); 87.96 (CH-1′); 89.12 (CH-4′); 94.68 (C-5); 97.24 (C5-C≡**C**-ferrocene); 143.46 (CH-6); 156.57 (C-2); 165.49 (C-4); (C,CH-ferrocene not detected)

MS (ESI-): *m/z* (%): 547.2 (100) [M]; HRMS (ESI-): calcd. 547.2328 for $C_{29}H_{37}O_4N_3Fe$, found 547.2131.

7-[3-(Ferrocene-1-carboxamido)prop-1-yn-1-yl]-7-deaza-2'-deoxyadenosine (**dA^{FcPa}**)

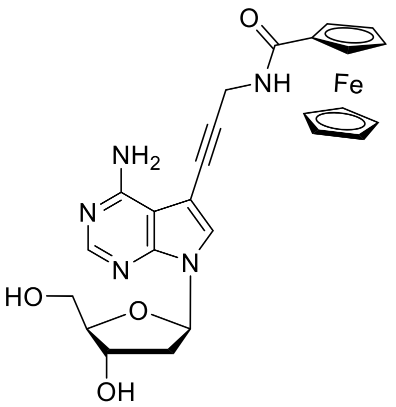

Compound **dA^{FcPa}** was prepared from **dA^{I}** according to Method B. The product was isolated as a yellow solid (52 mg, 98 %); ^{1}H NMR (500.0 MHz, CD_3OD): 2.31 (ddd, 1H, J_{gem} = 13.7, $J_{2'b,1'}$ = 6.0, $J_{2'b,3'}$ = 2.7 H-2′b); 2.61 (ddd, 1H, J_{gem} = 13.7, $J_{2'a,1'}$ = 8.0, $J_{2'a,3'}$ = 6.0, H-2′a); 3.70 (dd, 1H, J_{gem} = 12.1, $J_{5'b,4'}$ = 3.7,H-5′b); 3.77 (dd, 1H, J_{gem} = 12.1, $J_{5'a,4'}$ = 3.3,H-5′a); 3.99 (ddd, 1H, $J_{4',5'}$ = 3.7, 3.3, $J_{4',3'}$ = 2.7, H-4′); 4.21 (bs, 5H, H-cp); 4.25 (bs, 2H, CH_2N); 4.37 – 4.45 (m, 2H, H-2″,5″); 4.49 (dt, H, $J_{3',2'}$ = 6.0, 2.7, $J_{3',4'}$ = 2.7, H-3′); 4.83 – 4.85 (m, 2H, H-3″,4″); 6.46 (dd, 1H, $J_{1',2'}$ = 8.0, 6.0, H-1′); 7.58 (s, 1H, H-6); 8.15 (bs, 1H, H-2).

^{13}C NMR (125.7 MHz, CD_3OD): 30.90 (CH_2N); 41.55 (CH_2-2′); 63.61 (CH_2-5′); 69.52, 69.54 (CH-3″,4″); 70.82 (CH-cp); 72.04 (CH-2″,5″); 72.96 (CH-3′); 75.74, 75.86 (C-1″, C5-**C**≡C-CH_2); 86.63 (CH-1′); 89.18 (CH-4′); 90.43 (C5-C≡**C**-CH_2); 97.16 (C-5); 104.77 (C-4a); 127.78 (CH-6); 149.83 (C-7a); 153.49 (CH-2); 159.00 (C-4); 174.14 (CONH).

MS (ESI-): *m/z* (%): 516.1 (90) [M]; 538.1 (100) [M+Na]; HRMS (ESI-): calcd. 516.1329 for $C_{25}H_{26}O_4N_5Fe$, found 516.1327; calcd. 538.1148 for $C_{25}H_{25}O_4N_5FeNa$, found 538.1148.

5-[3-(Ferrocene-1-carboxamido)prop-1-yn-1-yl]-2'-deoxycytidine (**dC^{FcPa}**)

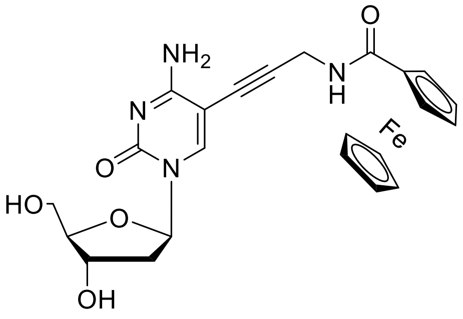

Compound **dC^{FcPa}** was prepared from **dC^{I}** according to Method B. The product was isolated as a yellow solid (48 mg, 97 %); ^{1}H NMR (600.1 MHz, DMSO-d_6): 1.95 (ddd, 1H, J_{gem} = 13.2, $J_{2'b,1'}$ = 7.2, $J_{2'b,3'}$ = 6.0, H-2'b); 2.12 (ddd, 1H, J_{gem} = 13.2, $J_{2'a,1'}$ = 5.9, $J_{2'a,3'}$ = 3.3, H-2'a); 3.52, 3.57 (2 × ddd, 2 × 1H, J_{gem} = 11.9, $J_{5',OH}$ = 5.2, $J_{5',4'}$ = 3.5, H-5'); 3.77 (q, 1H, $J_{4',3'}$ = $J_{4',5'}$ = 3.5, H-4'); 4.18 (m, 8H, H-3', CH_2N, H-cp); 4.38 (m, 2H, H-2",5"); 4.81 (m, 2H, H-3",4"); 5.07 (bt, 1H, $J_{OH,5'}$ = 5.2, OH-5'); 5.21 (bd, 1H, $J_{OH,3'}$ = 4.2, OH-3'); 6.09 (dd, 1H, $J_{1',2'}$ = 7.2, 5.9, H-1'); 6.84, 7.88 (2 × bs, 2 × 1H, NH_2); 8.12 (s, 1H, H-6); 8.29 (t, 1H, *J* = 5.4, NH).

^{13}C NMR (150.9 MHz, DMSO-d_6): 29.68 (CH_2N); 40.98 (CH_2-2'); 61.29 (CH_2-5'); 68.47 (CH-3",4"); 69.57 (CH-cp); 70.45 (CH-3', CH-2",5"); 74.23 (C5-**C**≡C-CH_2); 75.83 (C-1"); 85.56 (CH-1'); 87.68 (CH-4'); 89.71 (C-5); 93.58 (C5-C≡**C**-CH_2); 143.72 (CH-6); 153.67 (C-2); 164.69 (C-4); 169.67 (CONH).

MS (ESI-): *m/z* (%): 493.1 (10) [M]; 515.1 (100) [M+Na]; HRMS (ESI-): calcd. 493.1169 for $C_{23}H_{25}O_5N_4Fe$, found 493.1164; calcd. 515.0988 for $C_{23}H_{24}O_5N_4FeNa$, found 515.0983.

Synthesis of ferrocene-labeled dNTPs

Synthesis of modified nucleoside triphosphates - Sonogashira cross-coupling

Method A: A 1:1 mixture of H_2O-CH_3CN (2 mL) was added through a septum to an argon-purged flask containing a halogenated nucleotide **dN^{I}TP** (1 equiv.), **FcX** (1.5 equiv.), CuI (10 mol %), Et_3N (10 equiv.), PPh_3 (2 mol %) and [Pd(PPh_3)$_2$$Cl_2$] (5 mol %). The flask was evacuated and purged with argon, and then the reaction mixture was stirred at 60 ºC for 1 h until complete consumption of the starting material and then evaporated under vacuum. Product was isolated from the crude reaction mixture by HPLC on a C18 column with the use of a linear gradient of 0.1 M TEAB (triethylammonium bicarbonate) in H_2O to 0.1 M TEAB in H_2O–MeOH (1:1) as eluent. Several co-distillations with water followed by freeze-drying from water gave a solid product.

Synthesis of modified nucleoside triphosphates - triphosphorylation

Method B: $POCl_3$ (2.5 equiv) in PO(OMe)$_3$ (1 ml) was added through a septum to an argon-purged flask containing modified nucleosides **dN^{EPT}** (1 equiv.), Reaction mixture was then stirred at 0 ºC for 12 h until complete consumption of the starting material. Then an ice-cooled solution of $(NHBu_3)_2H_2P_2O_7$ (5 equiv) and Bu_3N (4.2 equiv) in dry DMF (2 ml) was added and the mixture was stirred at 0 °C for another 1.5 h. The reaction was quenched by addition of 2 M aqueous TEAB (triethylammonium bicarbonate) (2 ml) and the solvents were evaporated under vacuum and the residue was co-distilled with water three times. The product was isolated by HPLC on a C18 column with the use of a linear gradient of 0.1 M TEAB in H_2O to 0.1 M TEAB in H_2O–MeOH (1:1) as eluent. Several co-distillations with water followed by freeze-drying from water gave solid product.

7-(1',2,2',3,3',4,4',5-Octamethylferrocene-1-yl-ethynyl)-7-deaza-2'-deoxyadenosine 5'-*O*-triphosphate (**dA^{FcMe}TP**)

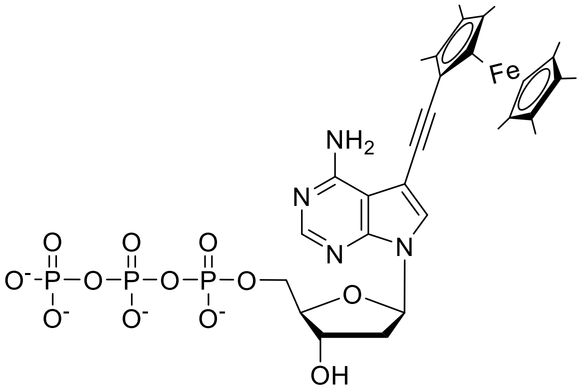

Compound **dA^{FcMe}TP** was prepared from **dA^{I}TP** by aqueous Sonogashira cross-coupling (Method A, 20 mg, 38 %) and from **dA^{FcMe}** according to the general triphosphorylation procedure (Method B, 8 mg, 15 %). The product was isolated as a yellow solid. ^{1}H and ^{13}C NMR spectra were not possible to analyze because of partial oxidation of ferrocene moiety.

^{31}P{^{1}H} NMR (162 MHz, D_2O): -20.41 (t, J = 19,6 P_{β}); -10.29 (d, J = 19.6, P_{α}); -4.10 (d, J = 20.2, P_{γ}).

MS (ESI-): *m/z* (%): 729.2 (100) [M-H_2PO_3]; 649.2 (60) [M-$H_3P_2O_6$]; HRMS (ESI-): calcd 808.1132 for $C_{31}H_{39}O_{12}N_4FeP_3$, found 808.1119.

5-(1',2,2',3,3',4,4',5-Octamethylferrocene-1-yl-ethynyl)-2'-deoxycytidine 5'-*O*-triphosphate (**dC^{FcMe}TP**)

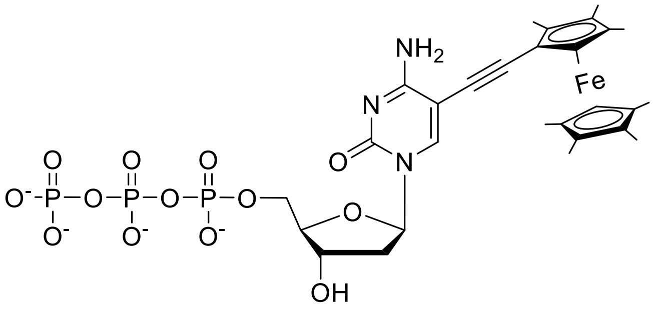

Compound **dC^{FcMe}TP** was prepared from **dC^{I}TP** by aqueous Sonogashira cross-coupling (Method A, 18 mg, 30 %) and from **dC^{FcMe}** according to the general triphosphorylation procedure (Method B, 7 mg, 20 %). The product was isolated as a yellow solid. ^{1}H and ^{13}C NMR spectra were not possible to analyse because of the partial oxidation of the ferrocene moiety.

^{31}P{^{1}H} NMR (162 MHz, D_2O): -19.62 (t, J = 20.5, P_{β}); -9.40 (d, J = 20.1, P_{α}); -3.48 (d, J = 20.5, P_{γ}).

MS (ESI-): *m/z* (%): 706.1 (100) [M-H_2PO_3]; 626.2 (50) [M-$H_3P_2O_6$]; HRMS (ESI-): calcd. 785.0972 for $C_{29}H_{38}O_{13}N_3FeP_3$, found 785.0964.

7-[3-(Ferrocene-1-carboxamido)prop-1-yn-1-yl]-7-deaza-2'-deoxyadenosine 5'-*O*-triphosphate (dA^{FcPa}TP)

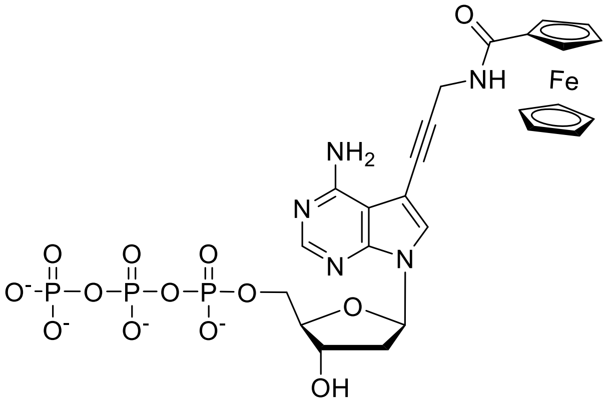

Compound **dA^{FcPa}TP** was prepared from **dA^{I}TP** by aqueous Sonogashira cross-coupling (Method A, 8 mg, 13 %) and from **dA^{FcPa}** according to the general triphosphorylation procedure (Method B, 25 mg, 22 %). The product was isolated as a yellow solid. ^{1}H NMR (500.0 MHz, D_2O): 1.20 (bm, 36H, C**H**$_3$CH$_2$N); 2.43 (ddd, 1H, J_{gem} = 13.7, $J_{2'b,1'}$ = 6.0, $J_{2'b,3'}$ = 3.4 H-2′b); 2.61 (ddd, 1H, J_{gem} = 13.7, $J_{2'a,1'}$ = 7.8, $J_{2'a,3'}$ = 6.0, H-2′a); 3.11 (bm, 24H, CH$_3$C**H$_2$**N); 4.04 – 4.13 (m, 2H, H-5′); 4.17 (q, 1H, $J_{4',3'}$ = $J_{4',5'}$ = 3.4, H-4′); 4.22 (bs, 5H, H-cp); 4.26 (bs, 2H, CH$_2$N); 4.46 (m, 2H, H-2″,5″); 4.68 (dt, H, $J_{3',2'}$ = 6.0, 3.4, $J_{3',4'}$ = 3.4, H-3′); 4.79 (bs, 2H, H-3″,4″); 6.46 (dd, 1H, $J_{1',2'}$ = 7.8, 6.0, H-1′); 7.60 (s, 1H, H-6); 8.03 (bs, 1H, H-2).

^{13}C NMR (125.7 MHz, D_2O): 10.94 (**CH**$_3$CH$_2$N); 32.68 (CH$_2$N); 41.15 (CH$_2$-2′); 42.97 (CH$_3$**C**H$_2$N); 68.28 (d, $J_{C,P}$ = 4.9, CH$_2$-5′); 71.19, 71.21 (CH-3″,4″); 72.74 (CH-cp); 73.69 (CH-3′); 74.50 (CH-2″,5″); 75.88 (C-1″); 7.47 (C5-**C**≡C-CH$_2$); 85.50 (CH-1′); 87.96 (d, $J_{C,P}$ = 8.7, CH-4′); 92.15 (C5-C≡**C**-CH$_2$); 99.01 (C-5); 105.56 (C-4a); 128.80 (CH-6); 151.18 (C-7a); 154.90 (CH-2); 159.84 (C-4); 176.97 (CONH).

^{31}P NMR (202.3 MHz, D_2O): -22.36 (bt, J = 19.4, P_β); -10.75 (d, J = 19.4, P_α); - 7.87 (bd, J = 19.4, P_γ).

MS (ESI-): 754.0 [M-H]; 674.0 [M-H$_2$PO$_3$]; HRMS (ESI-): calcd. 754.0173 for $C_{25}H_{27}O_{13}N_5FeP_3$, found 754.0169.

5-[3-(Ferrocene-1-carboxamido)prop-1-yn-1-yl]-2'-deoxycytidine 5'-*O*-triphosphate (dC^{FcPa}TP)

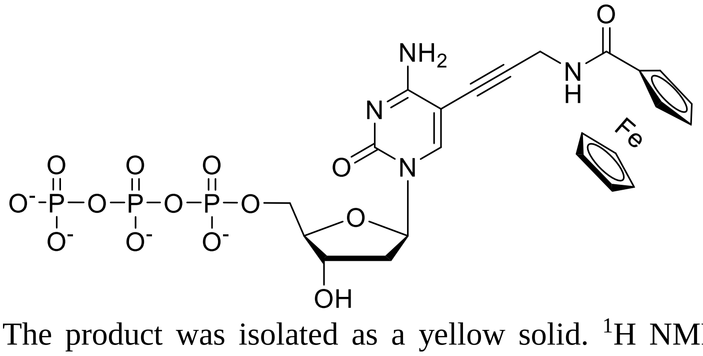

Compound **dC^{FcPa}TP** was prepared from **dC^{I}TP** by aqueous Sonogashira cross-coupling (Method A, 10 mg, 16 %) and from **dC^{FcPa}** according to the general triphosphorylation procedure (Method B, 20 mg, 18 %). The product was isolated as a yellow solid. ^{1}H NMR (500.0 MHz, D_2O): 1.26 (bm, 36H, C**H**$_3$CH$_2$N); 2.26 (dt, 1H, J_{gem} = 13.7, $J_{2'b,1'}$ = 6.8, $J_{2'b,3'}$ = 6.4, H-2′b); 2.40 (ddd, 1H, J_{gem} =

13.7, $J_{2'a,1'}$ = 6.1, $J_{2'a,3'}$ = 4.7, H-2′a); 3.17 (bm, 24H, $CH_3C\mathbf{H_2}N$); 4.17 (m, 1H, H-4′); 4.18 – 4.26 (m, 2H, H-5′); 4.26 – 4.33 (m, 7H, CH_2N, H-cp); 4.54 (m, 2H, H-2″,5″); 4.58 (dt, H, $J_{3',2'}$ = 6.4, 4.7, $J_{3',4'}$ = 4.7, H-3′); 4.85 (m, 2H, H-3″,4″); 6.22 (dd, 1H, $J_{1',2'}$ = 6.8, 6.1, H-1′); 8.18 (s, 1H, H-6).

^{13}C NMR (125.7 MHz, D_2O): 10.98 ($\mathbf{C}H_3CH_2N$); 32.52 (CH_2N); 42.02 (CH_2-2′); 49.33 ($CH_3\mathbf{C}H_2N$); 67.75 (d, $J_{C,P}$ = 4.6, CH_2-5′); 71.30, 71.34 (CH-3″,4″); 72.69 (CH-3′); 72.84 (CH-cp); 74.64, 74.65 (CH-2″,5″); 75.73 (C1″, C5-**C**≡C-CH_2); 88.21 (d, $J_{C,P}$ = 8.6, CH-4′); 88.82 (CH-1′); 95.05 (C-5); 95.60 (C5-C≡**C**-CH_2); 147.67 (CH-6); 158.68 (C-2); 167.83 (C-4); 177.10 (CONH).

^{31}P NMR (202.3 MHz, D_2O): -22.42 (t, J = 20.0, P_β); -10.83 (d, J = 20.0, P_α); -8.33 (bd, J = 20.0, P_γ).

MS (ESI-): 731.0 [M-H]; 651.0 [M-H_2PO_3]; HRMS (ESI-): calcd. 731.0013 for $C_{23}H_{26}O_{14}N_4FeP_3$, found 731.0006.

5-(Ferrocene-1-yl-ethynyl)-2'-deoxycytidine 5'-*O*-triphosphate (**dC^{Fc}TP**)

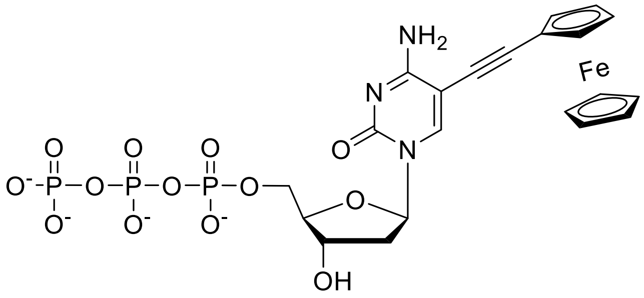

Compound **dC^{Fc}TP** was prepared in analogy to the literature [7] by the Sonogashira coupling of **dC^{I}TP** (0.05 mmol) with ethynylferrocene (16 mg, 0.078 mmol) in presence of $Pd(OAc)_2$ (0.6 mg, 5 mol %), CuI (1 mg, 10 mol %) and TPPTS (tris(3-sulfonatophenyl)phosphine) (7.5 mg, 25 mol %) in H_2O/acetonitrile (2:1, 1 ml) containing Et_3N (58 μL, 8 equiv.). The mixture was stirred under argon at 70 °C for 1 h, evaporated and the product isolated by reverse phase HPLC on C18 column with a linear gradient of 0.1 M TEAB (triethylammonium bicarbonate) in H_2O to 0.1 M TEAB in H_2O/MeOH (1:1), followed by repeated coevaporation with water and conversion to sodium salt using Dowex 50WX8 in Na^+ cycle. The product was obtained as a trisodium salt by freeze-drying from water in 27 % yield. ^{1}H NMR (600.1 MHz, D_2O): 2.34, 2.45 (2 × m, 2 × 1H, H-2'); 4.17-4.28 (m, 3H, H-4',5'); 4.34 (bs, 5H, cp); 4.40 (bs, 2H, H-3",4"); 4.60-4.68 (bm, 3H, H-3' and H-2",5") 6.48 (bt, 1H, $J_{1',2'}$ = 5.9, H-1'); 7.72 (s, 1H, H-8); 8.23 (bs, 1H, H-2).

^{13}C NMR (150.9 MHz, D_2O): 41.70 (CH2-2'); 65.92 (C-1"); 67.97 (d, $J_{C,P}$ = 5.3, CH_2-5'); 72.31 (CH-3",4"); 72.89 (CH-cp); 73.02 (CH-3'); 74.28 (CH-2",5"); 78.26 (C≡C-fer); 88.20 (d, $J_{C,P}$ =

8.5, CH-4'); 89.02 (CH-1'); 96.19 (C≡C-fer); 98.22 (C-5); 146.16 (CH-6); 158.76 (C-2); 167.58 (C-4).

^{31}P{^{1}H} NMR (202.3 MHz, D_2O): -21.70 (t, *J* = 19.2, P_β); -11.08 (d, *J* = 19.2, P_α); -5.91 (d, *J* = 19.2, P_γ).

MS (ESI-): *m/z* (%): 739.9 (15) [M]; 717.9 (100) [M-Na]; 696.0 (85) [M-Na_2]; 616.0 [M-Na_2PO_3]; 594.0 [M-Na_3PO_3]; 514.0 [M-$Na_3P_2O_6$]; HRMS (ESI-): calcd. 739.92570 for $C_{21}H_{20}O_{13}N_3FeNa_3P_3$; found: 739.92571.

3. Experimental section - biochemistry

General remarks

All gels were analysed by fluorescence imaging using Typhoon FLA 9500 (GE Healthcare). Mass spectra of oligonucleotides were measured on UltrafleXtreme MALDI-TOF/TOF (Bruker) mass spectrometer with 1 kHz smartbeam II laser. UV-Vis spectra were measured at room temperature in a NanoDrop1000 (ThermoScientific). Synthetic oligonucleotides (primers labelled at 5´-end with a 6-carboxyfluorescein (6-FAM), templates and biotinylated templates; for sequences see Table S1) were purchased from Generi Biotech (Czech Republic). Natural nucleoside triphosphates (dATP, dGTP, dTTP, dCTP) were purchased from ThermoScientific. KOD XL DNA polymerase and corresponding polymerase reaction buffer from Merck (Sigma Aldrich), Pwo DNA polymerase and Terminal transferase (TdT) were purchased from New England Biolabs. Streptavidin magnetic particles (Roche) were obtained from Sigma Aldrich. Milli-Q water was used for all experiments. PAGE stop solution used after PEX reactions contains: 95 % [v/v] formamide, 0.5 mM EDTA, 0.025 % [w/v] bromophenol blue and 0.025 % [w/v] xylene cyanol, 0.025 % [w/v] SDS and Milli-Q water. Samples after PEX reactions were separated with a 12.5 % PAGE (acrylamide/bisacrylamide 19:1, 25 % urea) under denaturing conditions (TBE 1X). Other chemicals were of analytical grade. Samples after PCR reactions were separated with a 2 % agarose gel (Agarose, research grade, Serva) in 0.5X TBE buffer, stained with GelRed (10,000X in water, Biotium).

Table S1. List of sequences of primers and templates.

Name	Sequence
primer^{rnd}	5'-CATGGGCGGCATGGG-3'
primer^{LT25TH}	5‘-CAAGGACAAAATACCTGTATTCCTT-3‘
primer^{L20-}	5‘-GACATCATGAGAGACATCGC-3‘
temp^{rnd16}	5'-CTAGCATGAGCTCAGTCCCATGCCGCCCATG-3'
temp^{A}	5'-CCCTCCCATGCCGCCCATG-3'
temp^{C}	5'-CCCGCCCATGCCGCCCATG-3'
temp^{termA}	5'-TCCCATGCCGCCCATG-3'
temp^{termC}	5'-GCCCATGCCGCCCATG-3'
temp^{FVLA}	5‘-GACATCATGAGAGACATCGCCTCTGGGCTAATAGGACTACTTCT<br>AATCTGTAAGAGCAGATCCCTGGACAGGCAAGGAATACAGGTATTT<br>TGTCCTTG-3‘
temp$^{1A,1C-bio}$	5′-CCACCATCCGCC CAT GCC GCC CAT G-3′
temp$^{rnd16-bio}$	5'-CTAGCATGAGCTCAGTCCCATGCCGCCCATG-3'
temp$^{1-C3-1A,1C}$	5′-ATTACGACGAACTCAATGAA-**C3**-CCACCATCCGCCCATGCCGCCC<br>ATG - 3′
temp$^{2-C3-rnd16}$	5′- ATTACGACGAACTCAATGAA-**C3**-CTAGCATGAGCTCAGTCCCATG<br>CCGCCCATG - 3′
temp$^{3-C3-Repet4*A,C}$	5′-ATTACGACGAACTCAATGAA-**C3**-ACTGACTGACTGACTGCCCATG<br>CCGCCCATG - 3′
primer$^{8/2_4/4}$	5’-CATGGGCGGCCATTG G-3’
temp^{8A,2C}	5’-ATTACGACGAACTCAATGAA – **C3** – TCGATAGCCTACCTACAC<br>CACACCTCAATACACTACCTACCAATGGCCGCCCATG-3′
temp^{8C,2A}	5’-ATTACGACGAACTCAATGAA – **C3** – GCTAGATCCGACCGACAC<br>CACACCGCAAGACACGACCGACCAATGGCCGCCCATG-3’
temp^{4C,4A}	5’-ATTACGACGAACTCAATGAA – **C3** – GCTATAGCCTACGCAAGA<br>CCTCCAATGGCCGCCCATG-3’
Capture probe	5′- TTCATTGAGTTCGTCGTAATTTTTTTTTTTTTTTT-3′-**C6-SH**

In the template ONs the segments forming duplex with the primer are underlined.

Primer extension experiment – analysis and isolation of PEX products

Single incorporation (templates temp^{A} and temp^{C})

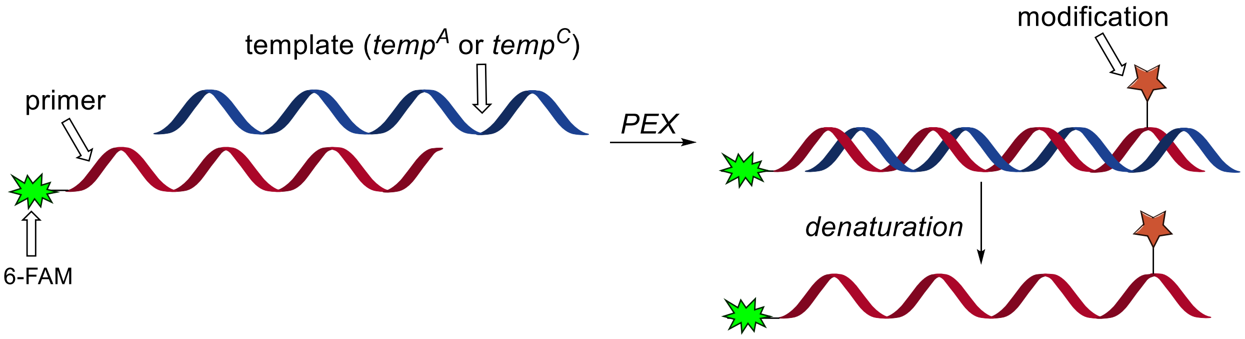

Scheme S3. Single nucleotide incorporation by PEX.

Method A: Reaction mixture (20 μL) contained temp^{A} (3 μM, 1 μl), 5′-6-(FAM)-labelled primer^{rnd} (3 μM, 1.5 μl), dGTP (4 mM, 0.1 μl), either dATP or **dA^{FcX}TP** (4 mM, 1 μl) KOD XL DNA polymerase (0.125 U) and reaction buffer (10X, 2 μL) as supplied by the manufacturer. The reaction mixture was incubated for 40 minutes at 60 °C. The PEX reaction was stopped by addition of PAGE stop solution (20 μL) and heated for 2 minutes at 95 °C. Samples were analysed with a 12.5 % denaturing polyacrylamide gel electrophoresis (1 h, 50 °C) and visualised using fluorescence imaging (Figure S1).

Method B: PEX reactions with temp^{C} were performed in the same way as described for temp^{A} except either dCTP or **dC^{FcX}TP** (4 mM, 1μl) were used (Figure S1).

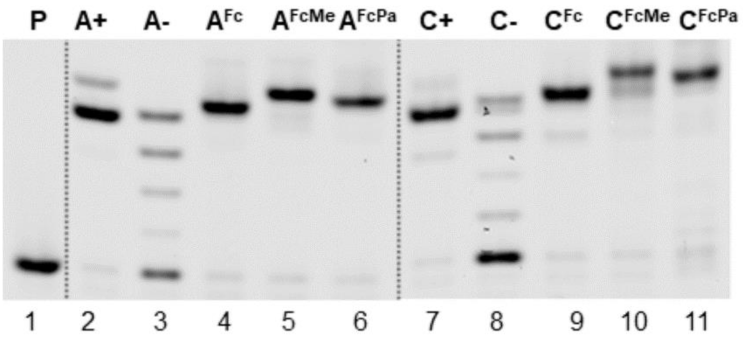

Figure S1. Primer extension with a KOD XL DNA polymerase using prim^{rnd}, temp^{A}, and temp^{C}: (P) primer (5'-6-FAM-labelled); (A+) dATP, dGTP; (A-) dGTP; (A^{Fc}) **dA^{Fc}TP**, dGTP; (A^{FcMe}) **dA^{FcMe}TP**, dGTP; (A^{FcPa}) **dA^{FcPa}TP**, dGTP; (C+) dCTP, dGTP; (C-) dGTP; (C^{Fc}) **dC^{Fc}TP**, dGTP; (C^{FcMe}) **dC^{FcMe}TP**, dGTP; (C^{FcPa}) **dC^{FcPa}TP**, dGTP.

Multiple incorporation (template temp^{rnd16})

The reaction mixture (20 µL) contained template temp^{rnd16} (3 µM, 1 µl), 5′-(FAM)-labelled primer^{rnd} (3 µM, 1.5 µl), dNTP (either natural or modified, 4 mM, 1 µl), KOD XL DNA polymerase (0.25 U) and reaction buffer (10X, 2 µL) as supplied by the manufacturer. The reaction mixture was incubated for 40 minutes at 60 °C. The PEX reaction was stopped by addition of PAGE stop solution (20 µL) and heated for 2 minutes at 95 °C. Samples were analysed with a 12.5 % denaturing polyacrylamide gel electrophoresis (1 h, 50 °C) and visualised using fluorescence imaging (Figure S2).

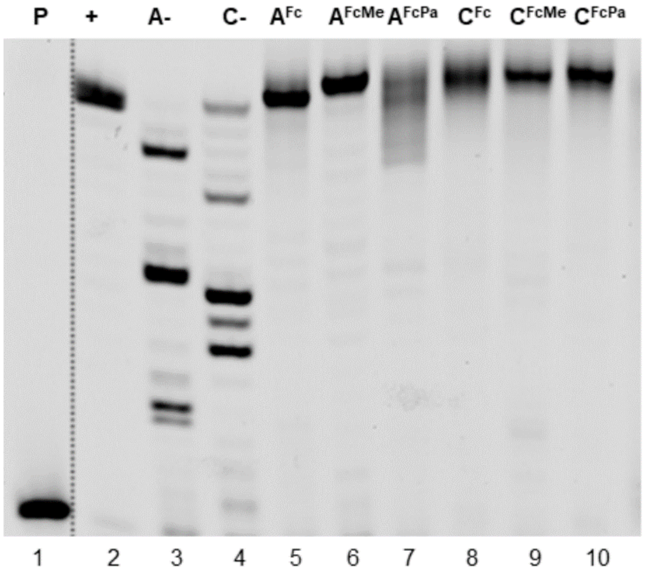

Figure S2. Primer extension with KOD XL DNA polymerase using prim^{rnd}, temp^{rnd16}: (P) primer (5'-6-FAM-labelled); (+) natural dNTPs; (A-) dCTP, dTTP, dGTP; (C-) dATP, dTTP, dGTP; (A^{Fc}) **dA^{Fc}TP**, dCTP, dTTP, dGTP; (C^{Fc}) **dC^{Fc}TP**, dATP, dTTP, dGTP; (A^{FcMe}) **dA^{FcMe}TP**, dCTP, dTTP, dGTP; (C^{FcMe}) **dC^{FcMe}TP**, dATP, dTTP, dGTP; (A^{FcPa}) **dA^{FcPa}TP**, dCTP, dTTP, dGTP; (C^{FcPa}) **dC^{FcPa}TP**, dATP, dTTP, dGTP.

Kinetics of incorporation of modified dNTPs (templates temp^{termA} and temp^{termC})

The simplified kinetics studies in the presence of KOD XL DNA polymerase were performed to examine the incorporation efficiency of modified **dN^{FcX}TP**s by PEX experiments in comparison with natural dNTPs (Figures S3-S6). The rates of the PEX with natural dNTPs or modified nucleotides were revealed using templates temp^{termA} or temp^{termC} and primer^{rnd}. The incorporation of natural dATP or dCTP were complete in 2 minutes and ferrocenene-labelled nucleotides were fully incorporated within a maximum of 1-2 minutes. The slowest extension was observed in the experiment with **dA^{FcMe}TP**.

The rate of incorporation was compared by preparation of samples with natural and modified dNTPs with various time periods. PEX reaction mixture (20 μl) was performed with 5′-6-(FAM)-labelled prim^{rnd} (3 μM, 1 μl), temp^{termA} (3 μM, 1.5 μl) or temp^{termC} (3 μM, 1.5 μl), dNTPs (4 mM, 1 μl) with KOD XL DNA polymerase (0.125 U) in the enzyme reaction buffer (10X, 2 μL) as supplied by the manufacturer, followed by stopping of the reaction using the PAGE stop solution (20 μL) and immidiately heating for 2 minutes at 95 °C. Samples were analysed with a 12.5 % denaturing polyacrylamide gel electrophoresis (1 h, 50 °C) and visualised using fluorescence imaging.

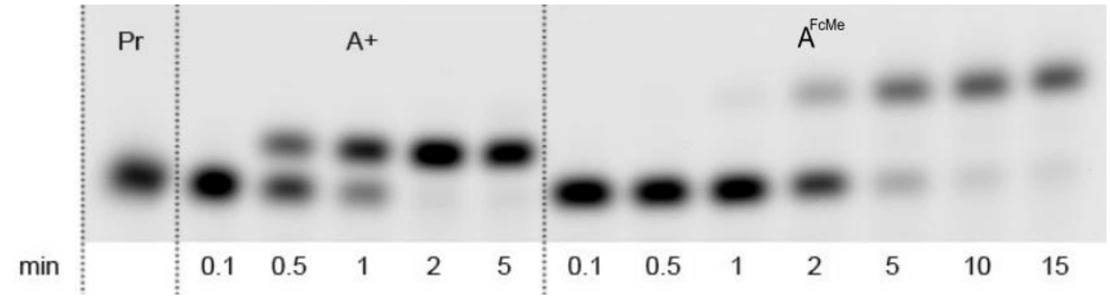

Figure S3. Comparison of incorporation of natural and modified dATPs to DNA with temp^{termA}: (Pr) primer (5'-6-FAM-labelled); (A+) dATP; (A^{FcMe}) **dA^{FcMe}TP**.

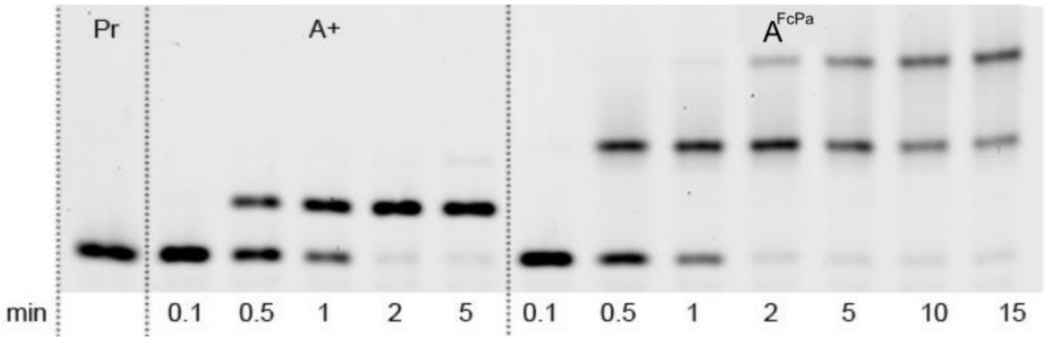

Figure S4. Comparison of incorporation of natural and modified dATPs to DNA with temp^{termA}: (Pr) primer (5'-6-FAM-labelled); (A+) dATP; (**A**^{FcPa}) **dA^{FcPa}TP**.

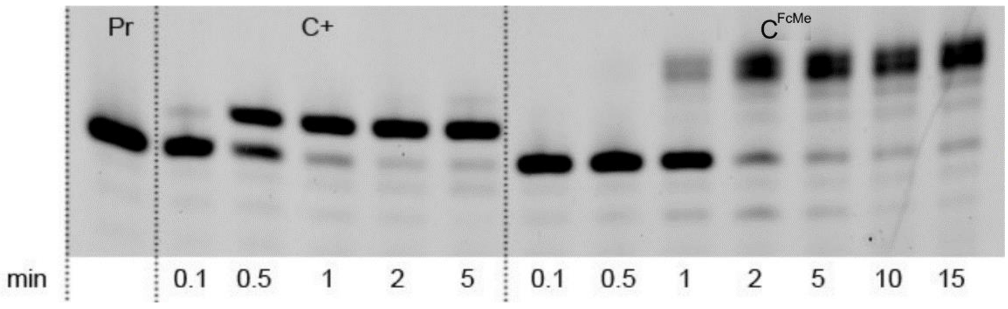

Figure S5. Comparison of incorporation of natural and modified dCTPs to DNA with temp^{termC}: (Pr) primer (5'-6-FAM-labelled); (C+) dCTP; (C^{FcMe}) **dC^{FcMe}TP**.

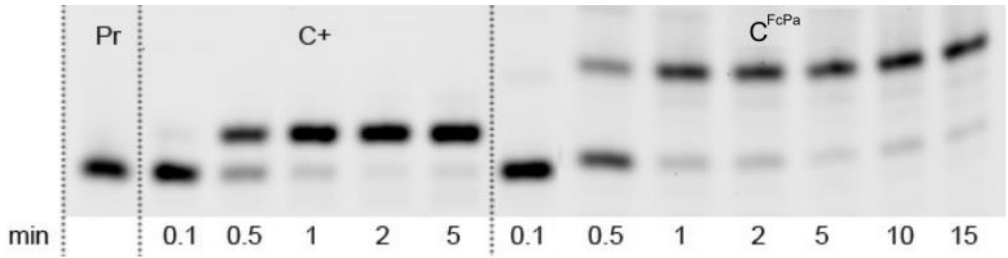

Figure S6. Comparison of incorporation of natural and modified dCTPs to DNA with temp^{termC}: (Pr) primer (5'-6-FAM-labelled); (C+) dCTP; (C^{FcPa}) **dC^{FcPa}TP**.

Isolation of single-strand oligonucleotides by the DBStv magnetoseparation procedure (templates temp^{rnd16}, temp^{A} and temp^{C})

The reaction mixture (50 µL) contained biotinylated temp^{rnd16} (temp^{A} or temp^{C}) (100 µM, 1.6 µL), primer^{rnd} (100 µM, 1.6 µL), dNTPs (4 mM, 2.6 µL), and KOD XL DNA polymerase (0.75 U) in the enzyme reaction buffer (5 µL) as supplied by the manufacturer. The reaction mixture was incubated for 1 h at 60 °C in a thermal cycler. The reaction was stopped by cooling to 4 °C. Streptavidin magnetic particles (Roche, 100 µL) were washed with Binding buffer TEN 100 (10 mM Tris, 1 mM EDTA, 100 mM NaCl, pH 7.5; 3 × 200 µL). The reaction mixture after PEX was diluted with the Binding buffer TEN 100 (50 µL), and the solution was added to the prewashed magnetic beads and incubated for 30 min at 15 °C and 1400 rpm. After the incubation, the magnetic beads were collected on a magnet (DynaMag-2, Invitrogen) and the solution was discarded. The beads were washed successively with Wash buffer TEN 500 (10 mM Tris, 1 mM EDTA, 500 mM NaCl, pH 7.5; 3 × 200 µL), and water (3 × 200 µL). Water (50 µL) was then added and the sample was denatured for 2 min at 70 °C and 1000 rpm. The beads were collected on a magnet and the solution was transferred to a clean vial. The product was analysed using MALDI-TOF mass spectrometry (Table S2).

MALDI-TOF measurements

The MALDI-TOF spectra were measured with 1 kHz smartbeam II laser technology. The measurements were carried out in reflectron and linear mode using the droplet technique, with the mass range up to 30 kDa. The matrix consisted of 3-hydroxypicolinic acid (HPA)/picolinic acid (PA)/ ammonium tartrate in ratio 9/1/1. The matrix (1 µL) was applied to the target (ground steel) and dried down at room temperature. The sample (1 µL) and matrix (1 µL) were mixed and added on the top of the dried matrix preparation spot and dried at room temperature.

Table S2. List of MALDI data of PEX products bearing modified or non-modified Fc labels.

oligonucleotide	M calcd. (Da)	M found (Da)
**31ON_4A^{Fc}**	10445.54	10447.0
**31ON_4A^{FcPa}**	10673.70	10674.1
**31ON_4C^{Fc}**	10449.54	10451,9
**31ON_4C^{FcPa}**	10677.70	10679.6
**19ON_1A^{Fc}**	6182.0	6183.2
**19ON_1A^{FcPa}**	6239.0	6238.6
**19ON_1A^{FcMe}**	6294.0	6295.4
**19ON_1C^{Fc}**	6159.0	6160.2
**19ON_1C^{FcPa}**	6216.0	3389.4
**19ON_1C^{FcMe}**	6271.0	6272.3

Single incorporation (template temp$^{1A,1C-bio}$)

The PEX reaction mixture (200 μL) contained 5′-(6-FAM)-labelled 15nt primer prim^{rnd} (3 μM, 10 μL), 25nt biotinylated template temp$^{1A,1C-bio}$ (3 μM, 15 μL), KOD XL DNA polymerase (2.5 U/μL, 0.5 μL), natural dNTPs (1 mM, 2 μL), modified dATP or dCTP (4 mM, 2.5 μL) in enzyme reaction buffer (10X, 20 μL) as supplied by the manufacturer. The reaction mixture was incubated for 10 min at 60 °C in a thermal cycler.

Small aliquots (5 μL) of the finished PEX mixture was mixed with 5 μL of PAGE stop solution and heated for 5 min at 95 °C prior to loading. Samples were separated with a 12.5 % PAGE under denaturing conditions (42 mA, 30 min). Visualisation was performed using fluorescence imaging (Figure S7).

The rest of the PEX reaction mixture was then used for the isolation of single-stranded oligonucleotides bearing one modification by magnetoseparation procedure (see below).

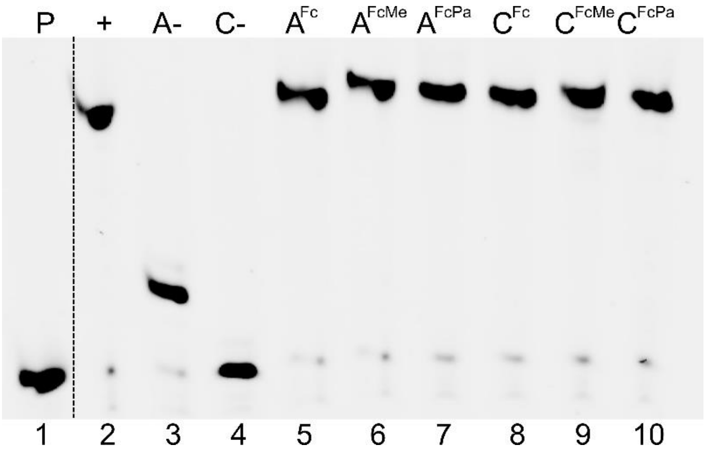

Figure S7. Primer extension with KOD XL DNA polymerase using prim^{rnd} and temp$^{1A,1C-bio}$: (P) primer (5'-6-FAM-labelled); (+) natural dNTPs; (A-) dCTP, dTTP, dGTP; (C-) dATP,

dTTP, dGTP; (A^{Fc}) **dA^{Fc}TP**, dCTP, dTTP, dGTP; (A^{FcMe}) **dA^{FcMe}TP**, dCTP, dTTP, dGTP; (A^{FcPa}) **dA^{FcPa}TP**, dCTP, dTTP, dGTP; (C^{Fc}) **dC^{Fc}TP**, dATP, dTTP, dGTP; (C^{FcMe}) **dC^{FcMe}TP**, dATP, dTTP, dGTP; (C^{FcPa}) **dC**^{FcPa}**TP**, dATP, dTTP, dGTP.

Multiple incorporation (template temp$^{rnd16\text{-}bio}$)

The PEX reaction mixture (200 μL) contained 5′-(6-FAM)-labelled 15nt primer prim^{rnd} (3 μM, 10 μL), 31nt biotinylated template temp$^{rnd16\text{-}bio}$ (3 μM, 15 μL), KOD XL DNA polymerase (2.5 U/μL, 3 μL), natural dNTPs (4 mM, 10 μL), modified dATP or dCTP (4 mM, 25 μL) in enzyme reaction buffer (10X, 20 μL) as supplied by the manufacturer. The reaction mixture was incubated for 30 min at 60 °C in a thermal cycler.

Small aliquots (5 μL) of the finished PEX mixture was mixed with 5 μL of PAGE stop solution and heated for 5 min at 95 °C prior to loading. Samples were separated with a 12.5 % PAGE under denaturing conditions (42 mA, 30 min). Visualisation was performed using fluorescence imaging (Figure S8).

The rest of the PEX reaction mixture was then used for the isolation of single-stranded oligonucleotides bearing four modifications by magnetoseparation procedure (see below).

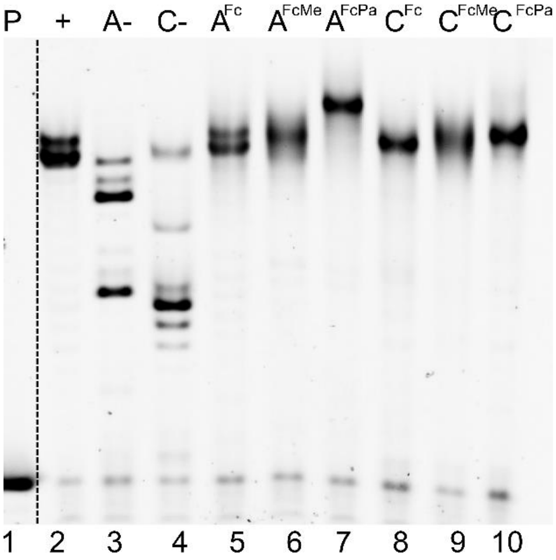

Figure S8. Primer extension with KOD XL DNA polymerase using prim^{rnd} and temp$^{rnd16\text{-}bio}$: (P) primer (5'-6-FAM-labelled); (+) natural dNTPs; (A-) dCTP, dTTP, dGTP; (C-) dATP, dTTP, dGTP; (A^{Fc}) **dA^{Fc}TP**, dCTP, dTTP, dGTP; (A^{FcMe}) **dA^{FcMe}TP**, dCTP, dTTP, dGTP; (A^{FcPa}) **dA^{FcPa}TP**, dCTP, dTTP, dGTP; (C^{Fc}) **dC^{Fc}TP**, dATP, dTTP, dGTP; (C^{FcMe}) **dC^{FcMe}TP**, dATP, dTTP, dGTP; (C^{FcPa}) **dC**^{FcPa}**TP**, dATP, dTTP, dGTP.

Magnetoseparation procedure (template temp$^{1A,1C\text{-}bio}$ and temp$^{rnd16\text{-}bio}$)

Streptavidin magnetic particles (Roche, 450 μL) were washed with Binding buffer TEN 100 (10 mM Tris, 1 mM EDTA, 100 mM NaCl, pH 7.5; 3 × 200 μL). The reaction mixture after PEX (195 μL) was mixed with the Binding buffer TEN 100 (1000 μL), and the solution was then added to the prewashed magnetic beads and incubated for 30 min at 15 °C and 1400 rpm. After the incubation, the magnetic beads were collected on a magnet (DynaMag-2, Invitrogen) and the solution was discarded. The beads were washed successively with Wash buffer TEN 500 (10 mM Tris, 1 mM EDTA, 500 mM NaCl, pH 7.5; 3 × 500 μL), and Milli-Q water (4 × 300 μL). Water (100 μL) was then added and the sample was denatured for 2 min at 72 °C and 900 rpm. The beads were collected on a magnet and the solution was transferred to a clean vial.

After magnetoseparation, small aliquots (2 μL) of all samples were mixed with 3 μL of PAGE stop solution and 10 μL of Milli-Q water and heated for 5 min at 95 °C prior to loading. Samples were separated with a 12.5 % PAGE under denaturing conditions (42 mA, 30 min). Visualisation was performed using fluorescence imaging (Figure S9).

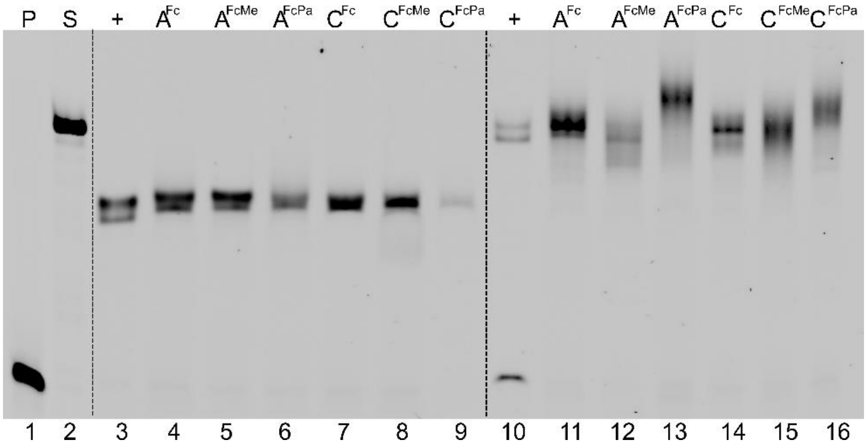

Figure S9. 12.5 % PAGE after magnetoseparation procedure. Single-stranded oligonucleotide products (Lines 3-9) with one Fc modification, 25-mer product; (Lines 10-16) with four Fc modifications, 31-mer product. (P) 15nt primer (5'-6-FAM-labelled); (S) 31nt oligonucleotide with complementar sequence to temp^{rnd16} (5'-6-FAM-labelled).

All single-stranded oligonucleotide products were lyophilized, and before use were dissolved in 10 μL of Milli-Q water.

Enzymatic synthesis of ON using tailed-templates with C3-spacer

Single incorporation (template temp$^{1\text{-}C3\text{-}1A,1C}$)

The PEX reaction mixture (20 μL) contained 5′-(6-FAM)-labelled 15nt primer prim^{rnd} (3 μM, 1 μL), template temp$^{1\text{-}C3\text{-}1A,1C}$ (3 μM, 1.5 μL), KOD XL DNA polymerase (0.125 U/μL, 1 μL), natural dNTPs (0.2 mM, 1 μL), modified dATP or dCTP (0.8 mM, 1.5 μL) in enzyme reaction buffer (10X, 2 μL) as supplied by the manufacturer. The reaction mixture was incubated for 10 min at 60 °C in a thermal cycler.

The finished PEX mixture was mixed with 20 μL of PAGE stop solution and heated for 5 min at 95 °C prior to loading. Samples were separated with a 12.5 % PAGE under denaturing conditions (42 mA, 30 min). Visualisation was performed using fluorescence imaging (Figure S10-A).

Moreover, the enzymatic synthesis was then five times scaled-up (100 μL total volume of PEX reaction mixture) in order to obtain sufficient amount of modified DNA for electrochemical experiments.

After the PEX synthesis at a higher scale, small aliquots (5 μL) of all samples were mixed with 5 μL of PAGE stop solution and heated for 5 min at 95 °C prior to loading. Samples were separated with a 12.5 % PAGE under denaturing conditions (42 mA, 30 min). Visualisation was performed using fluorescence imaging (Figure S10-B).

The remaining volume (~ 95 μL) of all samples was mixed with 20 μL of hybridization solution (H7140, Sigma Aldrich) and freezed to - 80 °C.

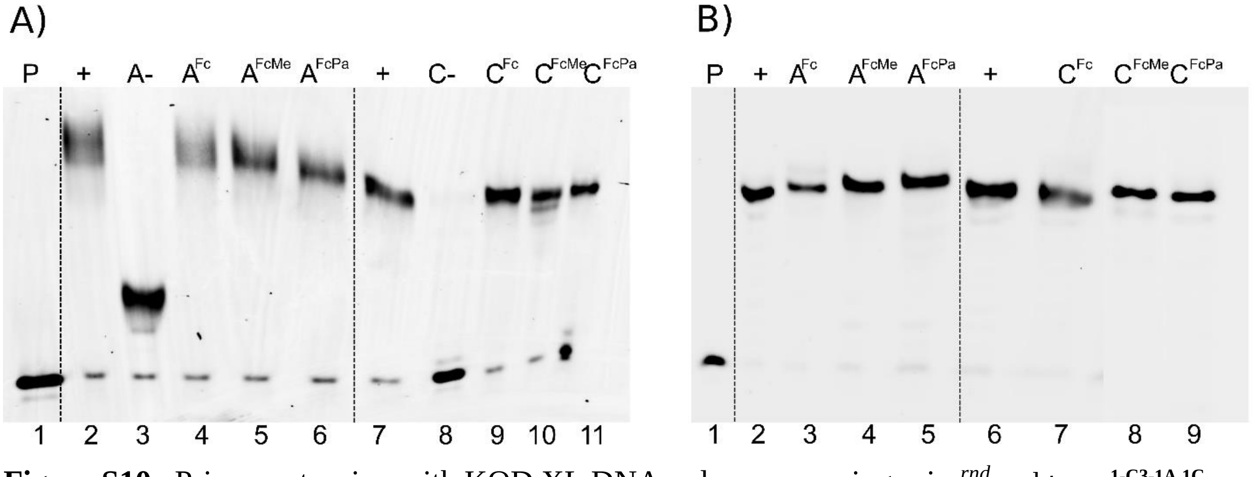

Figure S10. Primer extension with KOD XL DNA polymerase using prim^{rnd} and **temp$^{1\text{-}C3\text{-}1A,1C}$**: A) PEX in small scale (20 μL); B) PEX in larger scale (100 μL); (P) primer (5'-6-FAM-labelled); (+) natural dNTPs; (A-) dCTP, dTTP, dGTP; (C-) dATP, dTTP, dGTP; (A^{Fc}) **dA^{Fc}TP**, dCTP, dTTP, dGTP; (A^{FcMe}) **dA^{FcMe}TP**, dCTP, dTTP, dGTP; (A^{FcPa}) **dA^{FcPA}TP**,

dCTP, dTTP, dGTP; (C^{Fc}) **dC^{Fc}TP**, dATP, dTTP, dGTP; (C^{FcMe}) **dC^{FcMe}TP**, dATP, dTTP, dGTP; (C^{FcPa}) **dC^{FcPa}TP**, dATP, dTTP, dGTP.

Multiple incorporation (templates temp$^{1\text{-}C3\text{-}1A,1C}$ or temp$^{3\text{-}C3\text{-}Repet4*A,C}$)

The PEX reaction mixture (20 μL) contained 5′-(6-FAM)-labelled 15nt primer prim^{rnd} (3 μM, 1 μL), templates temp$^{1\text{-}C3\text{-}1A,1C}$ or temp$^{3\text{-}C3\text{-}Repet4*A,C}$ (3 μM, 1.5 μL), KOD XL DNA polymerase (0.75 U/μL, 1 μL),

A) in the case of **dA^{FcX}TP**: natural dNTPs (4 mM, 1 μL), modified dATP (4 mM, 2.5 μL)

B) in the case of **dC^{FcX}TP**: natural dNTPs (4 mM, 0.5 μL), modified dCTP (4 mM, 2.5 μL)

in enzyme reaction buffer (10X, 2 μL) as supplied by the manufacturer. The reaction mixture was incubated for 20 min at 60 °C in a thermal cycler.

The finished PEX mixture was mixed with 20 μL of PAGE stop solution and heated for 5 min at 95 °C prior to loading. Samples were separated with a 12.5 % PAGE under denaturing conditions (42 mA, 30 min). Visualisation was performed using fluorescence imaging (Figure S11-A, Figure S12|-A).

Moreover, the enzymatic synthesis was then five times scaled-up (100 μL total volume of PEX reaction mixture) in order to obtain a sufficient amount of modified DNA for electrochemical experiments.

After the PEX synthesis at a higher scale, small aliquots (5 μL) of all samples were mixed with 5 μL of PAGE stop solution and heated for 5 min at 95 °C prior to loading. Samples were separated with a 12.5 % PAGE under denaturing conditions (42 mA, 30 min). Visualisation was performed using fluorescence imaging (Figure S11-B, Figure S12-B).

The remaining volume (~ 95 μL) of all samples was mixed with 20 μL of hybridization solution (H7140, Sigma Aldrich) and freezed to - 80 °C.

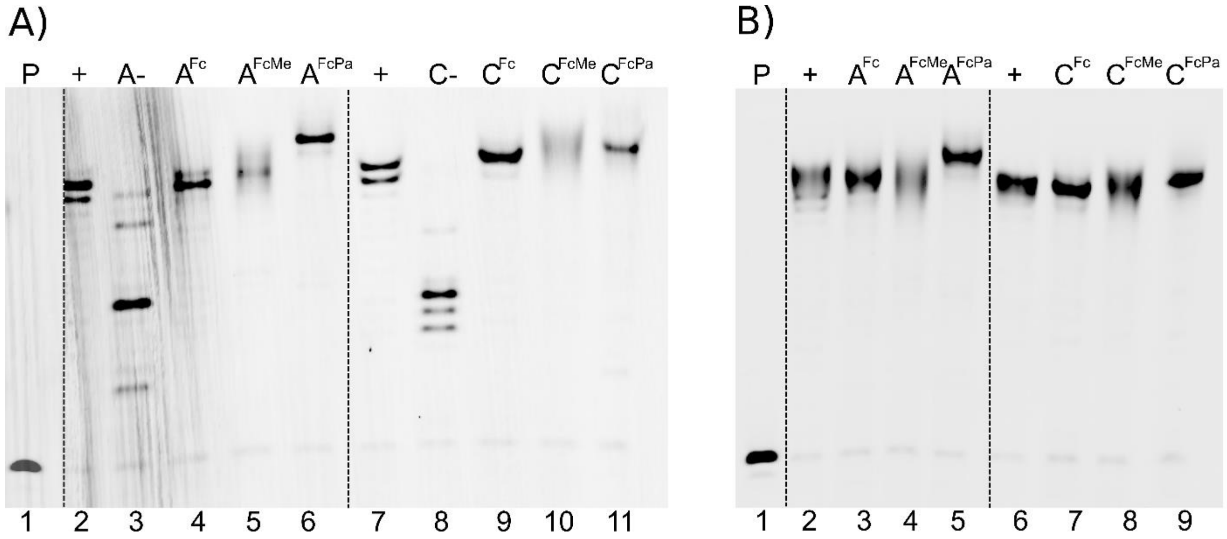

Figure S11. Primer extension with KOD XL DNA polymerase using prim^{rnd} and **temp$^{2\text{-}C3\text{-}rnd16}$**: A) PEX in small scale (20 μL); B) PEX in larger scale (100 μL); (P) primer (5'-6-FAM-labelled); (+) natural dNTPs; (A-) dCTP, dTTP, dGTP; (C-) dATP, dTTP, dGTP; (A^{Fc}) **dA^{Fc}TP**, dCTP, dTTP, dGTP; (A^{FcMe}) **dA^{FcMe}TP**, dCTP, dTTP, dGTP; (A^{FcPa}) **dA^{FcPa}TP**, dCTP, dTTP, dGTP; (C^{Fc}) **dC^{Fc}TP**, dATP, dTTP, dGTP; (C^{FcMe}) **dC^{FcMe}TP**, dATP, dTTP, dGTP; (C^{FcPa}) **dC**^{FcPa}**TP**, dATP, dTTP, dGTP.

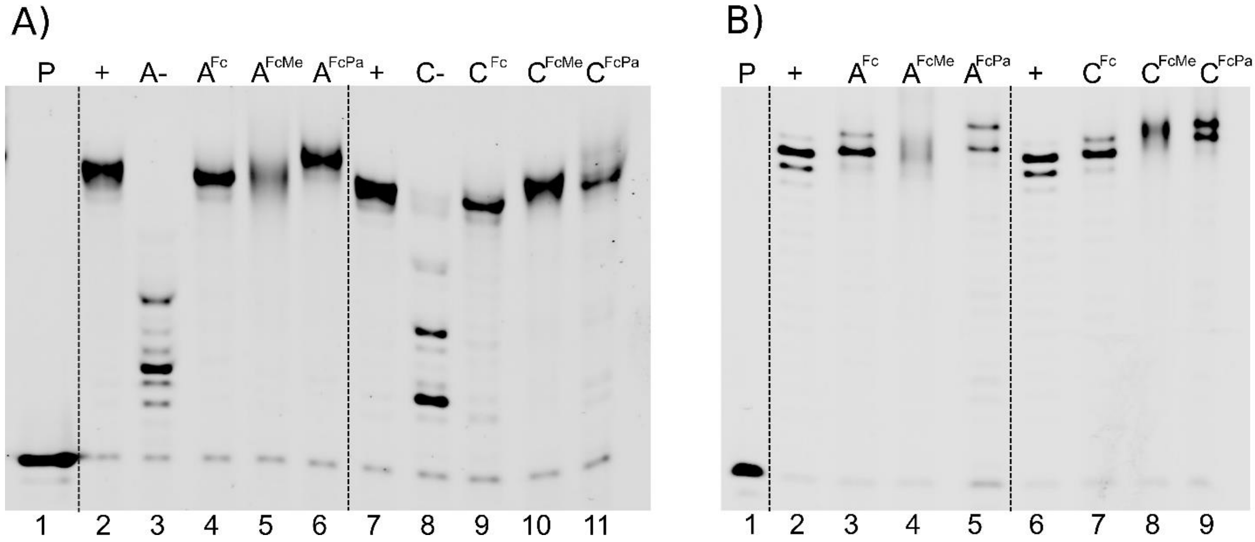

Figure S12. Primer extension with KOD XL DNA polymerase using prim^{rnd} and **temp$^{3\text{-}C3\text{-}Repet4*A,C}$**: A) PEX in small scale (20 μL); B) PEX in larger scale (100 μL); (P) primer (5'-6-FAM-labelled); (+) natural dNTPs; (A-) dCTP, dTTP, dGTP; (C-) dATP, dTTP, dGTP; (A^{Fc}) **dA^{Fc}TP**, dCTP, dTTP, dGTP; (A^{FcMe}) **dA^{FcMe}TP**, dCTP, dTTP, dGTP; (A^{FcPa}) **dA^{FcPa}TP**, dCTP, dTTP, dGTP; (C^{Fc}) **dC^{Fc}TP**, dATP, dTTP, dGTP; (C^{FcMe}) **dC^{FcMe}TP**, dATP, dTTP, dGTP; (C^{FcPa}) **dC**^{FcPa}**TP**, dATP, dTTP, dGTP.

Polymerase chain reaction (PCR) with modified dNTPs

The PCR was performed with a C1000Touch thermal cycler (Biorad) using following cycling protocol: 95 °C for 3 min, followed by 30 cycles at 94 °C for 1 min, 55 °C for 1 min, and 72 °C for 1 min, followed by a final elongation step at 72 °C for 5 min.

A) The PCR reaction mixture (20 μL) contained KOD XL DNA Polymerase (2.5 U/μL, 0.8 μL), natural dNTPs (4 mM, 1 μL), modified dATPs or dCTPs (4 mM, 2 μL), forward primer^{LT25TH} and reverse primer^{L20-} (100 μM, 0.2 μL each) and a 98-mer template temp^{FVLA} (10 nM, 2 μL,) in KOD XL reaction buffer (2 μL) as supplied by the manufacturer. PCR products were separated with a 2 % agarose gel in 0.5X TBE buffer, stained with GelRed (Figure S13-A).

B) The PCR reaction mixture (20 μL) contained KOD XL DNA Polymerase (2.5 U/μL, 0.8 μL), natural dNTPs (4 mM, 1 μL), modified dA^{X}TPs or dC^{X}TPs (X = Fc, FcMe, FcPA, 4 mM each) whilst maintaining 1.5:1 ratio of **dA^{X}TP:dATP** or **dC^{X}TP:dCTP** (1.2 μL of dN^{X}TP + 0.8 μL of dNTP), forward primer^{LT25TH} and reverse primer^{L20-} (100 μM, 0.2 μL each) and a 98-mer template temp^{FVLA} (10 nM, 2 μL,) in KOD XL reaction buffer (2 μL) as supplied by the manufacturer. PCR products were separated with a 2 % agarose gel in 0.5X TBE buffer, stained with GelRed. (Figure S13-B)

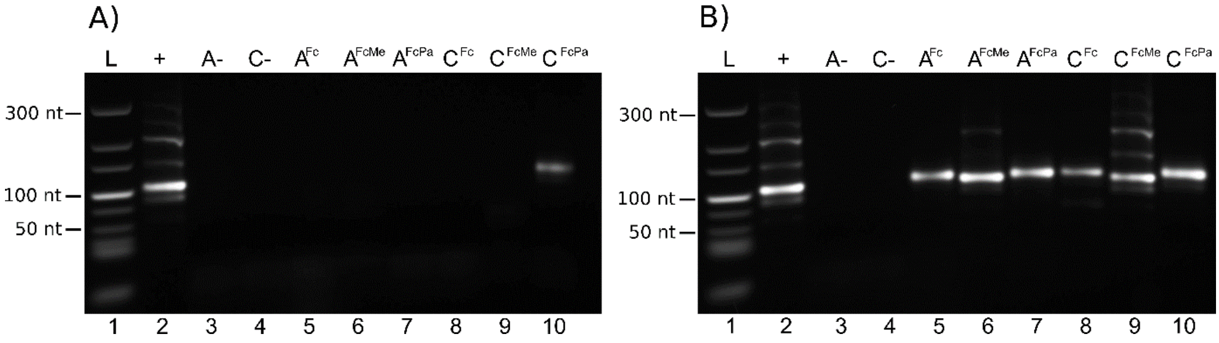

Figure S13. PCR synthesis with KOD XL DNA polymerase using 98-mer temp^{FVLA}. A + B) Lane 1 (L): ladder; lane 2 (+): natural dNTPs; lane 3 (A-): dTTP, dCTP, dGTP; lane 4 (C-): dTTP, dATP, dGTP;
A) lane 5 (A^{Fc}) **dA^{Fc}TP**, dCTP, dTTP, dGTP; lane 6 (A^{FcMe}) **dA^{FcMe}TP**, dCTP, dTTP, dGTP; lane 7 (A^{FcPa}) **dA^{FcPa}TP**, dCTP, dTTP, dGTP; lane 8 (C^{Fc}) **dC^{Fc}TP**, dATP, dTTP, dGTP; lane 9 (C^{FcMe}) **dC^{FcMe}TP**, dATP, dTTP, dGTP; lane 10 (C^{FcPa}) **dC**^{FcPa}**TP**, dATP, dTTP, dGTP.
B) Lane 5 (A^{Fc}) **dA^{Fc}TP:dATP** (1.5:1), dCTP, dTTP, dGTP; lane 6 (A^{FcMe}) **dA^{FcMe}TP:dATP** (1.5:1), dCTP, dTTP, dGTP; lane 7 (A^{FcPa}) **dA^{FcPA}TP:dATP** (1.5:1), dCTP, dTTP, dGTP; lane 8 (C^{Fc}) **dC^{Fc}TP:dCTP** (1.5:1), dATP, dTTP, dGTP; lane 9 (C^{FcMe}) **dC^{FcMe}TP:dCTP** (1.5:1), dATP, dTTP, dGTP; lane 10 (C^{FcPa}) **dC^{FcMe}TP:dCTP** (1.5:1), dATP, dTTP, dGTP.

4. Experimental section – electrochemistry

Electrochemical analysis of nucleosides

Modified nucleosides and dNTPs were studied by *in situ* square-wave voltammetry (SWV) at a basal plane-oriented pyrolytic graphite electrode (PGE). SWV settings: initial potential -0.5 V, end potential 1.6 V, frequency 200 Hz, amplitude 50 mV; background electrolyte: 0.2 M sodium acetate pH 5.0. Concentration of the compounds was 40 μM. All measurements were performed at room temperature using an Autolab analyzer (Eco Chemie, The Netherlands) in connection with VA-stand 663 (Metrohm, Herisau, Switzerland). The three-electrode system was used with Ag/AgCl/3M KCl electrode as a reference and platinum wire as an auxiliary electrode (Figure S14-S15).

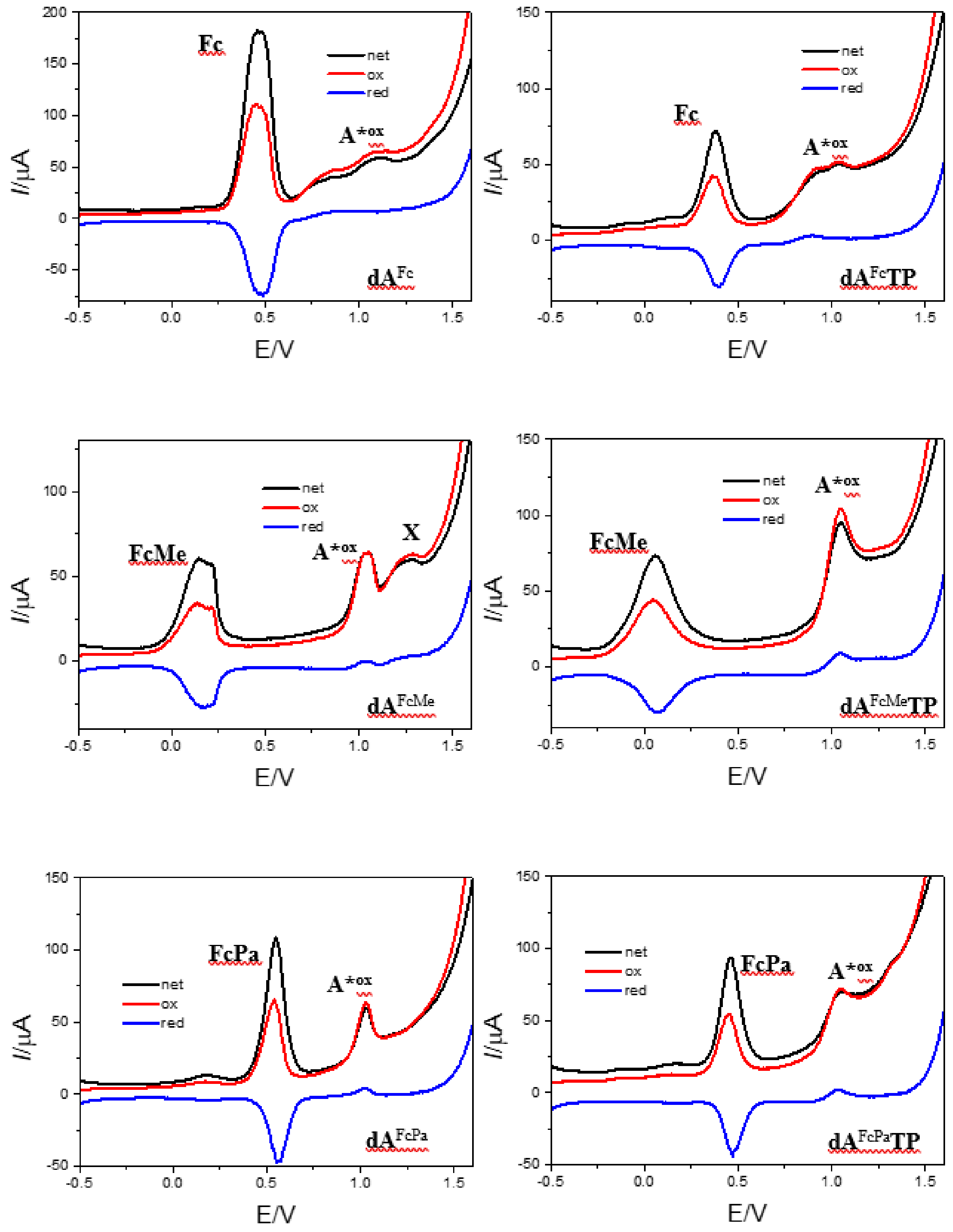

Figure S14. Components of square-wave voltammograms of A^{FcMe} and A^{FcPa}-modified nucleosides and dNTPs (for experimental conditions see Figure 2 in the article). Red curves – forward component, blue – backward component, black – net current. Negative (reduction) counterpeaks on the backward curve at potentials close to those of forward positive (oxidation) peaks indicate reversibility of the system (ferrocene/ferrocenium). Absence of the negative peaks (or presence of small positive peaks at the backward curve) indicate an irreversible oxidation process.

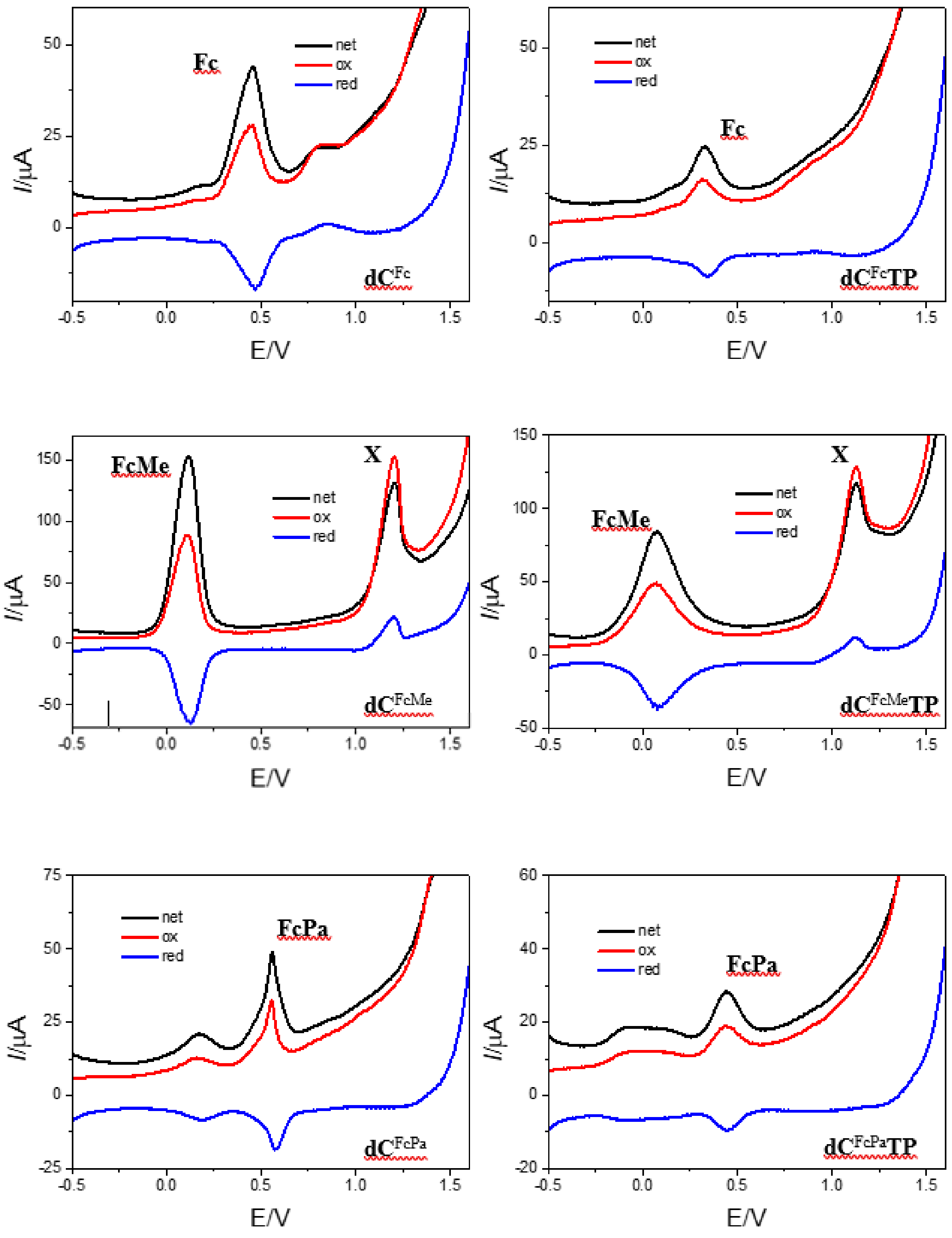

Figure S15. Components of square-wave voltammograms of **C**$^{\mathbf{FcMe}}$ and **C**$^{\mathbf{FcPa}}$-modified nucleosides and dNTPs (for experimental conditions see Figure 2 in the article). Red curves – forward component, blue – backward component, black – net current. Negative (reduction) counterpeaks on the backward curve at potentials close to those of forward positive (oxidation) peaks indicate reversibility of the system (ferrocene/ferrocenium). Absence of the negative peaks (or presence of small positive peaks at the backward curve) indicate an irreversible oxidation process.

Electrochemical analysis of oligonucleotides

Electrode fabrication and functionalisation

Gold sputtered electrode arrays consisting of 3 groups of 3 circular working electrodes (1 mm^2), were fabricated on a glass substrate as described previously.[3] Prior to functionalisation, the electrodes were cleaned by sonicating for 5 minutes in acetone, followed by 5 minutes in isopropanol and were then rinsed with Milli-Q water and dried with N_2. Subsequently, the electrodes were electrochemically cleaned in KOH (0.1 M) using cyclic voltammetry with a scan rate of 100 mV/s, from 0 to -1.2 V vs Ag/AgCl, for 10 scans. The electrodes were then rinsed with Milli-Q water, dried with N_2, and again electrochemically cleaned, this time in H_2SO_4 (0.1 M), with a scan rate of 100 mV/s from 0 to 1.6 V vs Ag/AgCl, again for 10 cycles, before a final rinse with Milli-Q water and drying with N_2.

A capture probe cocktail solution was prepared to contain 1 µM capture probe, 100 µM of mercaptohexanol and 1 M KH_2PO_4. One microlitre of the capture probe cocktail was dropcast on to each working electrode and the array was then incubated for 3 h at 22 ºC in a humidity saturated chamber. Following functionalisation, the electrodes were rinsed with Milli-Q and then dried with N_2. A 7 µL volume chamber was created to host the PEX purified products for hybridisation and electrochemical detection, by placing a PMMA cover over a patterned double adhesive gasket. Once assembled, the microfluidic chambers were washed with 20 µL of PBS Tween-20, then 200 µL of milli-Q and finally dried them with N_2 prior to use.

Primer extension and hybridisation

Primer extension (PEX) was performed in a T100 thermal cycler (Biorad) by heating the sample to 60 ºC for 30 minutes. The PEX reaction mixture (20 µL) contained 1.2 units of KOD XL DNA polymerase, KOD XL buffer (1X), 200 nM primer, 300 nM template and 200 µM of dGTP, dTTP, **dA^{Fc}TP** and **dC^{FcPa}TP**. The PEX product was mixed with 4 µL of hybridisation solution (2X) and 7 µL of the mixture were added to a functionalised electrode array and incubated for 30 minutes at 22 ºC in a humidity chamber. The microfluidic chambers were then washed with 3 x 200 µL of PBS Tween-20 and 200 µL of 0.1 M $Ca(NO_3)_2$.

Agarose gel electrophoresis

PEX products were visualised using agarose gel electrophoresis, which was prepared with ultra low pure agarose (4 % w/v) in 1× Tris-Borate-EDTA buffer (TBE) and stained with GelRed™nucleic acid stain. PEX product (3 μL) was added to 3 μL of loading buffer 2X (stock buffer 6X contained glycerol 30 %, bromphenol blue 0,25 %) per well, and electrophoresis was carried out with an applied voltage of 100 V for 40 min. The DNA was imaged using a UV transilluminator (λ = 254 nm) (Figure S16).

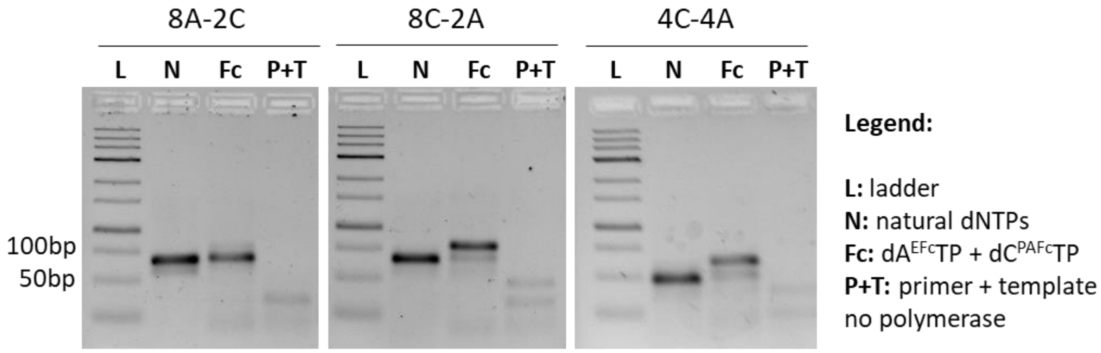

Figure S16. Agarose gel electrophoresis of PEX products using temp^{8A,2C}, temp^{8C,2A} or temp^{4C,4A} templates and primer$^{8/2_4/4}$ obtained in the presence of (a) all four natural dNTPs (+), or combination of **dA^{Fc}TP**, **dC^{FcPa}TP** with TTP and GTP (*Fc). P/T corresponds to primer hybridised to template in the absence of polymerase.

Electrochemical measurements of PEX products

Square wave voltammetry was performed using a potentiostat/galvanostat PBSTAT 12 Autolab controlled with Nova 2.1.3 software. Following hybridisation, measurements were carried out in 0.1 M $Ca(NO_3)_2$ electrolyte solution using an external Ag/AgCl reference electrode and the internal counter and working gold electrodes on the electrode array, with a step potential from 0 V to 0.6 V using a 1 mV step, 20 mV modulation amplitude and 50 Hz of frequency.

Hybridisation for liquid phase PEX products

PEX product (7 μL) was added to each electrode array chamber (3 working electrodes/chamber, for triplicate measurements) for 30 minutes at 22 ºC in a humidity saturated chamber. Following hybridisation the electrode array chambers were flushed with 3 x 200 μL of PBS-Tween and then with 200 μL of 0.1 M $Ca(NO_3)_2$.

Electrochemical detection - Square wave voltammetry

Square wave voltammetry was carried out using a potentiostat/galvanostat PGSTAT 12 Autolab controlled with Nova 2.1.3 software. Following hybridisation, measurements were taken in 0.1 M $Ca(NO_3)_2$ electrolyte solution using an external Ag/AgCl reference electrode and the internal counter and working gold electrodes on the electrode array. For square wave voltammetry experiments, a step potential from 0.2 V to 0.6 V was used, with a 5 mV step, 25 mV modulation amplitude and 50 Hz frequency. For cyclic voltammetry experiments the potential was cycled between 0 and 0.6 V vs Ag/AgCl at a scan rate of 100 mV/s.

5. Copies of MALDI-TOF mass spectra

a) prim^{rnd} 3'-GGGTACGGCGGGTAC-5'

temp^{rnd16} 5'-CTAGCATGAGCTCAGTCCCATGCCGCCCATG-3'

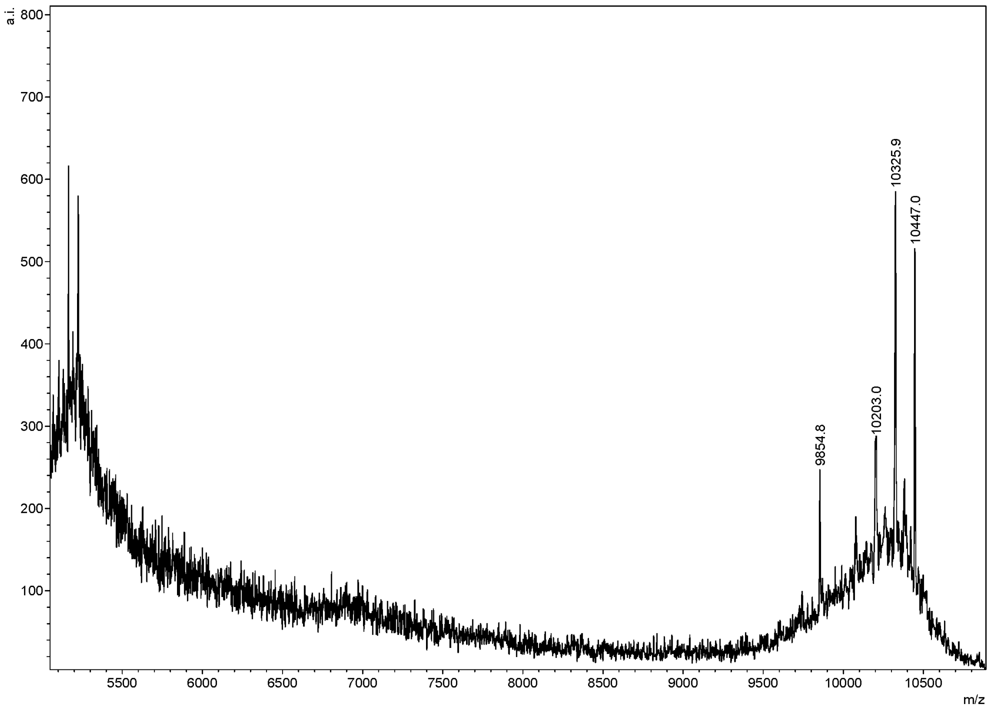

Figure S17. Mass (ssDNA: **dA^{Fc}TP,** dCTP, dTTP, dGTP): calculated: 10445,54 Da; found: 10447.0 Da; Δ = 1.46 (M = 10325.9 Da is full product minus one Cp ring with Fc; M = 10203.0 Da is full product minus two Cp rings with two Fc)

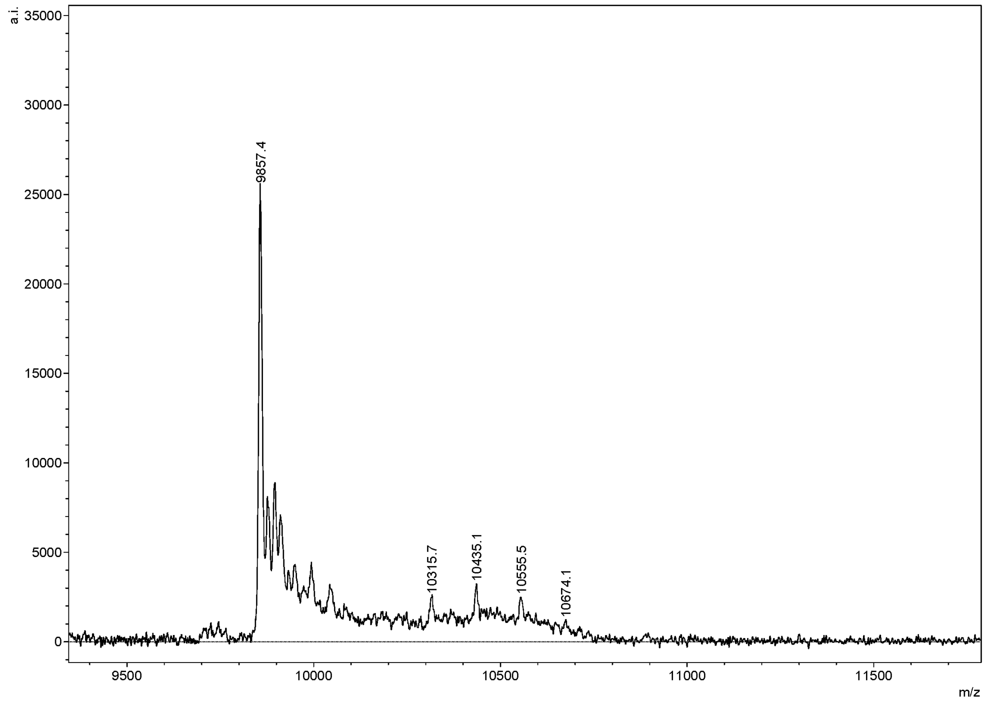

Figure S18. Mass (ssDNA: **dA^{FcPa}TP,** dCTP, dTTP, dGTP): calculated: 10673,7 Da; found: 10674.1 Da; Δ = 1.46 (M = 9857.4 Da is a peak of template; M = 10555.5 Da is full product

minus one Cp ring with Fc; M = 10435.0 Da is full product minus two Cp rings with two Fc; M = 10315.7 Da is full product minus three Cp ring with three Fc) (Sample was measured in linear mode).

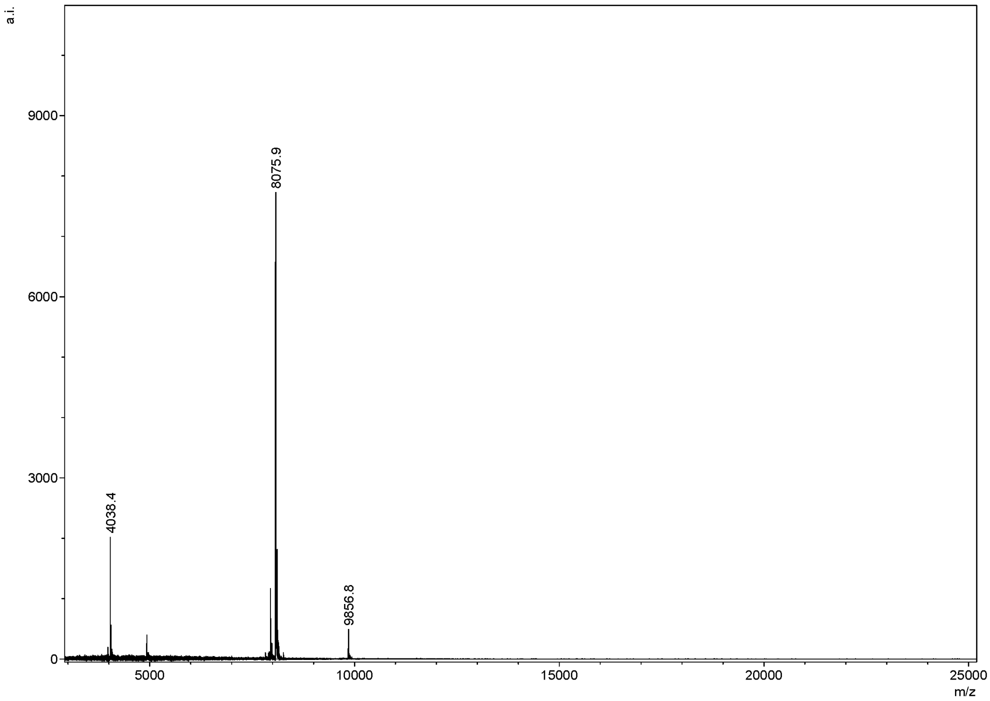

Figure S19. Mass (ssDNA: **dA^{FcMe}TP,** dCTP, dTTP**,** dGTP): calculated: 10894,38 Da; found: 8075.9 Da; (M = 9856.8 Da is a peak of template; M = 8075.9 Da is product of not fully incorporated product 5'-CATGGGCGGCATGGG**A**^{FcMe}CTG**A**^{FcMe}GCTC-3')

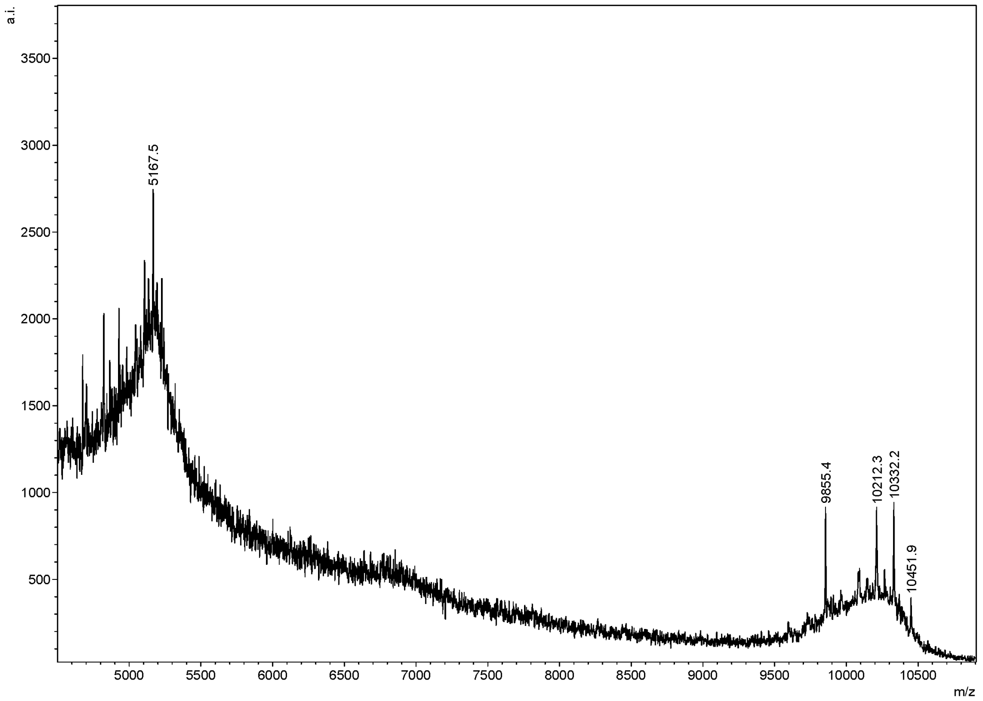

Figure S20. Mass (ssDNA: **dC^{Fc}TP,** dATP, dTTP**,** dGTP): calculated: 10449,54 Da; found: 10451.9 Da; Δ = 2.36 (M = 10332.2 Da is full product minus one Cp ring with Fc; M = 10212.3 Da is full product minus two Cp rings with two Fc).

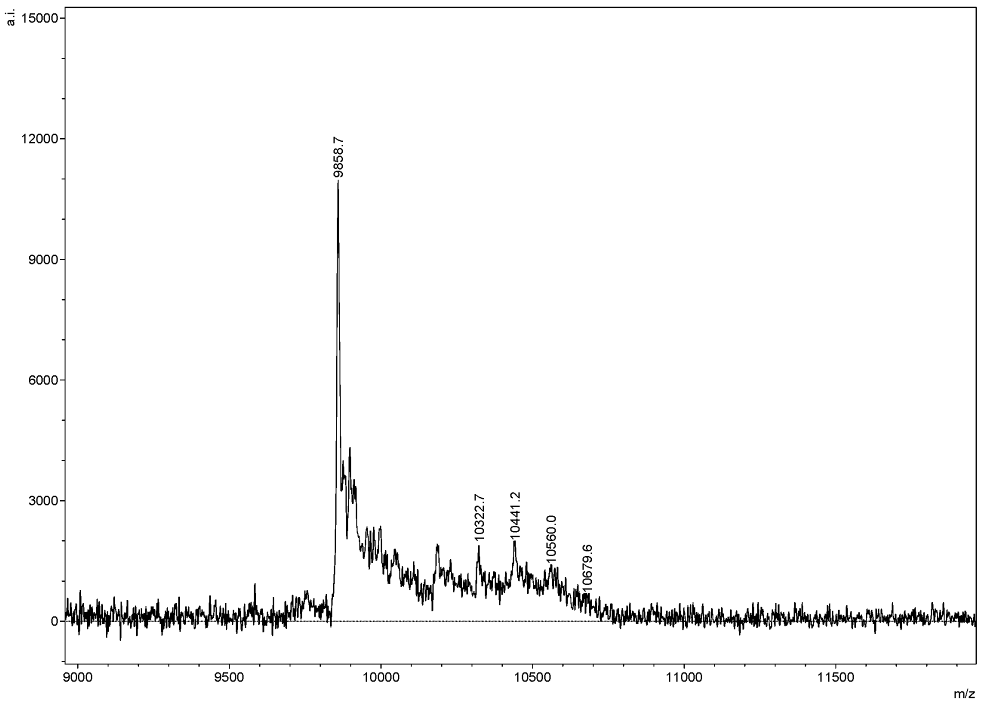

Figure S21. Mass (ssDNA: **dC^{FcPa}TP,** dATP, dTTP**,** dGTP): calculated: 10677,7 Da; found: 10679.6 Da; Δ = 1.9 (M = 9858.7 Da is a peak of template; M = 10560.0 Da is full product minus one Cp ring with Fc; M = 10441.2 Da is full product minus two Cp rings with two Fc; M = 10322.7 Da is full product minus three Cp ring with three Fc) (Sample was measured in linear mode)

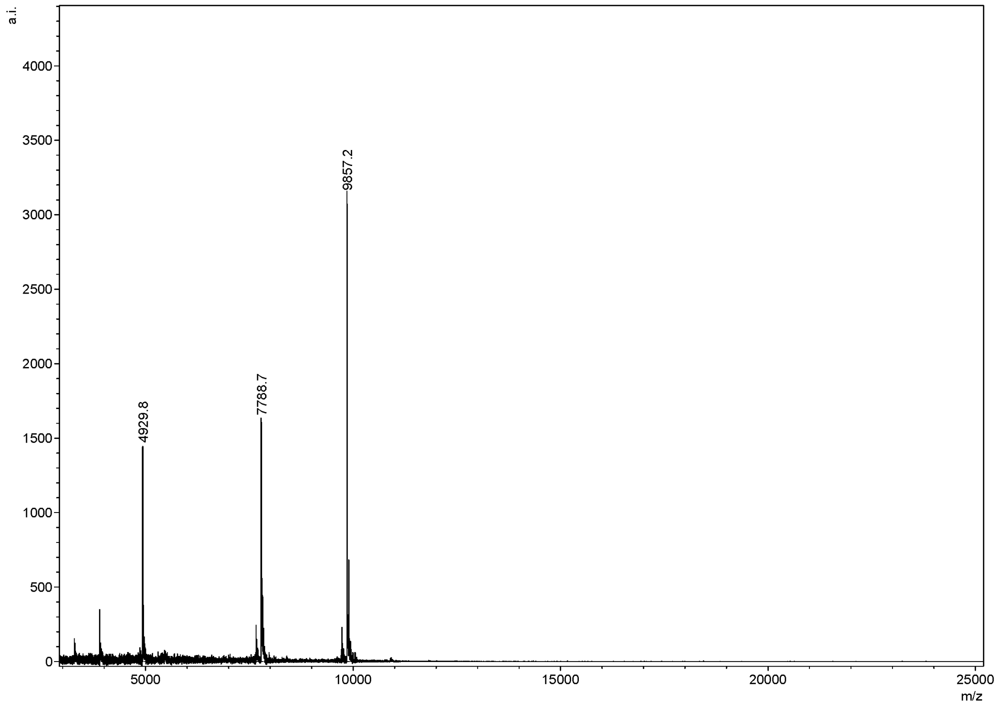

Figure S22. Mass (ssDNA: dC^{FcMe}TP, dATP, dTTP**,** dGTP): calculated: 10898,38 Da; found: 7788.7 Da; (M = 9857.2 Da is a peak of template; M = 7788.7 Da is product of not fully incorporated product 5’-CATGGGCGGCATGGGA**C**^{FcMe}TGAG**C**^{FcMe}T-3’)

b) prim^{rnd} 3'-GGGTACGGCGGGTAC-5'

temp^{A} 5'-CCCTCCCATGCCGCCCATG-3'

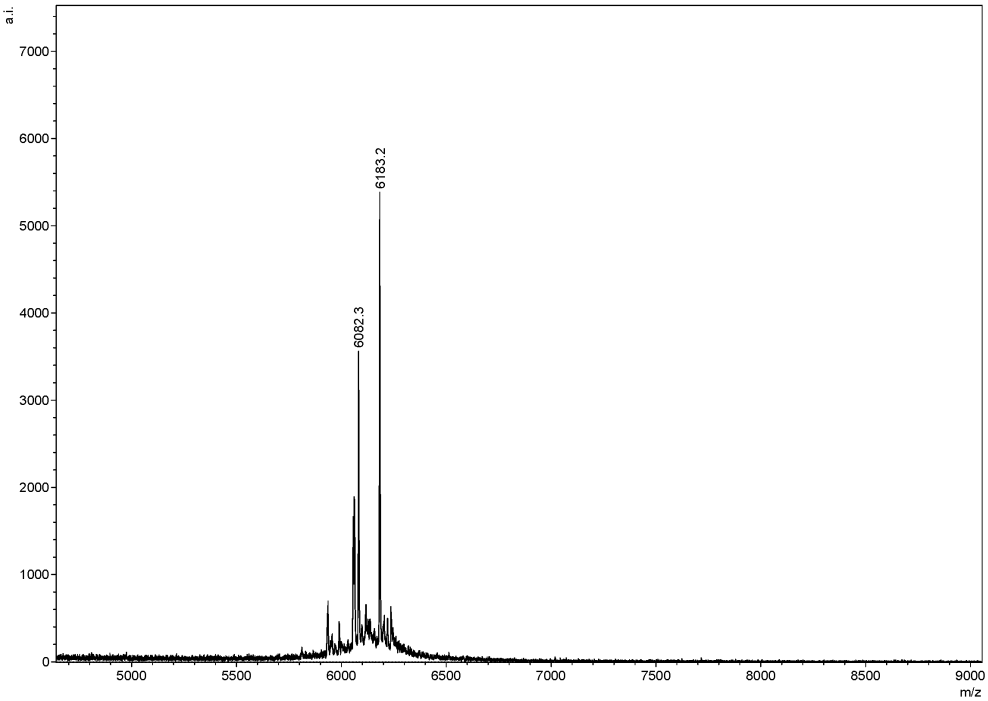

Figure S23. Mass (ssDNA: **dA^{Fc}TP,** dGTP): calculated: 6182,03 Da; found: 6183.2 Da; Δ = 1.17 (M = 6082.3 Da is a peak of template)

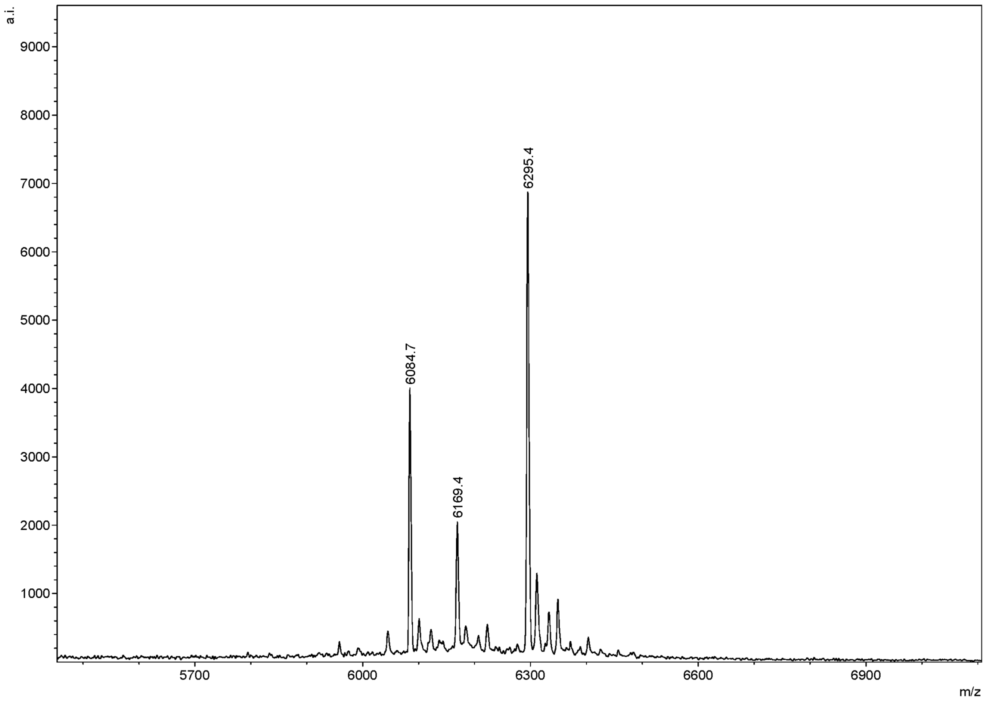

Figure S24. Mass (ssDNA: **dA^{FcMe}TP,** dGTP): calculated: 6293,96 Da; found: 6295.4 Da; Δ = 1.44

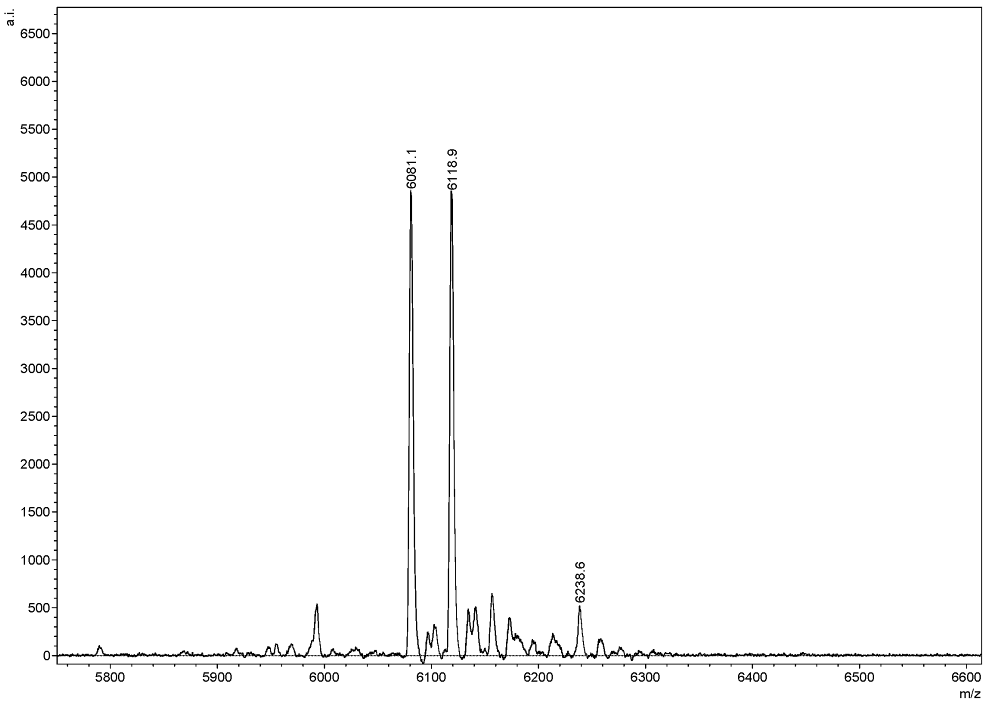

Figure S25. Mass (ssDNA**: dA^{FcPa}TP,** dGTP): calculated: 6239,01 Da; found: 6238.6 Da; Δ = 0.41 (M = 6081.1 Da is a peak of template; M = 6118.9 Da is full product minus Cp ring with Fc)

c) prim^{rnd} 3'-GGGTACGGCGGGTAC-5'

temp^{C} 5'-CCCGCCCATGCCGCCCATG-3'

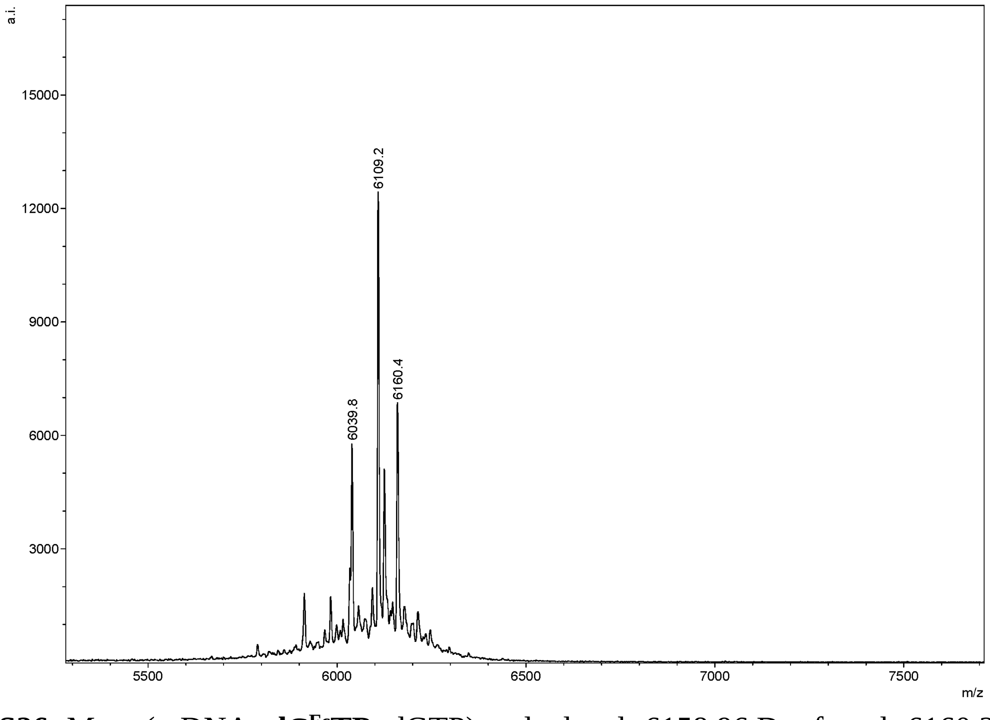

Figure S26. Mass (ssDNA: **dC^{Fc}TP,** dGTP): calculated: 6158,96 Da; found: 6160.2 Da; Δ = 1.24 (M = 6109.2 Da is a peak of template; M = 6039.8 Da is full product minus Cp ring with Fc).

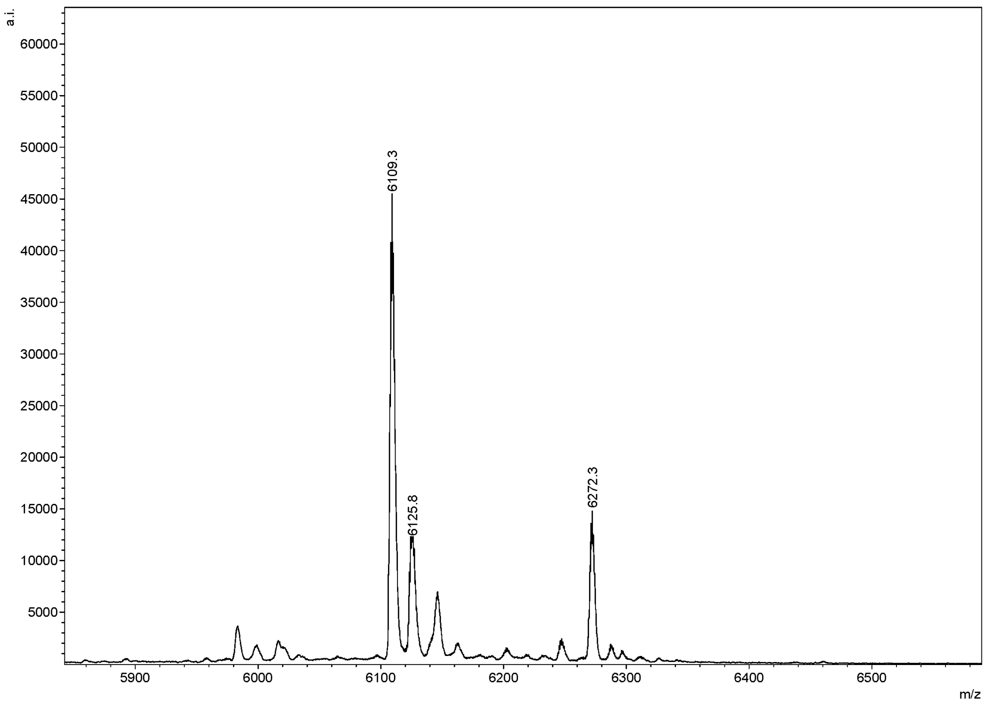

Figure S27. Mass (ssDNA: **dC^{FcMe}TP,** dGTP): calculated: 6270,96 Da; found: 6272.3 Da; Δ = 1.34.

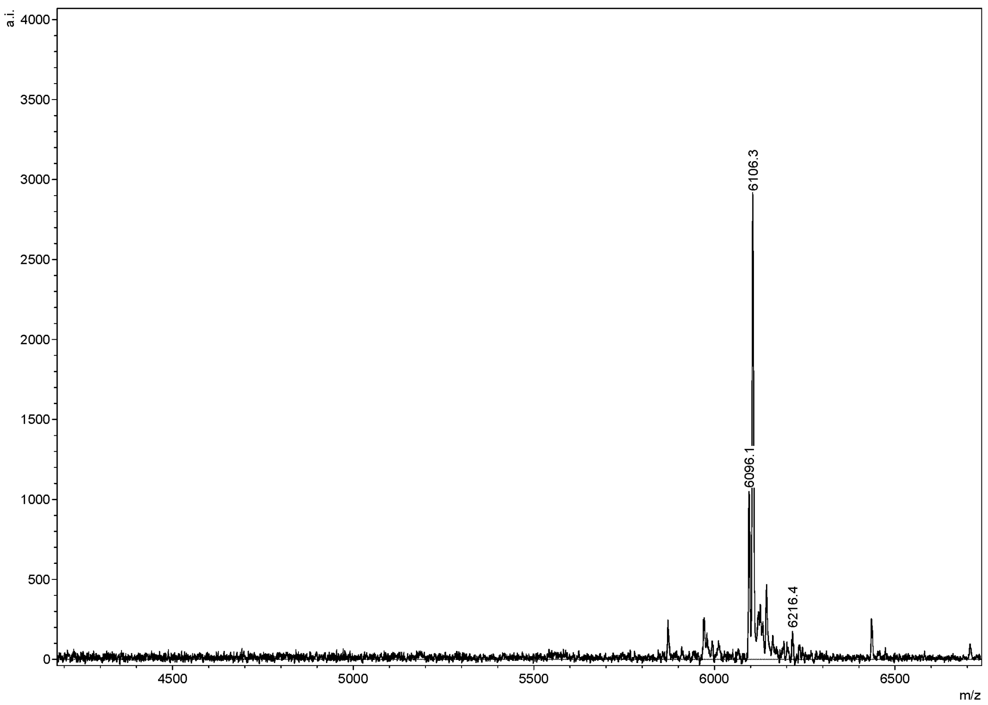

Figure S28. Mass (ssDNA: **dC^{FcPa}TP,** dGTP): calculated: 6216,01 Da; found: 6216.4 Da; Δ = 0.39 (M = 6106.3 Da is a peak of template; M = 6096.1 Da is full product minus Cp ring with Fc).

6. Copies of NMR spectra

dA^{FcMe}

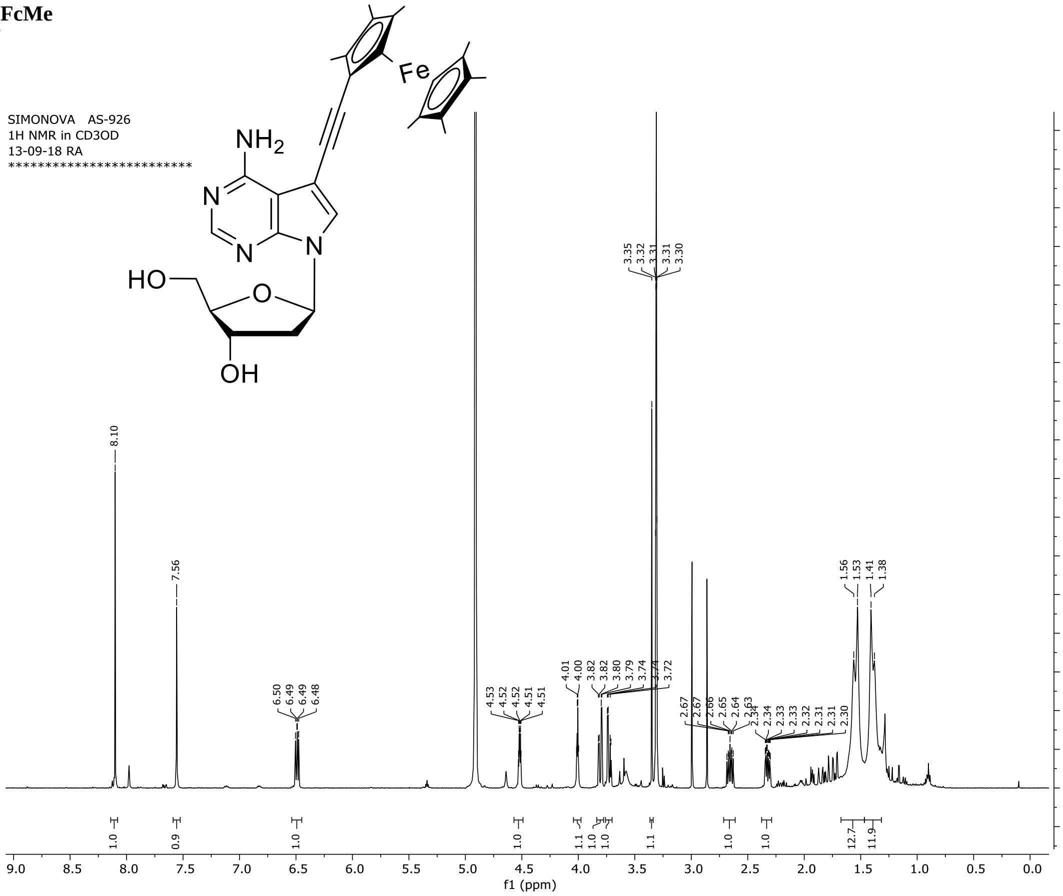

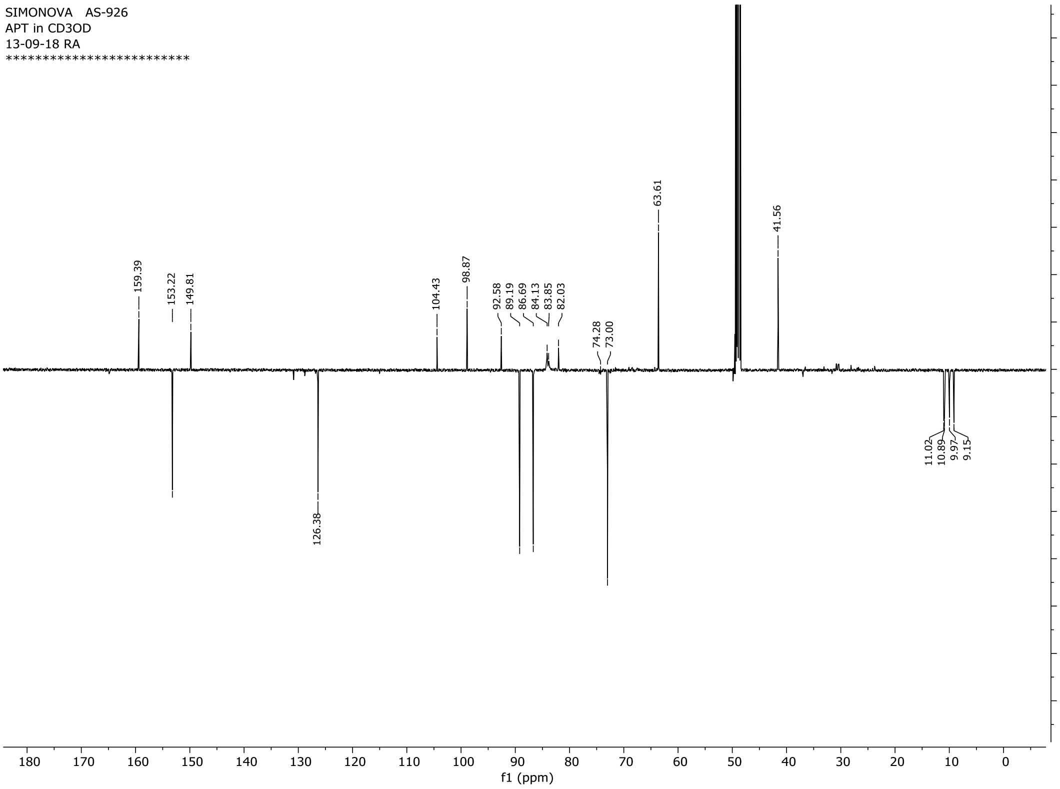

dC^{FcMe}

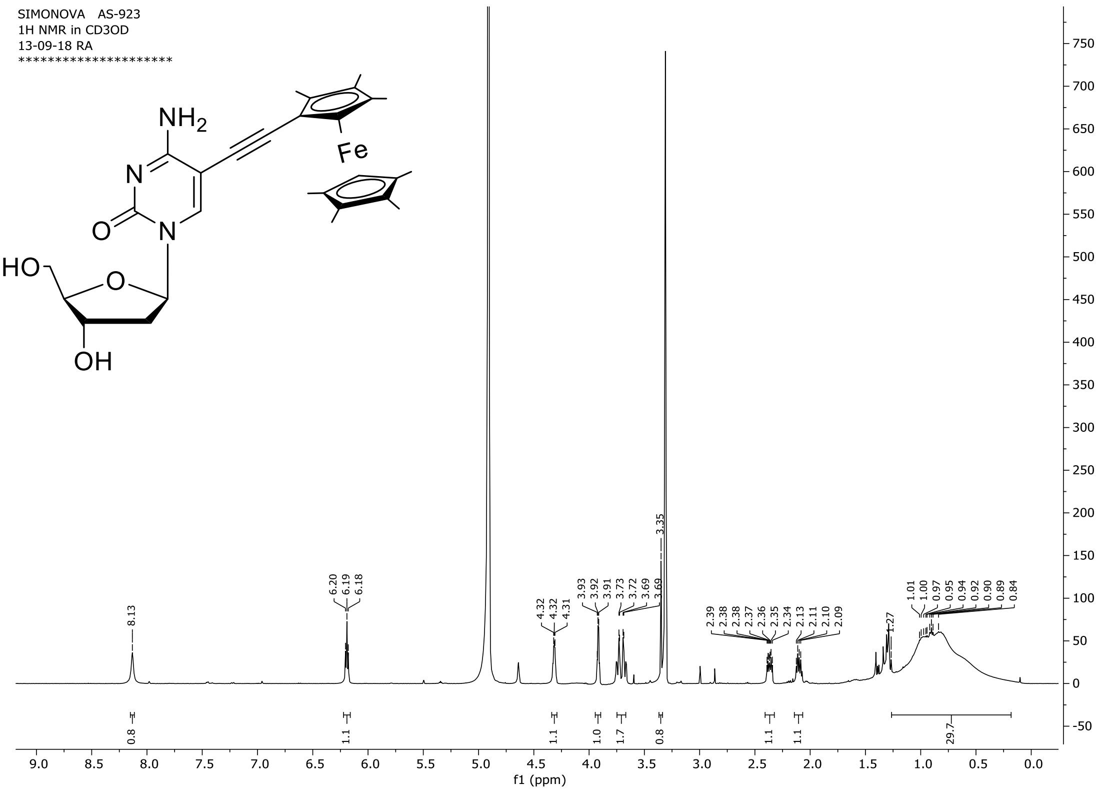
SIMONOVA AS-923
1H NMR in CD3OD
13-09-18 RA
NH2
N
O
N
HO
O
OH
Fe
f1 (ppm)

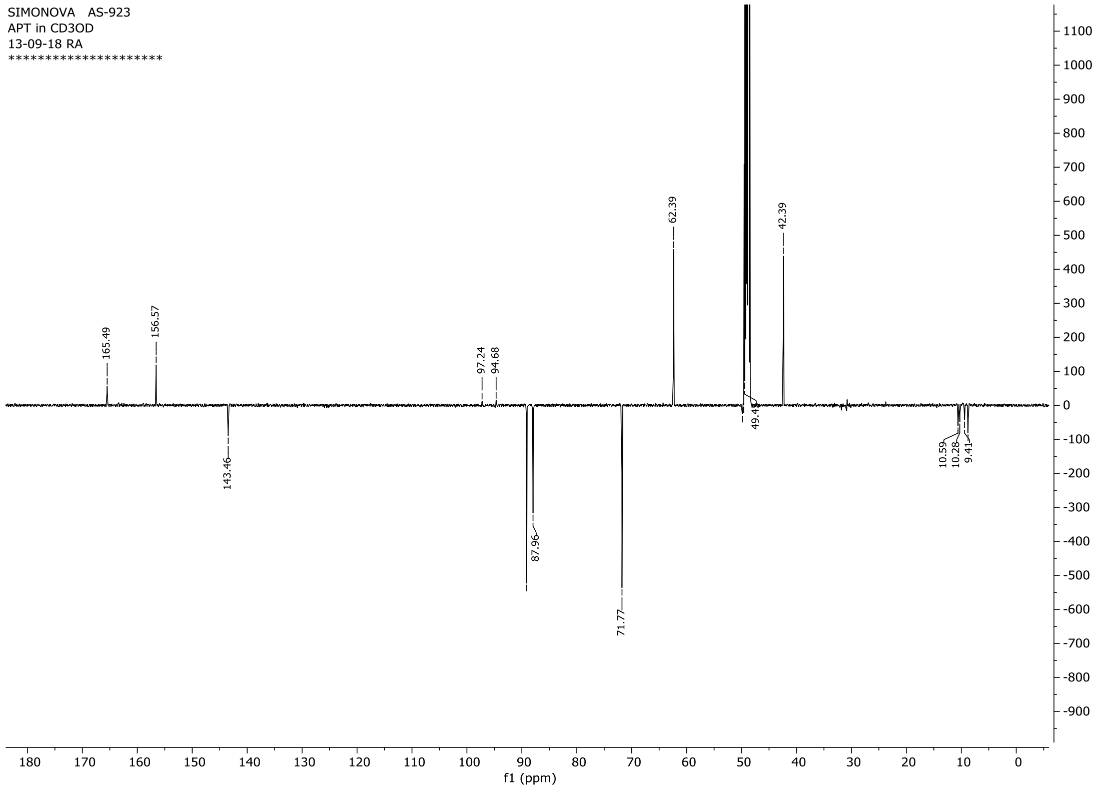
SIMONOVA AS-923
APT in CD3OD
13-09-18 RA
f1 (ppm)

dA^{FcPa}

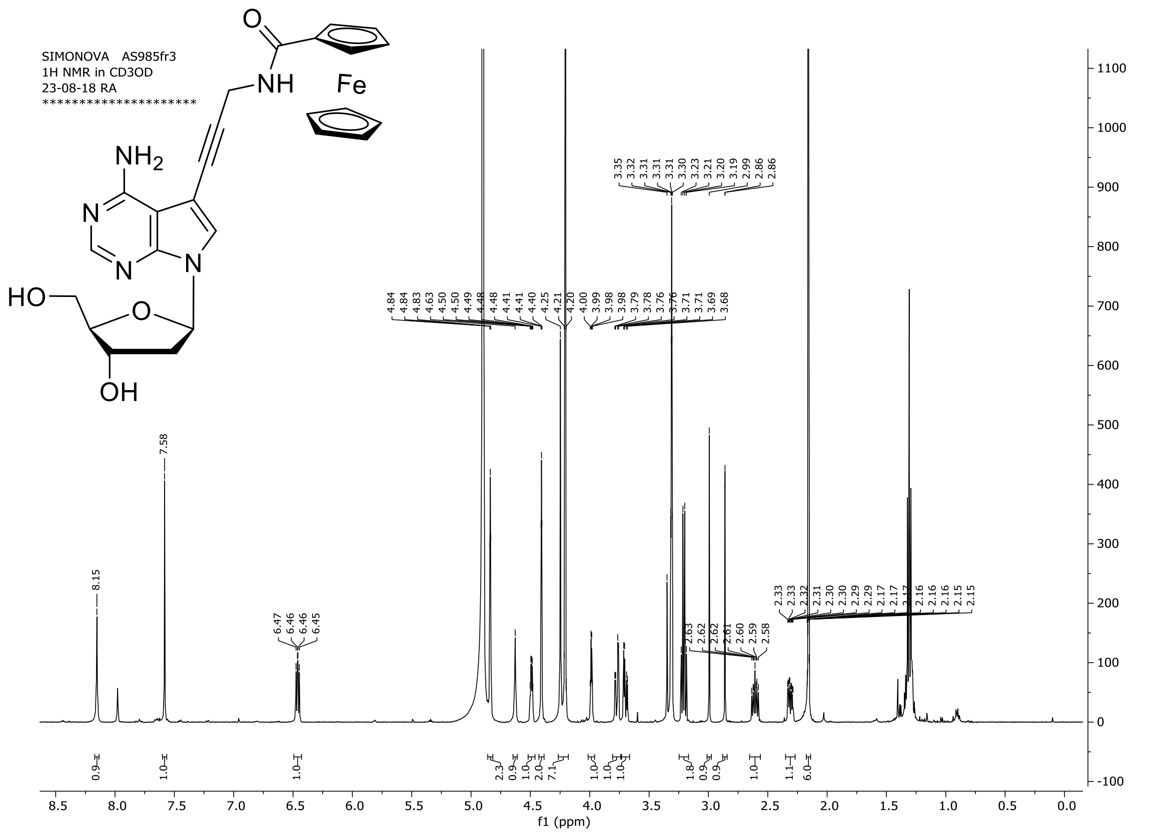
SIMONOVA AS985fr3
1H NMR in CD3OD
23-08-18 RA
****
O
NH
Fe
NH2
N
N
N
HO
O
OH
8.15
7.58
6.47 6.46 6.46 6.45
4.84 4.84 4.83 4.63 4.50 4.50 4.49 4.48 4.48 4.41 4.41 4.40 4.25 4.21 4.20 4.00 3.99 3.98 3.98 3.79 3.78 3.76 3.76 3.71 3.71 3.69 3.68
3.35 3.32 3.31 3.31 3.31 3.30 3.30 3.23 3.21 3.20 3.19 2.99 2.86 2.86
2.63 2.62 2.62 2.61 2.60 2.59 2.58
2.33 2.33 2.32 2.31 2.30 2.30 2.29 2.29 2.17 2.17 2.16 2.16 2.15 2.15
0.9 1.0 1.0 2.3 0.9 1.0 2.0 7.1 1.0 1.0 1.0 1.8 0.9 0.9 1.0 1.1 6.0
f1 (ppm)

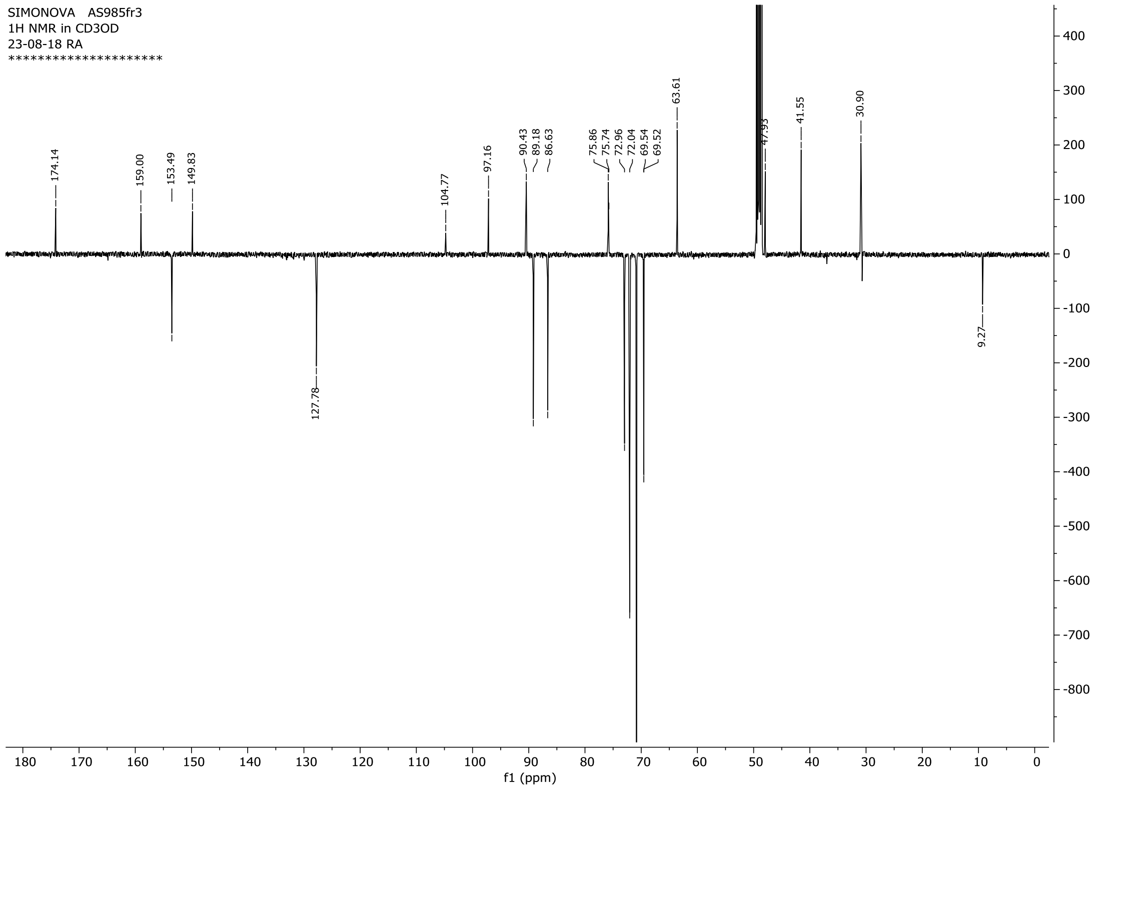
SIMONOVA AS985fr3
1H NMR in CD3OD
23-08-18 RA
****
174.14
159.00
153.49
149.83
127.78
104.77
97.16
90.43 89.18 86.63
75.86 75.74 72.96 72.04 69.54 69.52
63.61
47.93
41.55
30.90
9.27
f1 (ppm)

dC^{FcPa}

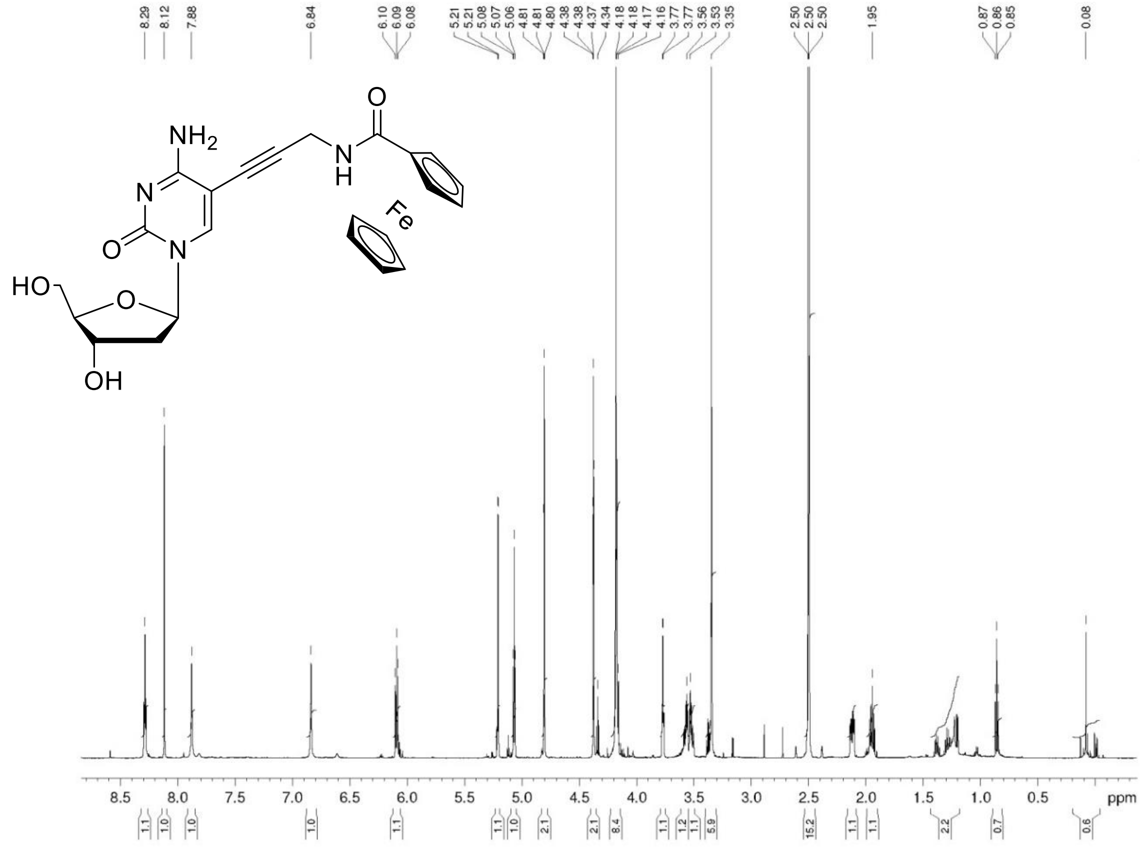
8.29 8.12 7.88 6.84 6.10 6.09 6.08 5.21 5.21 5.08 5.07 5.06 4.81 4.81 4.80 4.38 4.38 4.37 4.34 4.18 4.18 4.17 4.16 3.77 3.77 3.56 3.53 3.35 2.50 2.50 2.50 1.95 0.87 0.86 0.85 0.08
NH2
N
O
N
HO
O
OH
N
H
O
Fe
8.5 8.0 7.5 7.0 6.5 6.0 5.5 5.0 4.5 4.0 3.5 3.0 2.5 2.0 1.5 1.0 0.5 ppm
1.1 1.0 1.0 1.0 1.1 1.1 1.0 2.1 2.1 8.4 1.1 1.2 1.1 5.9 15.2 1.1 1.1 2.2 0.7 0.6

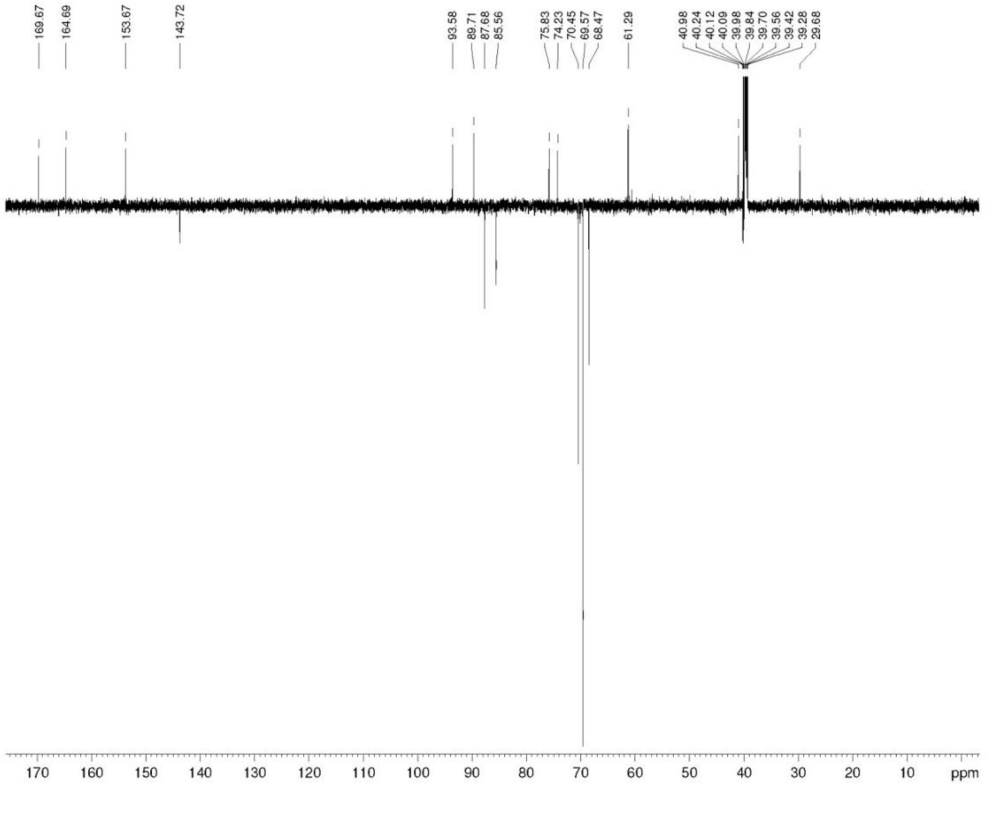
169.67 164.69 153.67 143.72 93.58 89.71 87.68 85.56 75.83 74.23 70.45 69.57 68.47 61.29 40.98 40.24 40.12 40.09 39.98 39.84 39.70 39.56 39.42 39.28 29.68
170 160 150 140 130 120 110 100 90 80 70 60 50 40 30 20 10 ppm

dA^{FcMe}TP

^{1}H and ^{13}C NMR spectra were not possible to analyse because of partial oxidation of ferrocene moiety.

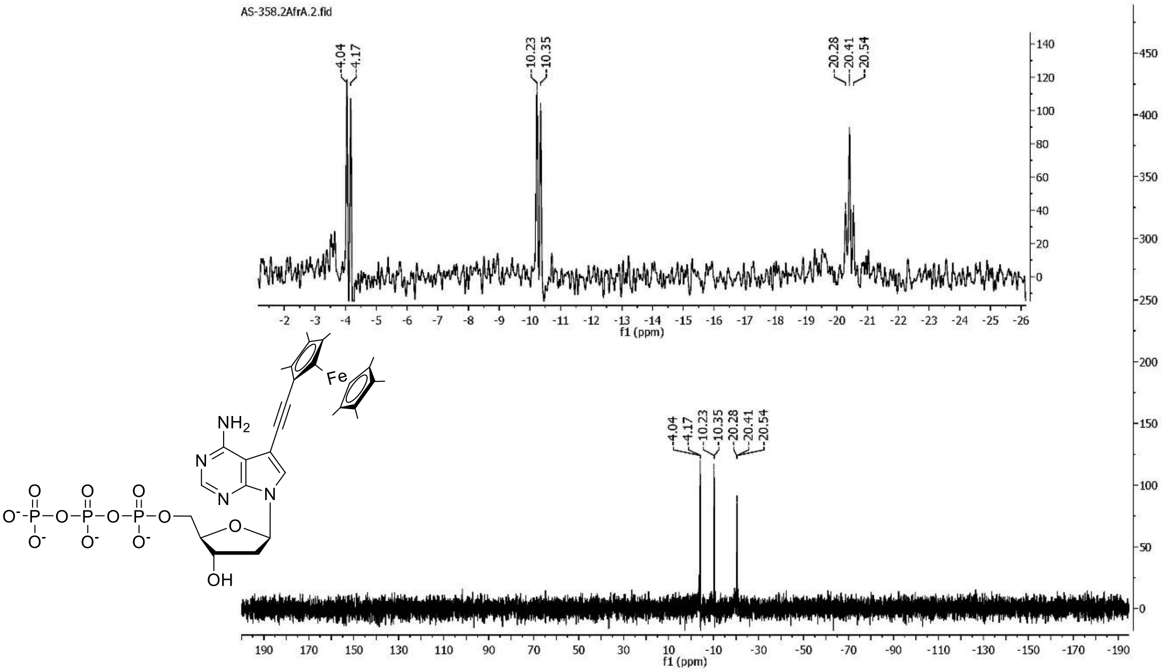

dC^{FcMe}TP

^{1}H and ^{13}C NMR spectra were not possible to analyse because of partial oxidation of ferrocene moiety.

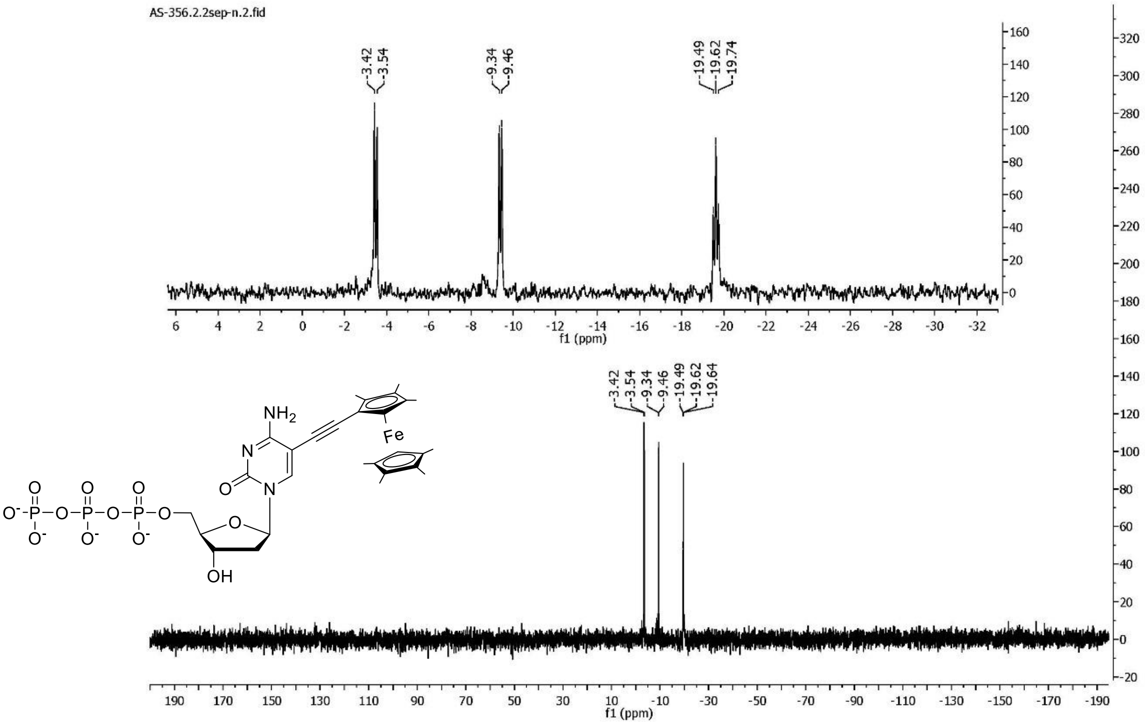

dA^{FcPa}TP

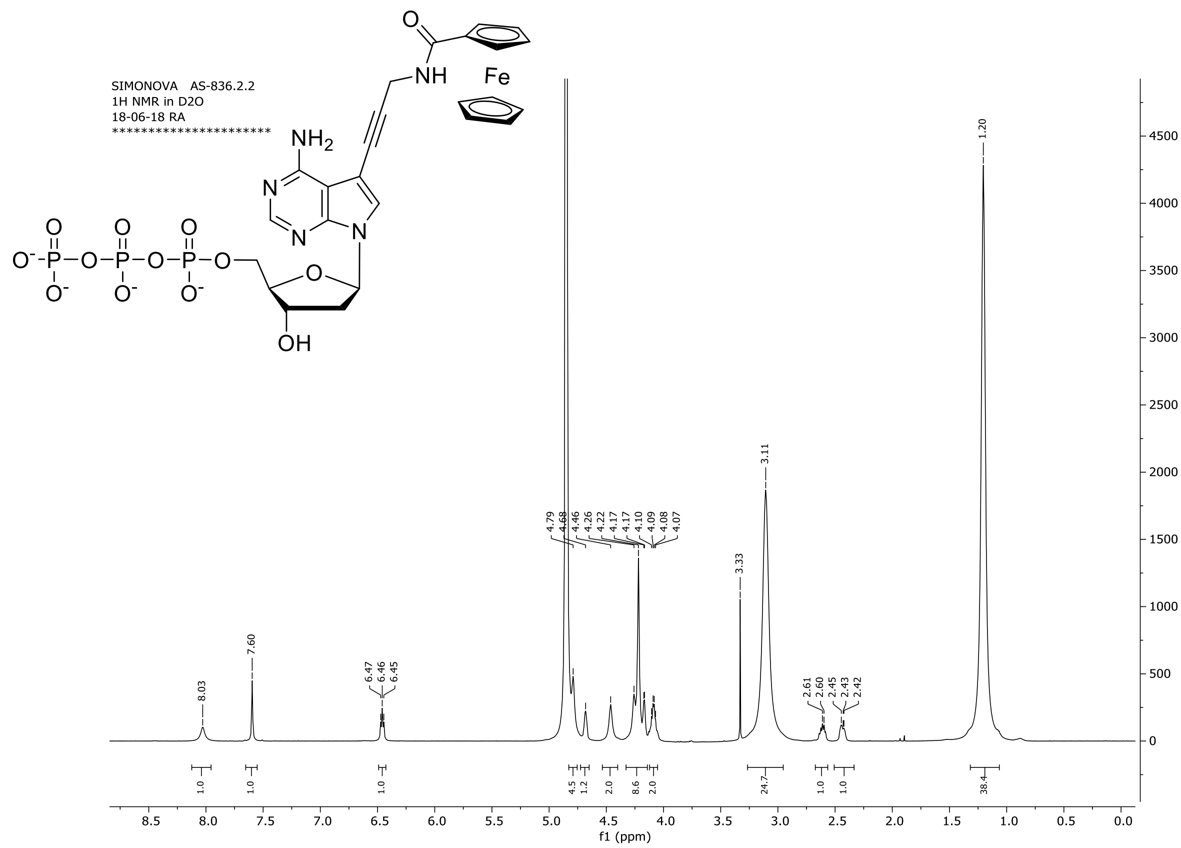

SIMONOVA AS-836.2.2
1H NMR in D2O
18-06-18 RA
****
NH2
N
N
N
O
OH
NH
Fe
O
8.03
7.60
6.47
6.46
6.45
4.79
4.68
4.46
4.26
4.22
4.17
4.17
4.10
4.09
4.08
4.07
3.33
3.11
2.61
2.60
2.45
2.43
2.42
1.20
1.0
1.0
1.0
4.5
1.2
2.0
8.6
2.0
24.7
1.0
1.0
38.4
f1 (ppm)

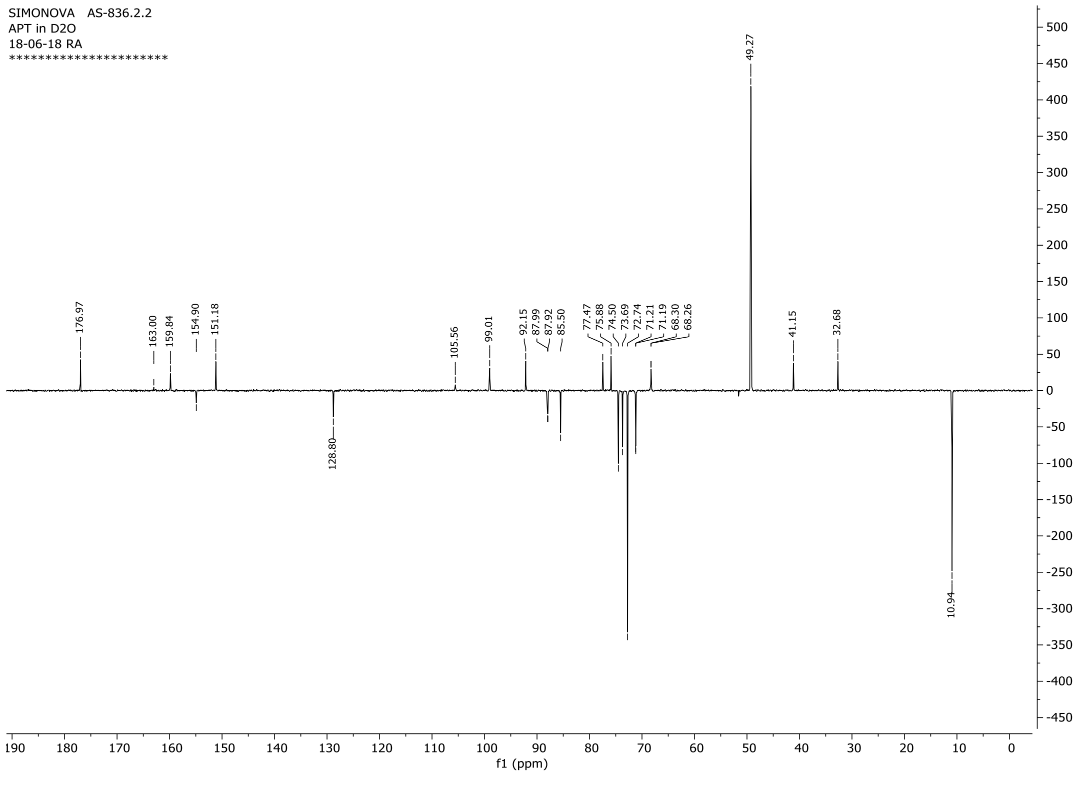

SIMONOVA AS-836.2.2
APT in D2O
18-06-18 RA
****
176.97
163.00
159.84
154.90
151.18
128.80
105.56
99.01
92.15
87.99
87.92
85.50
77.47
75.88
74.50
73.69
72.74
71.21
71.19
68.30
68.26
49.27
41.15
32.68
10.94
f1 (ppm)

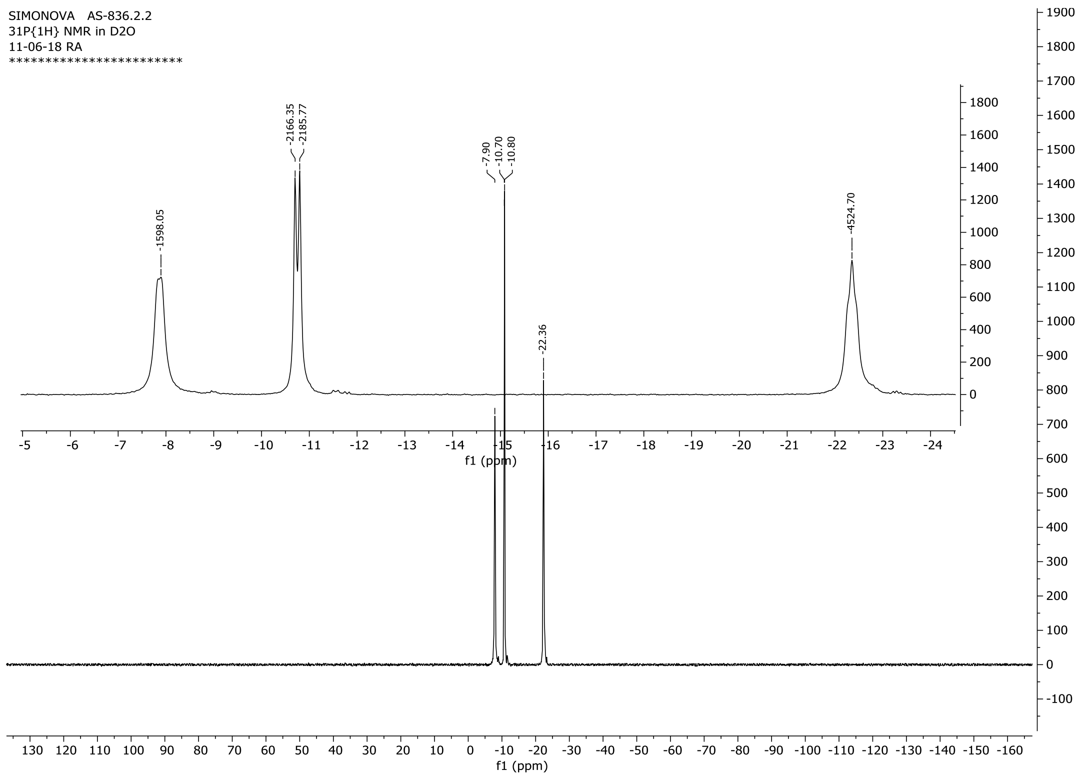
SIMONOVA AS-836.2.2
31P{1H} NMR in D2O
11-06-18 RA
-1598.05
-2166.35
-2185.77
-7.90
-10.70
-10.80
-22.36
-4524.70
f1 (ppm)
f1 (ppm)

dC^{FcPa}TP

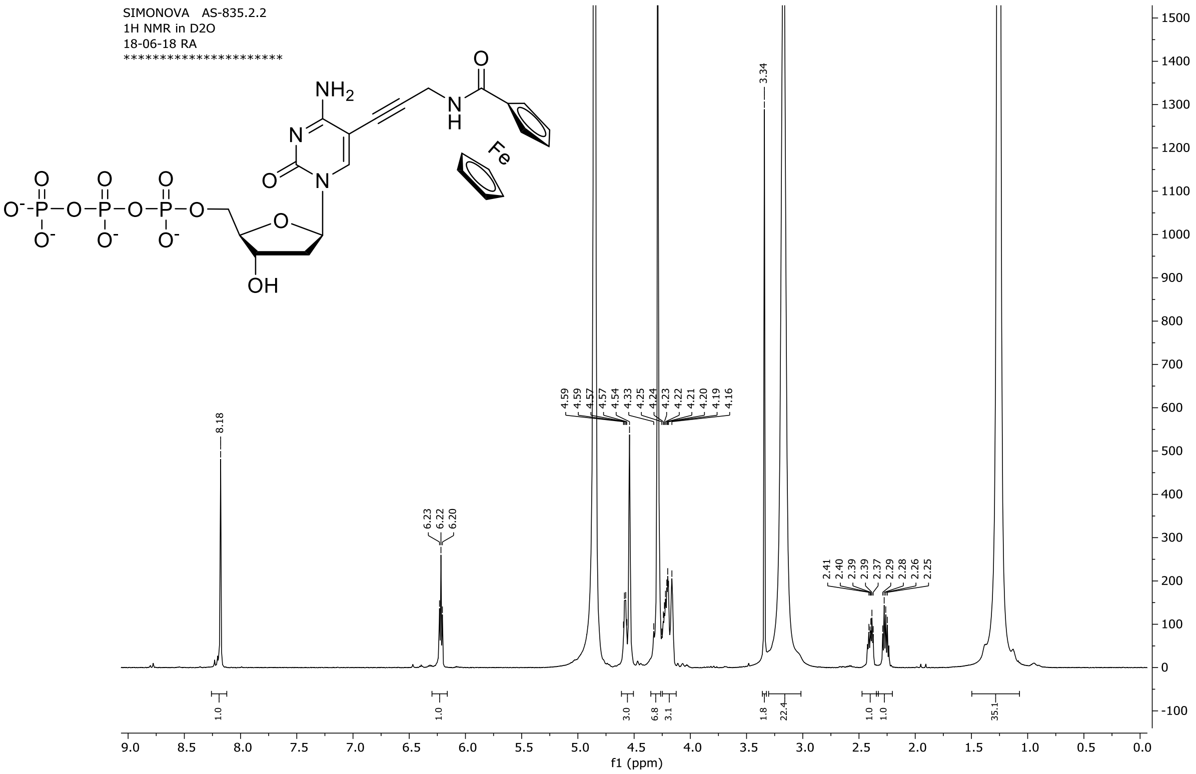
SIMONOVA AS-835.2.2
1H NMR in D2O
18-06-18 RA
NH2
N
O
N
N
H
O
Fe
O
O
O
O-
P
O
P
O
P
O
O-
O-
O-
O-
O
OH
8.18
6.23
6.22
6.20
4.59
4.59
4.57
4.54
4.33
4.25
4.24
4.23
4.22
4.21
4.20
4.19
4.16
3.34
2.41
2.40
2.39
2.39
2.37
2.29
2.28
2.26
2.25
1.0
1.0
3.0
6.8
3.1
1.8
22.4
1.0
1.0
35.1
f1 (ppm)

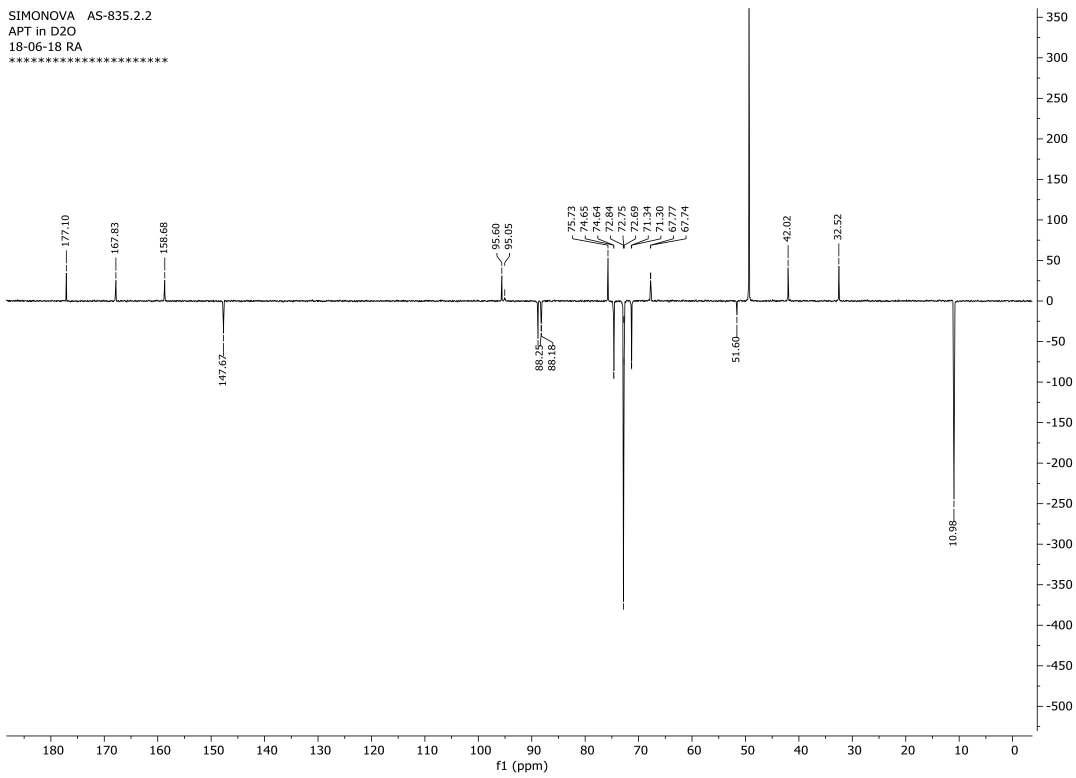
SIMONOVA AS-835.2.2
APT in D2O
18-06-18 RA
f1 (ppm)

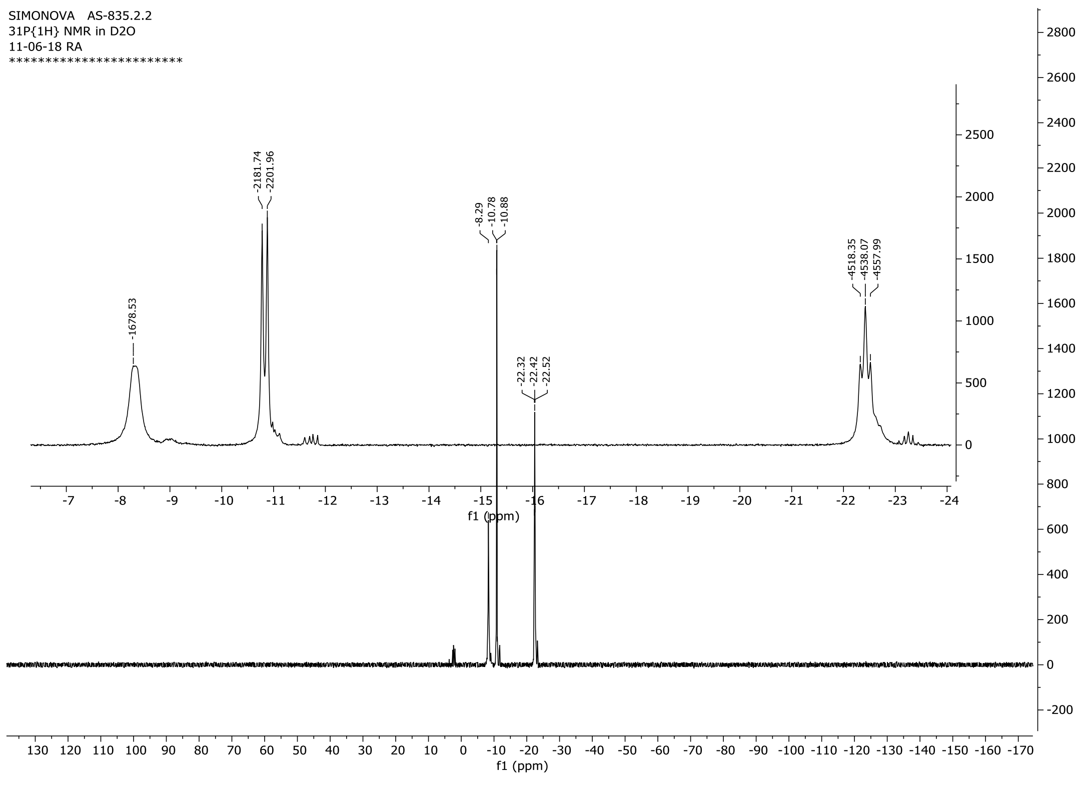
SIMONOVA AS-835.2.2
31P{1H} NMR in D2O
11-06-18 RA
f1 (ppm)

dC^{Fc}TP

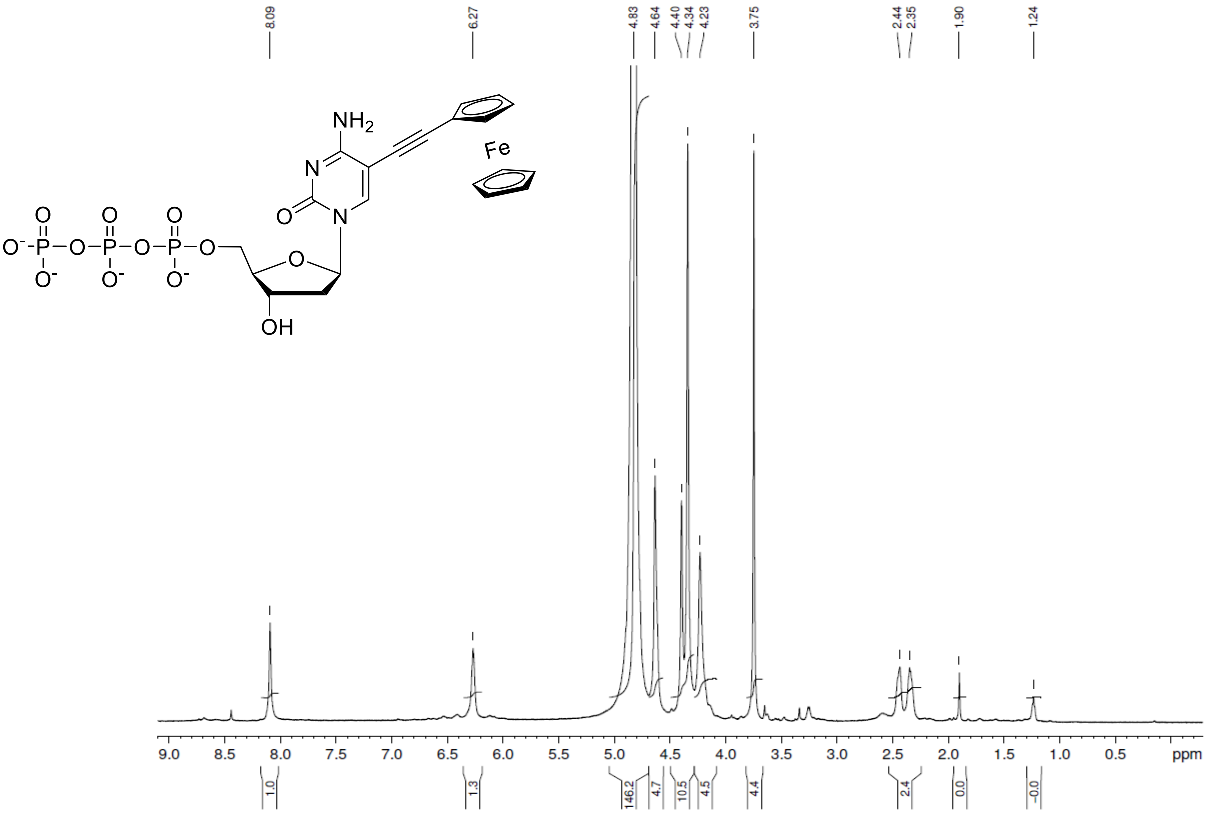

NH2
N
O
N
Fe
O
O
O
O-
P
O
P
O
P
O
O-
O-
O-
O
OH
8.09
6.27
4.83
4.64
4.40
4.34
4.23
3.75
2.44
2.35
1.90
1.24
9.0 8.5 8.0 7.5 7.0 6.5 6.0 5.5 5.0 4.5 4.0 3.5 3.0 2.5 2.0 1.5 1.0 0.5 ppm
1.0
1.3
146.2
4.7
10.5
4.5
4.4
2.4
0.0
-0.0

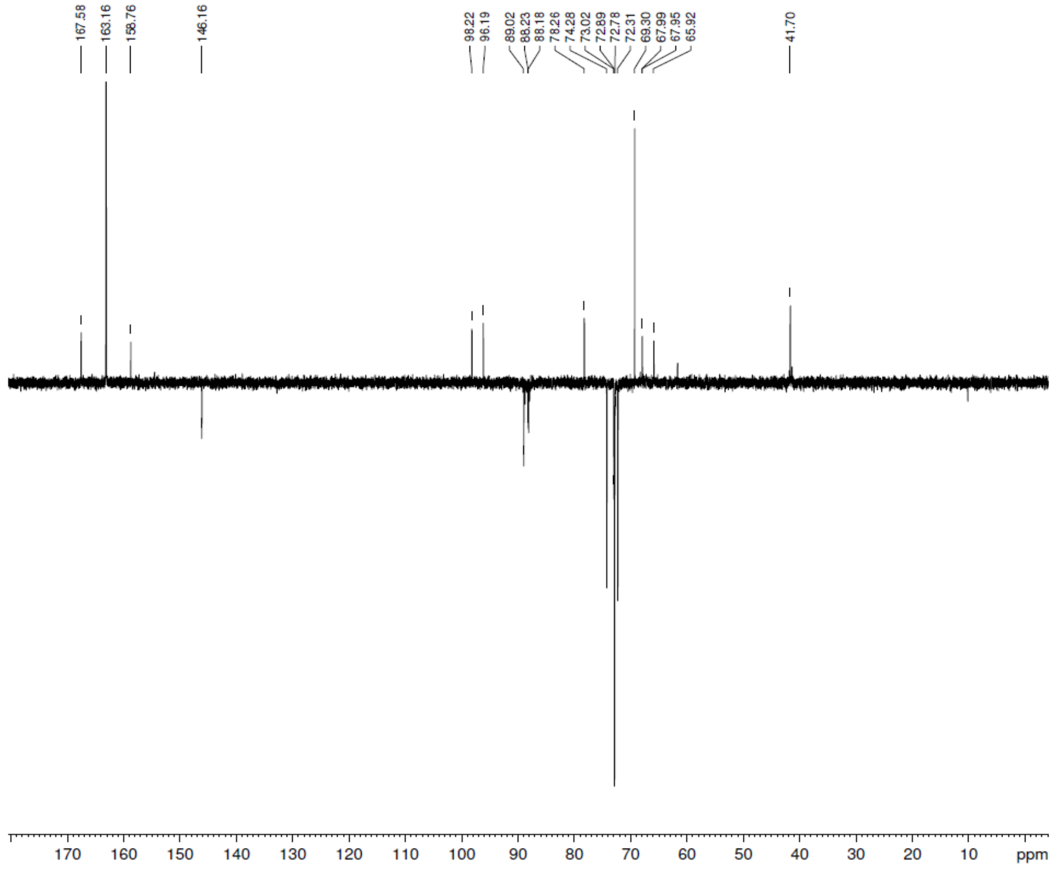

167.58
163.16
158.76
146.16
98.22
96.19
89.02
88.23
88.18
78.26
74.28
73.02
72.89
72.78
72.31
69.30
67.99
67.95
65.92
41.70
170 160 150 140 130 120 110 100 90 80 70 60 50 40 30 20 10 ppm

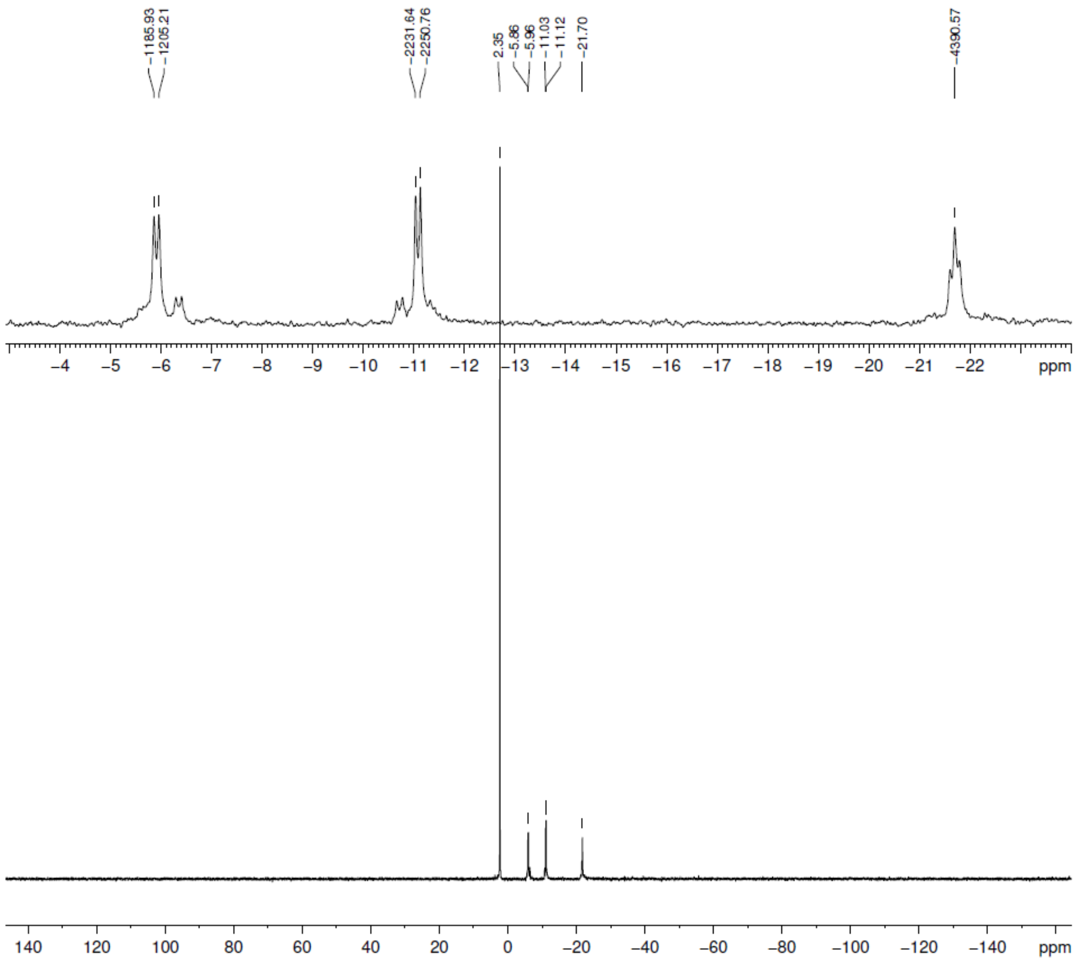

-1185.93
-1205.21
-2231.64
-2250.76
2.35
-5.86
-5.96
-11.03
-11.12
-21.70
-4390.57
ppm

7. References

1. a) S. K. Ghag, M. L. Tarlton, E. A. Henle, E. M. Ochoa, A. W. Watson, L. N. Zakharov, E. J. Watson, *Organometallics*, **2013**, *32*, 1851–1857; b) C. Zou and M. S. Wrighton, *J. Am. Chem. Soc.*, **1990**, *112*, 7578-7584; c) P. Jutzi, B. Kleinebekel, *J. Organometal. Chem.*, **1997**, *545–546*, 573–576.
2. A. E. Beilstein, M. W. Grinstaff, *Chem. Commun.*, **2000**, 509–510.
3. I. Magriñá, A. Toldrà, M. Campàs, M. Ortiz, A. Simonova, I. Katakis, M. Hocek, C.K. O'Sullivan, *Biosens. Bioelectron.*, **2019**, *134*, 76–82.
